# Organic Electrochemical Transistors in Tissue‐Interfaced Bioelectronics

**DOI:** 10.1002/advs.202513422

**Published:** 2025-12-07

**Authors:** Ruixiang Bai, Zeyu Zhao, Feng Yan

**Affiliations:** ^1^ Department of Applied Physics Research Center for Organic Electronics The Hong Kong Polytechnic University Hung Hom Kowloon Hong Kong People's Republic of China

**Keywords:** biocompatibility, biosensors, bio‐tissue interfaces, mechanical compliance, organic electrochemical transistors (OECTs)

## Abstract

Organic electrochemical transistors (OECTs) have attracted considerable attention in the field of tissue‐interfaced bioelectronics. They uniquely bridge the ionic and electronic domains while offering mechanical compliance, biocompatibility, and high signal amplification. These characteristics make them especially advantageous for applications requiring stable, long‐term integration with soft, deformable, and dynamic biological tissues. This review provides a comprehensive summary of recent progress in the design, fabrication, and application of OECTs for biological integration. It begins by outlining the fundamental working mechanisms of OECTs and their advantages in transducing biological signals. Key strategies to enhance bio‐tissue interfacing are discussed, including molecular functionalization, interface orientation control, and mechanical matching. The review also covers developments in OECT‐based systems for skin‐mounted and implantable applications, with a focus on maintaining stability and responsiveness under dynamic physiological conditions. Particular attention is given to approaches that improve biocompatibility, reduce foreign body response, and enable minimally invasive operation in vivo. Finally, the article outlines key remaining challenges and suggests future directions to explore in this area.

## Graphical abstract

This article reviews the design, fabrication, and biological application of organic electrochemical transistors (OECTs), emphasizing their potential in tissue‐interfaced bioelectronics. It covers the fundamental principles of OECTs, strategies for enhancing tissue interfacing, and the development of skin‐mounted and implantable systems. Additionally, it highlights challenges related to biocompatibility and offers insights into future research directions.

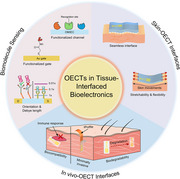

## 1 Introduction

The integration of electronics with biological systems has long been a central goal in bioengineering, driving the development of devices capable of sensing, stimulation, and physiological signal transduction.^[^ ^1^, ^2^, ^3^ ^]^ Traditional diagnostic approaches, which typically rely on intermittent laboratory testing, are often insufficient to capture transient or dynamic physiological signals, limiting their effectiveness in real‐world biomedical applications.^[^ ^4^ ^]^ To overcome these limitations, researchers have focused on developing biosensors and bioelectronic systems that can operate directly within biological environments, enabling more responsive and physiologically relevant interactions.^[^ ^5^, ^6^ ^]^ Luigi Galvani made the seminal discovery of bioelectricity in the 1780s. Since then, extensive efforts have been devoted to developing devices capable of recording and modulating biological signals with high fidelity.^[^ ^7^ ^]^ These bioelectronic platforms directly interface with living tissues and have been extensively explored for applications including disease diagnosis, neural recording, and therapeutic stimulation.^[^ ^8^, ^9^, ^10^, ^11^ ^]^ A key challenge in this domain is the creation of stable and conformable interfaces between bioelectronic devices and soft bio‐tissues, which are essential for high‐quality signal transduction and long‐term functionality.^[^ ^12^ ^]^ Human tissues, such as the epidermis, are soft, deformable, and curvilinear, with a low modulus ranging from 10^5^ to 10^6^ Pa.^[^ ^13^ ^]^ In contrast, conventional electronic systems that employ sub‐millimeter electrodes made from highly conductive noble metals like gold and platinum—featuring extremely high elastic moduli (ranging from 10^9^ to 10^12^ Pa) and rigid, voluminous designs—frequently face challenges when attempting to conform to wearable or implantable applications.^[^ ^14^ ^]^ While noble metals are prized for their chemical stability and low toxicity, they often encounter integration challenges within biological environments.^[^ ^15^ ^]^ A key issue lies in the mechanical disparity between these rigid materials and the soft, compliant nature of human tissues, which can compromise signal fidelity, reduce device longevity, and cause user discomfort such as irritation or inflammation.^[^ ^16^, ^17^ ^]^ Furthermore, although these metals function effectively in dry conditions, they lack adaptive performance in aqueous or physiological settings. Their exclusive conduction of electronic charges stands in contrast to the ionic nature of biological signal transmission, limiting their functionality in bioelectronic interfaces.^[^ ^15^ ^]^ Alternatively, organic semiconductors offer significant potential for establishing effective communication between electronic systems and biological entities.^[^ ^18^ ^]^ Among these, organic mixed ionic‐electronic conductors (OMIECs) represent a class of organic semiconductors that exhibit intrinsic compatibility with aqueous environments. OMIECs typically consist of conjugated polymers with customized chemical structures, enabling them to achieve conductivity through ion interactions while allowing body fluids to serve as an integral component.^[^ ^7^, ^18^ ^]^ Additionally, as polymeric materials, OMIECs possess inherent flexibility that enables adaptation to various form factors and facilitates manufacturing through diverse processing techniques. Their properties can be further engineered via backbone modifications, side‐chain engineering, or blending with complementary materials to create flexible, stretchable, and deformable devices. These combined properties establish OMIECs as ideal electronic materials for biointerface applications.^[^ ^19^, ^20^, ^21^, ^22^ ^]^

**Figure 1:**
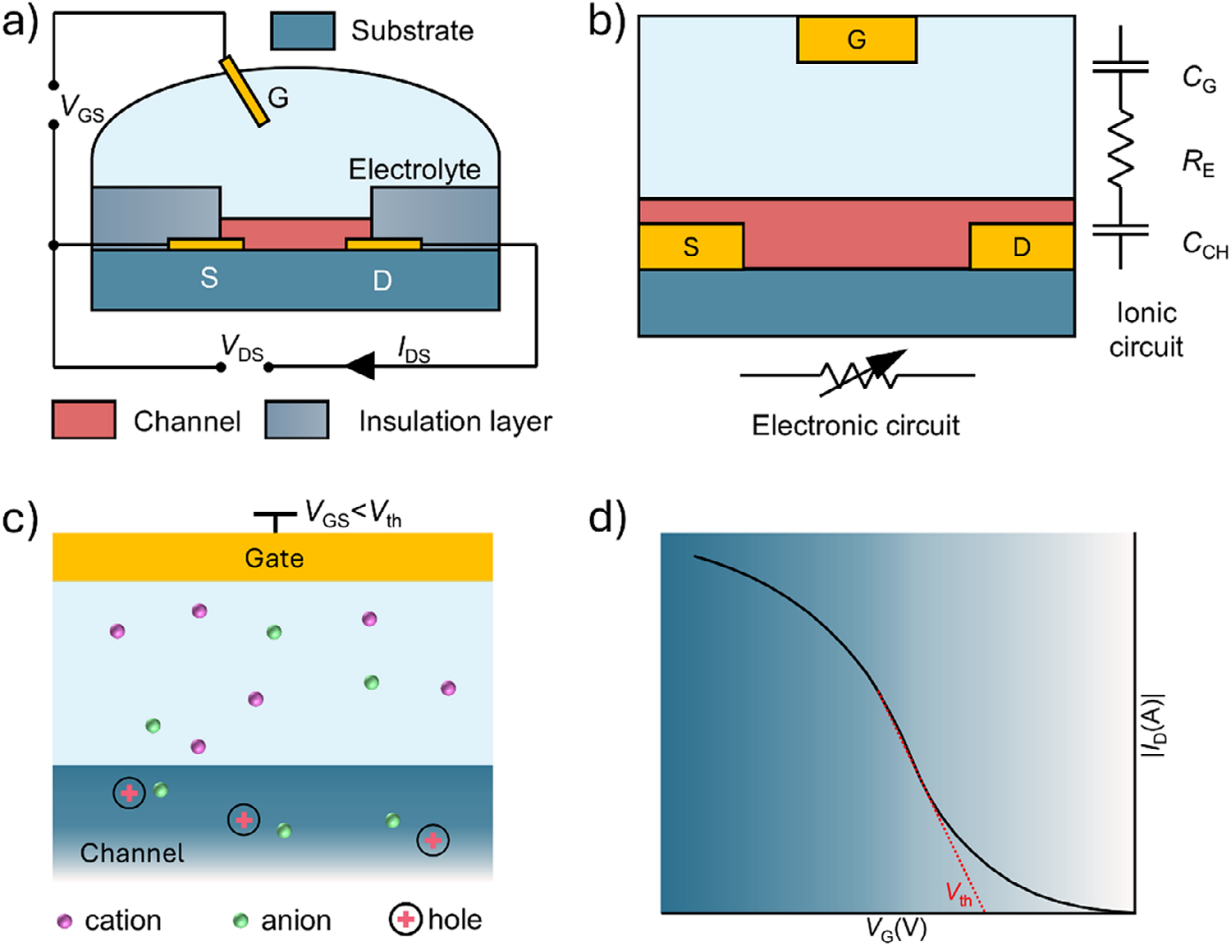
Structure and operation of a typical organic electrochemical transistor (OECT). a) Structural components of a typical OECT, including source (S), drain (D), gate (G), and electrolyte. b) Schematic representation of the electronic and ionic pathways in an OECT. c) Typical structure of an OECT (p‐channel device as an example). d) The transfer curve of a typical p‐type accumulation‐mode OECT.

Organic electrochemical transistors (OECTs) harness the above functionalities of OMIECs, offering unique advantages in tissue‐interfaced bioelectronics. OECTs combine mechanical flexibility, biocompatibility, and signal amplification capabilities, making them ideal for direct integration with biological tissues.^[^ ^7^, ^23^ ^]^ First introduced by White et al. in the 1980s,^[^ ^24^ ^]^ OECTs constitute a distinct type of organic thin‐film transistor (OTFT), uniquely suited for bioelectronic applications owing to their reliable performance in physiological environments. Like organic field‐effect transistors (OFETs), OECTs are three‐terminal devices with a gate, source, and drain (**Figure** **1**a). However, unlike OFETs, where current modulation occurs at the interface between the organic semiconductor and the dielectric, OECTs allow ions from the electrolyte to penetrate the organic semiconductor, modulating its doping state throughout the entire channel. This ion‐to‐electron coupling mechanism significantly enhances conductivity and enables OECTs to achieve greater drain current modulation at low gate voltages, making them ideal for amplifying bioelectric signals and detecting biomarkers with high sensitivity.^[^ ^25^, ^26^, ^27^, ^28^ ^]^ Furthermore, the sensing mechanism of OECTs depends on direct interfacing between the semiconductor channel and biological tissue, where biopotentials or specific biochemical cues modulate the channel's bulk conductivity through electrostatic interactions.^[^ ^29^, ^30^, ^31^ ^]^ As this transduction process is inherently sensitive to the nanoscale proximity between the channel and the tissue surface,^[^ ^32^ ^]^ achieving an optimal interface necessitates intimate and conformal adhesion of the semiconductor to the target tissue.^[^ ^12^ ^]^

There has been growing interest in OECTs for bio‐tissue engineering, driven by advances in materials science, fabrication miniaturization, and the increasing demand for real‐time, localized sensing in dynamic biological environments.^[^ ^33^ ^]^ OECTs exhibit a unique combination of ionic–electronic coupling, low‐voltage operation, and mechanical softness, making them well‐suited for bioelectronic platforms that require intimate integration with soft tissues.^[^ ^15^, ^34^ ^]^ These attributes have facilitated progress in biosensing, stimulation, and tissue monitoring. However, challenges persist in translating laboratory‐scale performance into robust, scalable technologies suitable for clinical deployment. OECTs are also being actively investigated for their potential in neuromorphic bioelectronics, where devices replicate neural behaviors such as signal integration, threshold‐triggered spiking, and temporal coding.^[^ ^35^, ^36^ ^]^ For example, organic electrochemical neurons (OECNs) based on OECTs have been shown to replicate biological spiking dynamics through ion‐mediated transduction, enabling event‐based sensing, adaptive biointerfaces, and decentralized computation at the tissue‐electronic interface.^[^ ^37^ ^]^ These neuromorphic OECTs present key advantages over silicon‐based systems, including intrinsic biocompatibility, multimodal sensing, and stretchable architectures.^[^ ^38^ ^]^ As such, they represent a compelling direction for next‐generation OECT‐based platforms that integrate both biosensing and bioprocessing functionalities.

In this review, we comprehensively examine the role of OECTs in advancing tissue‐interfaced bioelectronics. We begin by introducing the fundamental working mechanisms of OECTs, highlighting their distinct ionic–electronic coupling and high transconductance that enable sensitive signal amplification in physiological environments. Building upon this foundation, we delve into strategies for engineering the bio‐tissue interface to achieve long‐term functional integration. These include biochemical functionalization of the channel and gate to enhance molecular specificity, as well as consideration of orientation effects and Debye length (λ_D_) constraints at the interface. We then discuss the development of skin‐integrated OECTs, focusing on structural innovations that promote seamless adhesion and stable operation under mechanical deformation. Furthermore, we explore in vivo applications of OECTs, addressing key challenges related to biocompatibility, bioresorbability, and minimal invasiveness required for implantable use. Finally, we outline critical considerations and unresolved challenges that must be addressed to translate these technologies toward real‐world biomedical applications.

## 2 Working Mechanism of OECTs

### 2.1 Device Operation Mechanism

OECTs can transduce a small gate voltage into a pronounced modulation of the drain current, effectively enabling signal amplification.^[^ ^25^ ^]^ This transduction capability is characterized by the transfer curve, which represents the relationship between the drain current (*I*_DS_) and gate voltage (*V*_GS_). The steeper the slope of the transfer curve, the more effective the modulation at that gate voltage, as indicated by the large rate of change in the drain current.^[^ ^7^, ^23^ ^]^ This phenomenon, termed the amplification capability of OECTs, is quantified by the transconductance (gm=∂IDS/∂IDS∂VGS∂VGS), a key performance parameter of transistors. OECTs exhibit remarkably high transconductance, often reaching millisiemens in micrometer‐scale devices, which is attributed to their distinctive structural configuration and electrochemical operating mechanism.^[^ ^25^ ^]^ In practical bioelectronic applications, however, amplification alone does not guarantee effective signal detection; a high signal‐to‐noise ratio (SNR) is equally critical. SNR, typically expressed in decibels (dB), quantifies the ability to distinguish biological signals from background noise and is calculated as:

(1) $$\mathrm{SNR}[\mathrm{dB}]=20\mathrm{lo}g_{10}\left(\frac{A_{\mathrm{signal}}}{A_{\mathrm{noise}}}\right)$$

where *A*_signal_ is the peak amplitude of the detected biosignal (e.g., ECG), and *A*_noise_ is the standard deviation of the background. A higher SNR indicates a cleaner signal and is essential for evaluating OECT performance in real‐world sensing environments.

The fundamental operating mechanism of OECTs is well explained by the model introduced by Bernards, which integrates the ionic and electronic circuits, as depicted in Figure 1b).^[^ ^26^ ^]^ According to the model's assumptions, ions in the electrolyte can penetrate the channel and change its volumetric electronic conductivity. This process allows the semiconductor to undergo doping and de‐doping, capturing both transient and steady‐state responses. The model conceptualizes the OECT as comprising two primary components: the ionic circuit and the electronic circuit. The ionic circuit governs ion transport through the gate, electrolyte, and channel, modulating the channel's properties. Meanwhile, the electronic circuit describes the movement of charge carriers between the source, channel, and drain, following Ohm's law. Within the ionic pathway, the organic channel functions as a variable resistor whose conductivity is regulated by ionic doping. This circuit can be modeled as a resistor–capacitor (RC) circuit, comprising a resistor that represents ion flow in the electrolyte and a capacitor that accounts for ion storage in the channel. For devices with polarized gate electrodes (e.g., Au or Pt), the ionic circuit includes an electrostatic double‐layer (EDL) capacitor (*C*_G_) formed at the gate/electrolyte interface and another capacitor (*C*_CH_) related to the channel's volumetric capacitance, both connected in series. Efficient gating is achieved when the gate capacitance (*C*_G_) is much larger than the channel capacitance (*C*_CH_), ensuring that most of the potential drop facilitates ion injection into the channel.^[^ ^39^ ^]^ This model represents the gating mechanism as a purely electrostatic (capacitive) process at the device level, where ion redistribution induces electronic modulation without redox reactions. It captures the charge compensation dynamics using an equivalent capacitor network, but this simplification does not negate the intrinsic volumetric ion penetration and charge modulation that characterize OECT operation. The ions injected into the channel do not participate in ion exchange or chemical reactions with the organic polymer. Instead, they induce opposite charges in the channel via electrostatic compensation.^[^ ^25^ ^]^

The operational modes of OECTs are categorized into depletion mode and accumulation mode.^[^ ^25^ ^]^ Depletion‐mode devices exhibit high conductivity at zero gate bias, often due to self‐doping. Applying a positive gate voltage (*V*_GS_) induces electrochemical dedoping, whereby ionic compensation reduces hole density and lowers channel conductivity. In contrast, accumulation‐mode OECTs exhibit low conductivity at zero voltage. Application of gate voltage promotes doping, increases charge carrier concentration, and enhances conductivity.^[^ ^40^ ^]^ For example, in a p‐type accumulation‐mode OECT, charge transport is dominated by hole carriers. At zero gate voltage, the channel is weakly conductive. When a negative gate voltage (*V*_GS_ < *V*_th_) is applied, as illustrated in Figure 1c, anions from the electrolyte migrate into the channel, resulting in hole accumulation and increased channel conductivity (Figure 1d). At saturation, the output characteristics of accumulation‐mode devices can be well‐fitted by the Bernards model, and the transconductance (*g*_m_) can also be predicted by this model, expressed as^[^ ^26^ ^]^:

(2) $$g_{m}=\left(\frac{W}{L}\right)\cdot d\cdot \mu \cdot C^{*}\cdot \left|V_{GS}-V_{th}\right|$$

where *L* and *W* represent the channel length and width, respectively, while *d* denotes the channel thickness. The electronic mobility is given by *µ*, and *C* ^*^ refers to the volumetric capacitance of the channel. The threshold voltage is indicated by *V*_th_. In OECTs, the product *µC* ^*^ characterizes bulk doping, and due to the exceptionally high capacitance *C* ^*^ of organic mixed conductors, OECTs exhibit significantly higher transconductance compared to both traditional FETs and their counterparts, OFETs. Furthermore, the model suggests that the transconductance of OECTs depends on the channel geometry (*Wd*/*L*) and the performance metrics of the active layer (*µC* ^*^), providing a framework for optimizing device design and selecting or developing appropriate active layer materials.^[^ ^23^ ^]^

Although OECTs exhibit extremely high transconductance, their operation tends to be relatively slow. As described by the Bernards model, the response time of OECTs is primarily dictated by either the RC time constant associated with the ionic circuit or the dynamics of the electronic transport pathway.^[^ ^26^ ^]^ In most devices, ion movement within the electrolyte is considerably slower than the charge carrier transfer in the electronic circuit. However, recent progress in microfabrication has facilitated the creation of compact mixed‐conducting materials with enhanced charge carrier mobility, thereby markedly shortening the transit time (τ_e_) of carriers within the channel (τe≈L2/L2μVDSμVDS).^[^ ^7^ ^]^ The RC time constant (τ_i_) in the ionic pathway is determined by the resistance of the electrolyte (*R*_E_) and the capacitance associated with the channel (*C*_CH_), given by τ_i_ = *C*_CH_ · *R*_E_.^[^ ^26^ ^]^ The capacitance of the channel (*C*_CH_) is proportional to the channel volume *WdL*, i.e.,*C*_CH_∝*WdL*.^[^ ^41^ ^]^ On the other hand, the electrolyte resistance (*R*_E_), a key factor affecting the time constant, adheres to a specific scaling relationship:RE∝1/1WLWL.^[^ ^42^, ^43^ ^]^ From the scaling of these two parameters, it can be deduced that the time constant τ_i_ is proportional to $d\sqrt{WL}$.^[^ ^44^ ^]^ This relationship indicates that the response speed of OECTs can be tuned by altering the device geometry. It is important to note that there is a trade‐off between the modulation performance (*g*_m_) and response speed (τ_i_) in OECTs.^[^ ^7^, ^23^, ^25^ ^]^ Reducing the channel thickness (*d*) can improve the response speed but at the expense of *g*_m_. In practical applications, current OECTs employing liquid electrolytes exhibit response times on the order of tens of microseconds, supporting frequencies up to tens of kilohertz. This performance is sufficient for recording and sensing most electrophysiological signals.^[^ ^25^ ^]^ Although these parameters are of vital importance, they cannot completely determine the detection performance of the device. The device's transducing and amplifying performance is governed by its intrinsic physical characteristics as well as its interfacial interactions with the surrounding biological environment.^[^ ^15^ ^]^

### 2.2 Organic Mixed Ionic–Electronic Conductors (OMIECs)

OMIECs represent a critical class of materials that enable the dual transport of electronic and ionic species within OECTs.^[^ ^25^ ^]^ The ability of OMIECs to accommodate bulk ion penetration and modulate charge transport under low‐voltage operation makes them uniquely suited for applications in bioelectronics, neuromorphic systems, and soft robotics. Over the past decade, the OMIEC landscape has rapidly diversified in both material chemistry and device engineering strategies. Central to the performance evaluation of OMIECs is the figure of merit µC*, defined as the product of carrier mobility (µ) and volumetric capacitance (C*), which reflects the material's mixed conduction efficiency under electrochemical gating. This section reviews recent advances in p‐type, n‐type, and composite OMIEC systems. **Table** **1** summarizes recent advancements in p‐type, n‐type, and ambipolar channel materials reported between 2024 and 2025, while additional materials can be found in our earlier review.^[^ ^7^ ^]^

**Table 1:** Summary of OMIECs.

OMIEC	Type	Wd/L [µm]	*g*_m_ [µS]	*g*_m_/(Wd/L) [S cm^−1^]	µC* [F V^−1^ cm^−1^ s^−1^]	C* [F cm^−3^]	µ [cm^2^ V^−1^ s^−1^]	τ_on_ [ms]	(year) ref.
p(g3T2‐O)	p	NA	NA	14 ± 5	29 ± 5	170 ± 15	0.21 ± 0.03	NA	(2024)^[^ ^45^ ^]^
p(g3T2‐S)	p	NA	NA	122 ± 43	223 ± 56	208 ± 30	1.38 ± 0.17	NA	
p(g3T2‐Se)	p	NA	NA	33 ± 17	64 ± 18	99 ± 5	0.92 ± 0.22	NA	
p(g3T2‐Te)	p	NA	NA	214 ± 159	462 ± 264	134 ± 15	3.60 ± 1.92	NA	
PE_2_‐OE_4_	p	0.020–0.312	∼ 1200–15400	453 ± 70	830 ± 37	207 ± 19	4.01 ± 0.56	16.9	(2025)^[^ ^54^ ^]^
PE_2_‐OE_6_	p	0.055–0.585	∼ 600–7500	111 ± 13	239 ± 7	92 ± 14	2.60 ± 0.52	12.8	
PE_2_‐OE_2_OE_2_	p	0.048–0.368	∼ 600–10000	235 ± 34	486 ± 11	138 ± 19	3.52 ± 0.75	7.6	
PE_2_‐OE_2_OE_3_	P	0.034–0.260	∼ 700–4500	292 ± 42	604 ± 23	149 ± 25	4.05 ± 1.08	15.8	
PE_2_‐OE_3_OE_3_	p	0.039–0.189	∼ 500–5000	221 ± 73	511 ± 27	143 ± 20	3.57 ± 0.86	13.6	
i‐3gTIT	p	NA	NA	86.2	302	169	1.80	NA	(2025)^[^ ^47^ ^]^
o‐3gTIT	p	NA	NA	6.7	27	130	0.21	NA	
gFBT‐g2T	p	0.329	98.042 ± 3.422	298 ± 10.4	847 (826 ± 28.8)	147 ± 10	5.76	19	(2025)^[^ ^46^ ^]^
gFBT‐3g2T	p	0.401	17.44 ± 0.56	43.5 ± 1.4	202 (198 ± 6.4)	292 ± 14	0.69	23	
DHF‐gTT	ambipolar	2.4	10.01 ± 1.13 ^a)^	4.17 ± 0.47 ^a)^	14.0 ± 0.6 ^a)^	104.8 ± 7.9 ^a)^	0.134 ± 0.015 ^a)^	5.87 ^a)^	(2025)^[^ ^51^ ^]^
			6.24 ± 0.22 ^b)^	2.60 ± 0.09 ^b)^	11.9 ± 0.5 ^b)^	41.9 ± 3.9 ^b)^	0.284 ± 0.035 ^b)^	38.75 ^b)^	
DH‐gTT	ambipolar	2.8	3.64 ± 0.20 ^a)^	1.30 ± 0.07 ^a)^	5.4 ± 0.2 ^a)^	134.6 ± 7.9 ^a)^	0.040 ± 0.004 ^a)^	5.32 ^a)^	
			0.64 ± 0.17 ^b)^	0.23 ± 0.06 ^b)^	5.6 ± 0.3 ^b)^	71.8 ± 3.2 ^b)^	0.078 ± 0.008 ^b)^	41.32 ^b)^	
Pg3Tz‐2‐DPP	n	NA	NA	4.8 ± 0.4	14.2 ± 0.9	243.6 ± 18.2	0.058 ± 0.071	41	(2025)^[^ ^55^ ^]^
Pg3Tz‐5‐DPP	n	NA	NA	8.6 ± 0.4	22.4 ± 1.7	246.5 ± 9.8	0.091 ± 0.037	33	
Pg4Tz‐5‐DPP	n	NA	NA	18.6 ± 1.2	47.2 ± 2.1	253.2 ± 9.6	0.186 ± 0.032	66	
Pg5Tz‐5‐DPP	n	NA	NA	30.8 ± 1.1	92.5 ± 3.8	243.3 ± 1.5	0.380 ± 0.01	46	
Pg6Tz‐5‐DPP	n	NA	NA	7.5 ± 0.5	23.6 ± 1.3	246.2 ± 9.0	0.096 ± 0.034	48	
Pg7Tz‐5‐DPP	n	NA	NA	4.9 ± 0.1	16.6 ± 1.9	252.8 ± 6.0	0.066 ± 0.022	23	
p(N‐T):PS 10K 1:6	n	2.14	0.064 ± 0.010	0.030 ± 0.0048	13.4 ± 2.8	10.1 ± 0.9	1.3 ± 0.3	2.90	(2024)^[^ ^49^ ^]^
p(N‐T):PMM 10K 1:6	n	4.12	0.024 ± 0.005	0.0059 ± 0.0012	5.3 ± 1.2	9.0 ± 0.5	0.6 ± 0.1	1.40	
p(N‐T):PS 1K 1:6	n	3.76	0.046 ± 0.012	0.0123 ± 0.0032	4.9 ± 1.9	7.9 ± 1.0	0.6 ± 0.2	1.81	
p(1gNDI‐T2)	n	NA	NA	0.0074 ± 0.0005	30.8 ± 2.0	235.5 ± 25.5	1.29 ± 0.22×10^−4^	368.4 ± 33.6	(2024)^[^ ^56^ ^]^
p(2gNDI‐T2)	n	NA	NA	0.030 ± 0.0048	166.5 ± 26.6	168.4 ± 10.6	9.83 ± 2.19×10^−4^	99.2 ± 0.1	
p(3gNDI‐T2)	n	NA	NA	0.0123 ± 0.0032	76.0 ± 26.6	156.5 ± 10.8	4.76 ± 1.58×10^−4^	107.5 ± 30.5	
p(4gNDI‐T2)	n	NA	NA	0.0059 ± 0.0012	73.7 ± 14.9	134.3 ± 10.5	5.17 ± 1.45×10^−4^	97.2 ± 0.5	
PBFDO	n	0.14	61.32	438	796	NA	NA	0.138	(2024)^[^ ^48^ ^]^
PBFDO‐PEG50wt%	n	0.14	57.68	412	724	NA	NA	0.072	
PBFDO‐PEG100wt%	n	0.14	37.1	265	583	NA	NA	0.022	

#### 2.2.1 p‐Type OMIECs

The development of high‐performance p‐type OMIECs has progressed substantially, driven by insights into how molecular architecture governs ion‐electron coupling. A central strategy involves balancing hydrophilicity and structural order to promote both C* and µ. For instance, Paulsen et al.^[^ ^45^ ^]^ introduced chalcogenophene units into the polymer backbone to enhance π–π stacking and hydration, resulting in OMIECs with improved morphological order and mixed conduction. This backbone engineering strategy yielded devices with µC* values exceeding 200 F·cm^−1^·V^−1^·s^−1^, demonstrating a promising pathway for boosting p‐type performance through structural design. Similarly, a representative example is the PEDOT‐based copolymer PE_2_‐OE_4_, developed by Bardagot et al., which balances the electroactive thiophene backbone with optimized oligo(ethylene glycol) (OE) side chains. This material achieves a µC* of 830 ± 37 F·cm^−1^·V^−1^·s^−1^, and exhibits remarkable operational stability, retaining 99% of its drain current over 200 ON/OFF cycles in aqueous environments.

In the context of medical bioelectronics, Liao et al.^[^ ^46^ ^]^ introduced a dioxythiophene–phthalimide‐based polymer (gFBT‐g2T) engineered for sterilization‐resilient operation. This polymer integrates branched glycol side chains and a robust donor–acceptor backbone to enable high ionic and electronic transport, even after autoclave treatment at 121 °C for 20 minutes. The material maintained its µ of 5.76 cm^2^·V^−1^·s^−1^, C* of 147 ± 10 F·cm^−3^, and a µC* of 847 F·cm^−1^·V^−1^·s^−1^, among the highest reported for D–A OMIECs. In parallel, degradability and bio‐integration have emerged as frontiers in OMIEC design. Chen et al.^[^ ^47^ ^]^ introduced a novel degradable p‐type OMIEC, i‐3gTIT, incorporating imine linkages along a fused heteroaromatic backbone. This polymer combined high mixed conduction—µ = 1.99 cm^2^·V^−1^·s^−1^, µC* = 302 F·cm^−1^·V^−1^·s^−1^—with hydrolytic degradability in mildly acidic aqueous environments over several days. Furthermore, it enabled high‐gain complementary OECT inverters with gain = 31.6 V/V at 0.6 V supply, and artificial synapses with > 90% *Modified National Institute of Standards and Technology* (MNIST) recognition accuracy when used in neuromorphic circuits. Its ability to undergo full or partial degradation post‐operation makes it a landmark material for transient, secure, and bioresorbable electronics.

#### 2.2.2 n‐Type OMIECs

The advancement of n‐type OMIECs has historically been hindered by limited stability and electron mobility under aqueous or ambient conditions. Recent materials have overcome these limitations through backbone and side‐chain design. For instance, Tang et al.^[^ ^48^ ^]^ incorporated polyethylene glycol (PEG) side chains into PBFDO, a highly conductive n‐type polymer, yielding variants such as PBFDO‐PEG50wt% with a µC* exceeding 720 F·cm^−1^·V^−1^·s^−1^, while maintaining a transient response time as low as 72 µs. Wu et al. synthesized a high‐performing selenophene‐substituted polymer, f‐BSeI2g‐SVSCN, which achieved an electron mobility (µ_e_, OECT) of 0.48 cm^2^·V^−1^·s^−1^, C* of 387 F·cm^−3^, and a record µC* of 191.2 F·cm^−1^·V^−1^·s^−1^. These gains are attributed to intensified π–π stacking and improved film crystallinity. Devices maintained 90% of their current after 30 min continuous bias, demonstrating robust aqueous‐phase operational stability.

To enhance morphological uniformity and suppress trap states, Zeglio et al.^[^ ^49^ ^]^ introduced a dilution strategy, blending the active n‐type polymer p(N‐T) with polystyrene (PS) at a 1:6 ratio. This significantly increased µ from 0.059 to 1.3 cm^2^·V^−1^·s^−1^, and µC* from 4.3 to 13.4 F·cm^−1^·V^−1^·s^−1^, while improving current retention from 12% to 77% over 60 min, evidencing that inert matrix support enhances both film uniformity and electrochemical resilience. Despite these material advances, oxygen reduction reactions (ORRs) remain a key degradation pathway. Nayak et al.^[^ ^50^ ^]^ systematically evaluated ORR behavior across multiple n‐type OMIECs and concluded that the lowest unoccupied molecular orbital (LUMO) levels alone are insufficient predictors of oxygen sensitivity. Instead, specific backbone chemistry and redox site identity dominate ORR reactivity and electrochemical cycling stability. Their findings urge future materials design to prioritize O_2_‐inert chemical motifs rather than LUMO tuning alone.

#### 2.2.3 Composite OMIECs

Composite OMIECs, which combine two or more materials to synergistically enhance ionic/electronic transport or introduce new functionalities, have emerged as a versatile strategy to overcome the trade‐offs inherent in single‐component materials. Recent composites focus on blending semiconducting polymers with insulating or functional additives or constructing heterostructures for ambipolar operation.

Qi et al.^[^ ^51^ ^]^ developed a single‐component ambipolar polymer (DHF‐gTT) by copolymerizing oligoethylene glycol‐functionalized bithiophene (donor) and fluorinated bisisatin‐lactone (acceptor) units. This composite exhibited balanced ionic–electronic transport, with µC* values of 14.0 ± 0.6 F·cm^−1^·V^−1^·s^−1^ (n‐type) and 11.9 ± 0.5 F·cm^−1^·V^−1^·s^−1^ (p‐type), alongside hole and electron mobilities of 0.284 ± 0.035 cm^2^·V^−1^·s^−1^ and 0.134 ± 0.015 cm^2^·V^−1^·s^−1^, respectively. This balance of charge transport properties enabled the successful fabrication of ambipolar OECTs, which exhibited a high voltage gain of 102 V/V, and simultaneously allowed real‐time detection of histamine and hydrogen peroxide, showcasing its biosensing capabilities in a single material platform.

Building upon this concept of ambipolar behavior, Wu et al.^[^ ^52^ ^]^ introduced a rigid ladder polymer, Cl_2_‐BAL, based on a poly(benzimidazoanthradiisoquinolinedione) backbone. Unlike DHF‐gTT, which relies on side‐chain functionalization to optimize ion and electron transport, Cl_2_‐BAL utilizes its planar, rigid structure to enhance electronic mobility and resist swelling during electrochemical cycling. This design results in a µC* of 6.8 F·cm^−1^·V^−1^·s^−1^, and normalized transient response times as fast as 0.56 ± 0.17 ms·µm^−2^, making it one of the fastest and most stable n‐type materials for OECTs. Importantly, Cl_2_‐BAL demonstrates antiambipolar behavior, enabling dynamic reconfiguration of logic functions, such as AND, NOR, OR, and NAND, within a single transistor, a property that opens up new avenues for reconfigurable bioelectronics. Similarly, Lan et al.^[^ ^53^ ^]^ reported an ultrathin‐film composite of small‐molecule OMIECs with ion‐tunable ambipolarity. In this case, the D‐A small molecule was blended with a glycolated additive to achieve ultrathin thicknesses of 12 nm, while maintaining µC* values of 45 F·cm^−1^·V^−1^·s^−1^ (p‐type) and 38 F·cm^−1^·V^−1^·s^−1^ (n‐type). By tuning the ionic strength of the electrolyte, the additive modulated ion penetration depth, enabling reversible switching between ambipolar and unipolar operation. This flexibility enabled the material to be integrated into flexible OECT arrays for neuromorphic computing, demonstrating spike‐timing‐dependent plasticity (STDP) with low power consumption (<10 pJ per spike).

## 3 Bio‐Tissue Interfaces in Bioelectronics

### 3.1 Mechanical Properties of Biological Tissues and Interface Requirements

The interface with biological tissues is essential in sensing applications, as it enables precise detection and analysis of physiological signals within the body.^[^ ^12^ ^]^ With the continuous advancement of material science, micro‐nano manufacturing technologies, and biomedical engineering, various novel bioelectronic materials and devices have emerged, providing abundant options for research and application of biological tissue interfaces. In particular, compared to currently clinically approved technologies, bioelectronic devices with superior biological integration and higher spatiotemporal resolution had already completed concept validation over a decade ago, paving the way for novel directions in bioelectronic integration.^[^ ^57^ ^]^ For example, Viventi et al. developed a novel silicon‐based electronic platform capable of conformal adhesion to biological tissues and integrating 2016 silicon nanomembrane transistors. This system enables high‐fidelity electrophysiological recordings from the moist, curved surface of a beating pig heart. The device samples simultaneously through 288 amplification and multiplexing channels, achieving an ultra‐high spatiotemporal resolution down to sub‐millimeter and sub‐millisecond levels.^[^ ^58^ ^]^ Around the same time, Kim et al. successfully demonstrated a novel “epidermal electronic system (EES),” which closely integrates with the skin surface using ultra‐thin, low‐modulus, lightweight, and stretchable electronic devices, enabling highly sensitive monitoring of physiological signals.^[^ ^59^ ^]^

The performance of these devices is largely dependent on their effective interface with biological tissues. For OECTs, due to the varying conformations and sizes of biomolecules, devices need to be meticulously designed for the intended application to enable sensitive and selective analysis of the target components in bodily fluids at low biomolecule concentrations. A representative case involves functionalizing the gate surface of OECTs with a PANI/Nafion–graphene composite film, achieving a hydrogen peroxide (H_2_O_2_) detection limit that surpasses those of common interfering species like dopamine and ascorbic acid by approximately three orders of magnitude.^[^ ^60^ ^]^ Similarly, ion‐selective membranes (ISM) containing ion carriers achieve ion carrier selectivity by reversibly interacting with target ions to form complexes, thereby providing ion selectivity.^[^ ^61^, ^62^, ^63^ ^]^

In the context of wearable and implantable devices, the primary objective of device fabrication is to establish a consistent and stable interface between the sensor and the tissue or organ, ensuring the bioelectronic device remains securely positioned over prolonged periods.^[^ ^57^ ^]^ This requirement is based on significant physiological factors; for example, during human movement, the multi‐axial motion of joints causes the skin on the forehead and around the eyes to stretch and compress considerably. The heart beats 60 to 100 times per minute, maintaining a total volume change of approximately 8% over the entire cardiac cycle, with the resulting periodic fluctuations in arterial blood pressure causing localized deformations (on the order of hundreds of micrometers) in the brain.^[^ ^64^ ^]^ Moreover, the interface between such distinctly different soft tissues (with Young's moduli ranging from 100 Pa to 10 MPa) and electronic materials (with Young's moduli ranging from 1 to 100 GPa) plays a critical role in the occurrence of foreign body reactions due to mechanical mismatch.^[^ ^57^, ^65^ ^]^ This necessitates that bioelectronic interfaces exhibit compliance, meaning the ability to closely adhere to the surface of tissues or organs even under dynamic and deforming conditions, thereby making bioelectronics less detectable by the host biology. This is a prerequisite for bioelectronics to ensure the high‐fidelity conversion of various physiological signals into electrical signals during complex movements within a biological system. Several approaches have been employed to engineer mechanical compliance, including the use of substrates with low Young's moduli,^[^ ^66^ ^]^ miniaturizing or even microminiaturizing devices to achieve smaller invasive areas,^[^ ^67^ ^]^ optimizing shape factors such as mesh or honeycomb grid structures,^[^ ^68^, ^69^ ^]^ and employing untethered interfaces for wireless data transmission.^[^ ^70^, ^71^ ^]^

For devices functioning in vivo, in addition to the mechanical compliance required for contact with delicate soft tissues, biocompatibility must also be considered to minimize immune rejection and cellular damage. The insertion and maintenance of these devices may demand an invasive procedure. Implanted materials may trigger a foreign body response (FBR), resulting in fibrotic encapsulation that compromises device performance and introduces potential risks related to eventual clearance or rejection.^[^ ^72^ ^]^ Common strategies include material selection and device optimization design. For example, materials such as parylene and polyimide can be used as substrates for OECTs, and OMIEC (e.g., PEDOT:PSS), along with these substrate materials, can undergo sterilization, meeting the sterility requirements for in vivo applications.^[^ ^73^ ^]^ Some synthetic biocompatible materials, such as those synthesized on PEtU‐PDMS‐based OECTs, show high cell viability (up to 89%) as evaluated by MTT assays, demonstrating good biocompatibility.^[^ ^74^ ^]^ Some biodegradable matrices,^[^ ^75^, ^76^ ^]^ such as Poly (Lactic‐Co‐Glycolic Acid) (PLGA), degrade into non‐toxic byproducts in vivo and do not induce inflammatory responses within 30 days post‐implantation, thereby eliminating the need for device removal.^[^ ^77^ ^]^ Furthermore, various fabrication methods based on OMIECs allow for the construction of vertically configured devices, with the OECT channel positioned between vertically stacked source and drain electrodes, helping to minimize the overall size of the implant.^[^ ^78^ ^]^

### 3.2 From Physical Interfaces to Molecular Recognition Interfaces

The technical progress of OECT in the design of physical interfaces has established a critical foundation for the stable integration of devices with biological systems. However, while optimizing physical interfaces is a key step in successfully integrating bioelectronic devices, achieving high sensitivity and specificity in biological signal detection requires further development in molecular recognition interface design. This transition is essential for enhancing the performance of OECTs in biosensor applications.

The working principle of OECT relies on the interaction between electrons and ions. In bioelectronic applications, the interface between OECT and biological tissues must exhibit excellent mechanical adaptability to ensure the stable transmission of signals. However, this optimization of the physical interface, although effectively promoting biological signal capture, remains limited to enhancing signal amplification and transmission capabilities. Building upon this physical foundation, the key challenge in current OECT applications is how to further enhance signal specificity and sensitivity by improving molecular selectivity. Through the design of molecular recognition interfaces, OECTs can transcend simple signal conduction and enable the detection of specific biomolecules, thus achieving efficient biosensing. The transition between physical and molecular recognition interfaces effectively merges traditional electrochemical sensor design with emerging biosensor technologies. The optimization of physical interfaces enables OECTs to maintain stable contact with biological tissues, while the design of molecular recognition interfaces allows OECTs to interact precisely with biomolecules, thus sensing specific molecules. To achieve this, the OECT interface must possess high chemical functionalization capabilities and be able to flexibly bind with various biological recognition molecules (e.g., antibodies, enzymes, aptamers, etc.).

Therefore, **Section** **4** provides a detailed discussion of the molecular recognition interface, serving as a natural extension of the physical interface's functionality. By modifying bioactive components (e.g., enzymes, antibodies, aptamers, or ion‐selective membranes) on key OECT components (e.g., the channel or gate), originally nonspecific physical contact can be upgraded to targeted bio‐electronic interactions. It is crucial to emphasize that this transition does not “replace” the physical interface but rather builds upon its stability. If OECTs detach from tissue during movement due to insufficient physical compliance or induce foreign‐body reactions due to poor biocompatibility, even the most precise molecular recognition interface will fail to function effectively. Conversely, OECTs with stable physical interfaces, but without molecular recognition capabilities, would fail to achieve precise diagnostics (e.g., early disease detection via biomarkers or real‐time monitoring of metabolic fluctuations).

## 4 Chemical‐OECT Interfaces

Having ensured a stable and biocompatible physical interface between the device and biological tissues, the research focus further shifts toward endowing these interfaces with specific bio‐recognition capabilities. Through carefully designed functionalization modifications of key device components (such as the channel or gate), the fundamental physical interface can be transformed into an intelligent sensing platform capable of detecting specific biomolecules with high selectivity and sensitivity.

**Figure 2:**
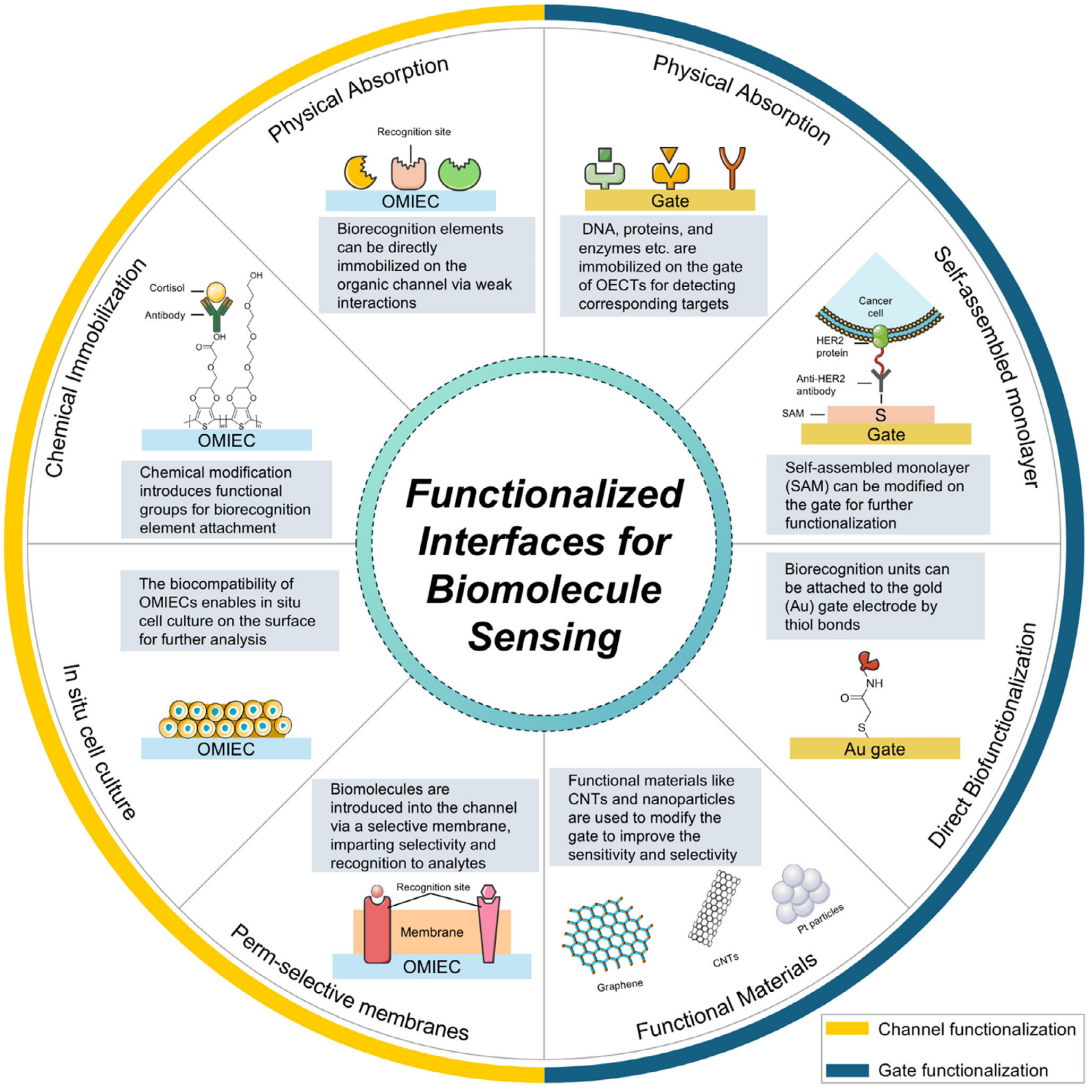
Methods for functionalizing the OECT channel and gate. Channel functionalization: Biorecognition elements can be immobilized on the channel via physical adsorption or through chemical modification strategies that introduce specific functional groups, enabling covalent bonding with antibodies. Additionally, cells can be cultured in situ on the OMIEC surface, or perm‐selective membranes can be used to selectively allow biomolecules of interest to enter the OMIEC. Gate functionalization: Biorecognition elements can be attached to the gate via physical absorption, or self‐assembled monolayers (SAMs) can be used to modify the surface for further functionalization. Direct biofunctionalization through thiol bonds can also be applied if the gate is made of gold (Au). Additionally, functional materials, such as graphene, CNTs, and Pt particles, can be used to modify the gate to enhance sensitivity and selectivity. Some components were adapted from Servier Medical Art templates, which are licensed under a Creative Commons Attribution 3.0 Unported License; https://smart.servier.com.

OECTs incorporating multiple biofunctional layers across distinct interfaces can act as selective recognition units for target analytes. This is typically accomplished by modifying the device with specific biomolecules, including antibodies, nanobodies, enzymes, or aptamers. The interface between the OMIEC and the electrolyte, as well as the gate and electrolyte, is often treated as a host for the recognition unit.^[^ ^15^ ^]^ Due to the rich chemical modifiability of OMIEC, various reaction sites and pathways are available for the functionalization of the channel. For example, functional side groups can be introduced into the main chain of the OMIEC via chemical synthesis, enabling covalent bonding with biorecognition elements.^[^ ^79^ ^]^ Moreover, the good biocompatibility of OMIEC materials enables cell culture on the channel surface, providing a natural platform for in situ cellular analysis.^[^ ^80^ ^]^ Due to the amplification properties of OECTs, functionalizing the gate–electrolyte interface is also a clear choice. The gate electrode is typically composed of metals or metal oxides, which can be functionalized through covalent or strong non‐covalent bonding with molecules containing linkers. Common types of channel and gate strategies are summarized in **Figure** **2**. These methods demonstrate practical versatility and compatibility with standard chemical functionalities, as they leverage naturally occurring chemical functionalities or groups that can be easily introduced through molecular engineering.^[^ ^81^ ^]^

### 4.1 Channel Functionalization

**Figure 3:**
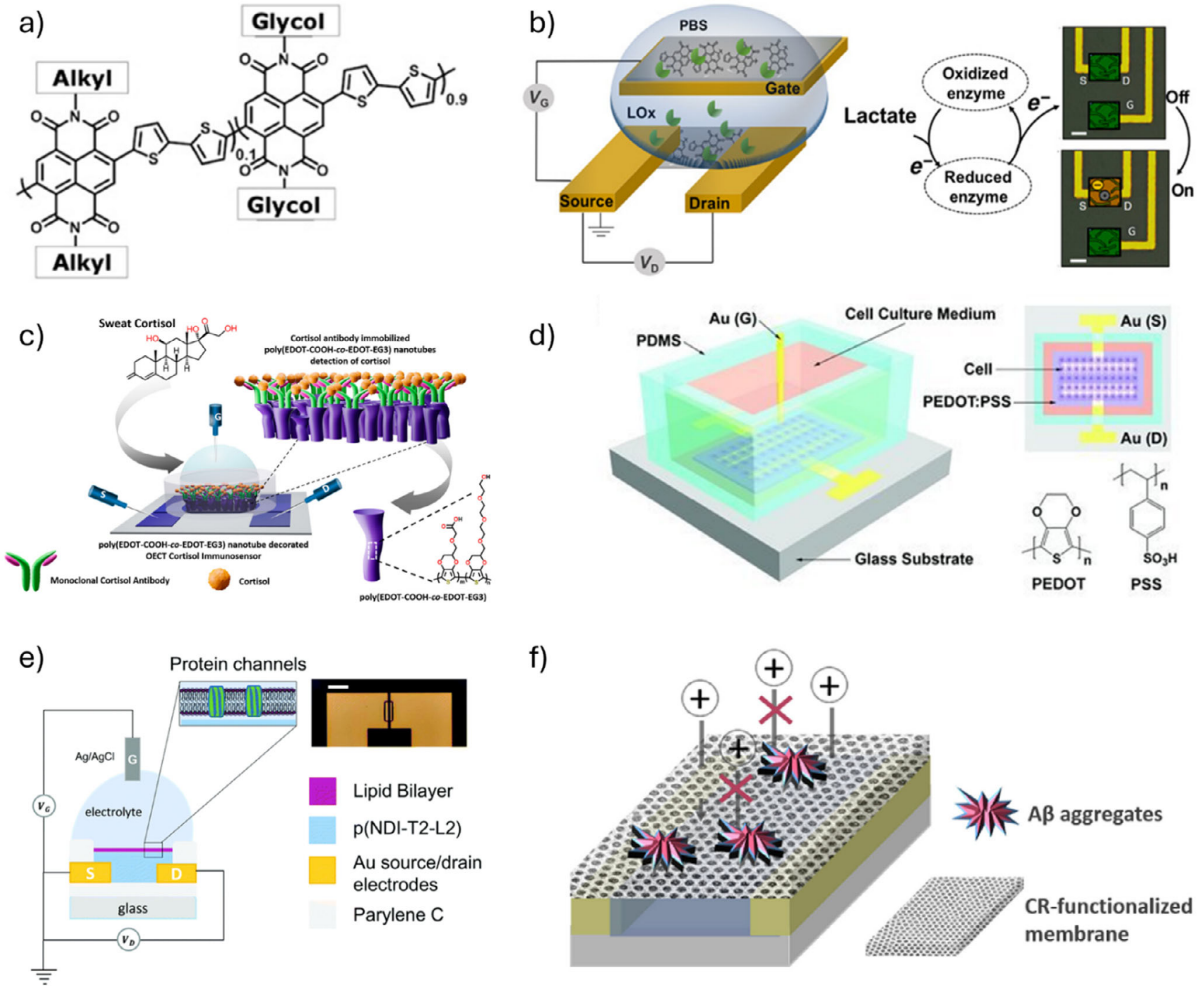
a) Molecular structure of the n‐type copolymer P‐90. b) Schematic illustration of an OECT device utilizing copolymer P‐90 in conjunction with lactate oxidase (LOx). a–b) Reproduced with permission.^[^ ^82^ ^]^ Copyright 2018, AAAS. c) Antibody immobilization on the nanotube‐functionalized OECT channel for selective cortisol detection. Reproduced with permission.^[^ ^79^ ^]^ Copyright 2022, American Chemical Society. d) Schematic of in situ cell culture on the OECT channel. Reproduced with permission.^[^ ^85^ ^]^ Copyright 2010, Wiley. e) Schematic diagram of OECT integrated with lipid layer. Reproduced with permission.^[^ ^88^ ^]^ Copyright 2022, The Royal Society of Chemistry. f) Schematic illustration of inhibited cation migration toward the PEDOT:PSS channel caused by the adsorption of A*β* aggregates on the membrane surface. Reproduced with permission.^[^ ^89^ ^]^ Copyright 2019, Elsevier.

The channel of OECT can be functionalized through simple physical adsorption or the physical immobilization of biomolecules. Despite the non‐covalent nature of physical adsorption, successful immobilization has been reported for various enzymes and antibodies under physiological conditions. For example, Pappa et al.^[^ ^82^ ^]^ used the n‐type material P‐90 as the active layer, with side chains containing polar ethylene glycol and nonpolar branched alkyl groups (**Figure** **3**a). The ethylene glycol side chains not only provide polar interaction sites for enzymes, allowing them to anchor effectively to the polymer film surface through interactions with the side chains, but also enhance the polymer's water absorption, thus improving electrochemical activity in aqueous media. This functionalization method eliminates the need for complex chemical treatments to immobilize enzymes or the addition of electron transfer mediators. Electrons produced through enzymatic reactions can be directly transferred to the conducting polymer backbone, allowing for the detection of target metabolites without the use of external redox mediators in lactate sensing (Figure 3b). This demonstrated versatility in enzymatic sensing applications; by replacing the enzyme used for channel functionalization with glucose oxidase (GOx), the same device structure can be utilized for glucose detection. It is worth noting that biomolecules immobilized through physical adsorption can better retain their conformation and activity. However, physisorption‐based immobilization has drawbacks, such as the desorption of bioreceptors from the surface during measurements and nonspecific adsorption of interfering molecules.^[^ ^83^ ^]^

Biomolecules can also be indirectly linked to OMIECs through the introduction of functional groups containing reactive sites that specifically interact with biomolecular functional groups. Due to the stability of covalent bonds between biomolecules and the OMIEC surface, bioreceptors remain firmly attached during measurements. This ensures the structural integrity of biomolecules during washing steps, fulfilling the stability requirements essential for analytical procedures.^[^ ^15^ ^]^ The functional groups necessary for covalent attachment can be introduced either by grafting onto the OMIEC surface or by depositing a sensing layer. For instance, our group utilized oxygen plasma treatment to generate hydroxyl groups on the PEDOT:PSS surface, followed by grafting a silane coupling agent. The terminal *primary amines (*–*NH*_2_) of the agent covalently bind to anti–E. coli O157:H7 antibodies, allowing the functionalized device to sensitively detect E. coli O157:H7 at concentrations as low as 10^3^ cfu mL^−1^.^[^ ^84^ ^]^ In the latter approach, a typical example is the work by Janardhanan et al.,^[^ ^79^ ^]^ who employed template‐free electrochemical polymerization to synthesize poly(EDOT–COOH–*co*–EDOT–EG3) nanotubes on the upper layer of PEDOT:PSS. The poly(EDOT–COOH) scaffold enables covalent immobilization of cortisol‐specific antibodies on the channel surface, whereas poly(EDOT‐EG3) effectively suppresses nonspecific adsorption of undesired cells and biomolecules (Figure 3c). Due to the stable chemical anchoring between cortisol antibodies and the channel, the OECT immunosensor retained nearly 93% of its initial drain current even after 20 days of storage. The process of chemical immobilization requires careful selection of functional groups on the OMIEC surface, taking into account the available functional groups and charge properties of biomolecules.^[^ ^83^ ^]^ To preserve the natural structure and activity of biological receptors, functionalization is typically performed under physiological conditions. For example, *primary amines (*–*NH*_2_) carry a positive charge under physiological conditions. On protein surfaces, these amine groups are typically outward‐facing, making them suitable for conjugation without causing biomolecular denaturation. Conversely, *carboxyl groups (*–*COOH)* carry a negative charge under physiological conditions and can be used to immobilize positively charged biomolecules. Alternatively, biomolecules can first be activated using EDC and sulfo–NHS, forming reactive intermediates that subsequently react with amine‐functionalized surfaces, resulting in conjugation.

The biocompatibility of OMIECs enables the in situ cell culture on the surface for further analysis.^[^ ^7^ ^]^ OECTs directly interfacing with cell cultures were shown to assess the integrity and health of barrier‐forming (non‐electrogenic) cells. For instance, our group pioneered the demonstration of in situ cell culture on the OECT channel (PEDOT:PSS) surface using human esophageal squamous epithelial cancqer cell lines (KYSE30) and fibroblast cell lines (HFF1) (Figure 3d), enabling detailed investigation of the relationship between cell detachqhment and metastatic properties.^[^ ^85^ ^]^ The sensing mechanism relies on the fact that cells cultured on the channel surface hinder ionic exchange between the electrolyte and the channel, thereby modulating the device's electrical response. Additionally, OECTs can function as single‐cell impedance sensors to quantitatively monitor impedance changes during cell adhesion and detachment processes. Their high sensitivity (current signal gain of 20.2 dB) enables the detection of subtle changes at the single‐cell level, providing a powerful tool for in‐depth understanding of cellular behavior and disease mechanisms.^[^ ^86^ ^]^ 3D cell culture, which allows cells to grow in all directions, can provide more comprehensive information compared to a 2D structure. By incorporating a 3D conducting polymer scaffold within the tubular structure enables simultaneous support for cell tissue growth and active signal transduction.^[^ ^87^ ^]^ The integrated fluid system offers greater ease of use and enhanced compatibility with biological systems. The tubular perfusion system enables continuous medium exchange, significantly accelerating cell adhesion and growth through in situ seeding. The device allows for continuous in situ monitoring of cell growth for over 44 hours until a confluent tissue layer is achieved.

Specific perm‐selective membranes can be employed to incorporate biomolecules into the channel region, utilizing either physical methods, such as molecular imprinting, or selective recognition mechanisms on membrane surfaces. For the former, for instance, cortisol can serve as a molecular template to initiate copolymerization with functional monomers and crosslinkers. After polymerization, the cortisol template is removed by washing, leaving binding sites complementary in size and shape to cortisol. These sites possess molecular memory, specifically recognizing and rebinding cortisol.^[^ ^62^ ^]^ For the latter, supported lipid bilayers (SLBs) integrated with transmembrane proteins have emerged as a promising method. For example, Kawan et al.,^[^ ^88^ ^]^ first demonstrated that SLBs could be formed on n‐type polymer‐based accumulation mode OECT channels by incorporating gramicidin A (GA) into DOPC bilayers (Figure 3e). The results showed selective permeability for monovalent cations, forming cation‐selective channels that permit passage of these ions. However, under certain conditions, the channel was impermeable to Ca^2+^, indicating that Ca^2+^ could block GA‐formed pores and thus decrease OECT current. Recently, droplet interface bilayer (DIB) technology has been utilized to fabricate transistor‐coupled droplet bilayers (TCDBs).^[^ ^90^ ^]^ TCDBs consist of lipid‐coated droplets immobilized onto a conductive polymer substrate, preventing direct interaction between the lipid bilayer and the PEDOT:PSS surface. The membrane resistance of TCDBs is approximately 1000‐fold higher than previously reported for PEDOT:PSS‐supported bilayers, effectively reducing nonspecific ion diffusion. Subsequently, alamethicin (Alm) peptides were introduced into the TCDB, forming cation‐selective channels. When a positive gate voltage is applied, the Alm channels allow cations to pass, thereby injecting them into the PEDOT:PSS channel and decreasing its conductivity. At low positive or negative voltages, Alm channels do not form, preventing ion transport and achieving selective ion permeability. Additionally, some perm‐selective membranes can simultaneously utilize both aforementioned selection mechanisms. For example, Congo red (CR) molecules can be immobilized on nanoporous membranes (approximately 50 nm pore size) covering the channel, enabling specific binding to amyloid‐*β* (A*β*), a biomarker closely associated with early Alzheimer's disease (AD) diagnosis (Figure 3f).^[^ ^89^ ^]^ Since A*β* is larger than the membrane pores, its capture by the membrane prevents electrolyte cations from diffusing into the channel. This mechanism enables modulation of the signal output based on the amount of A*β* captured from the electrolyte. Employing this approach, a low detection limit of 221 nM was achieved in human serum samples, demonstrating its potential for early AD diagnosis. Furthermore, integrating this system with microfluidic technology can significantly reduce both sample consumption and detection time, while preserving accuracy.^[^ ^91^ ^]^ Additionally, numerous reports highlight the employment of ion‐selective membranes (ISM) to modify the channel, enabling high selectivity and sensitivity for target ion detection, with further details provided in specialized reviews.^[^ ^92^ ^]^

### 4.2 Gate Functionalization

Utilizing the gate electrode serves as a more effective strategy for introducing bio‐functionality into the OECT in a controlled manner, compared to functionalizing the channel directly. This approach is demonstrated through several aspects: 1) Functionalization of an independent gate electrode does not directly affect channel conductivity, whereas direct modification of the conjugated polymer in the OECT channel can reduce carrier mobility; 2) Independent gate electrodes are typically constructed from conductive materials (e.g., Pt, Au, Ag/AgCl), which simplifies biosensor development due to the well‐established surface chemistry of these materials.^[^ ^15^ ^]^ 3) Gate electrode functionalization is typically achieved through straightforward methods, such as surface modification, coating, or deposition, allowing for versatile integration of catalysts, functional groups, or biomolecular recognition elements without contaminating the channel. In contrast, channel functionalization usually involves complex direct modifications of the conductive polymer, potentially altering its intrinsic electronic properties. Moreover, configuring the gate electrode as a functional interface enables multiplexing by allowing its placement on a separate substrate or physical decoupling from the channel. This configuration allows the use of multiple gate electrodes with a single channel, enabling simultaneous functionalization of several gates on the same device for the detection of multiple biological signals.

Physical adsorption is achieved by direct interaction between biomolecules and the solid surface (gate), following the same principle as channel modification. Physical adsorption is mainly governed by non‐covalent interactions, such as electrostatic forces, hydrogen bonding, and van der Waals interactions. This method offers operational simplicity, preserving biomolecular structure and activity. However, nonspecific adsorption can increase background noise, decreasing detection accuracy. Additionally, due to the relatively weak nature of non‐covalent interactions, adsorbed biomolecules may detach over time, affecting the long‐term stability of the device.^[^ ^93^ ^]^

**Figure 4:**
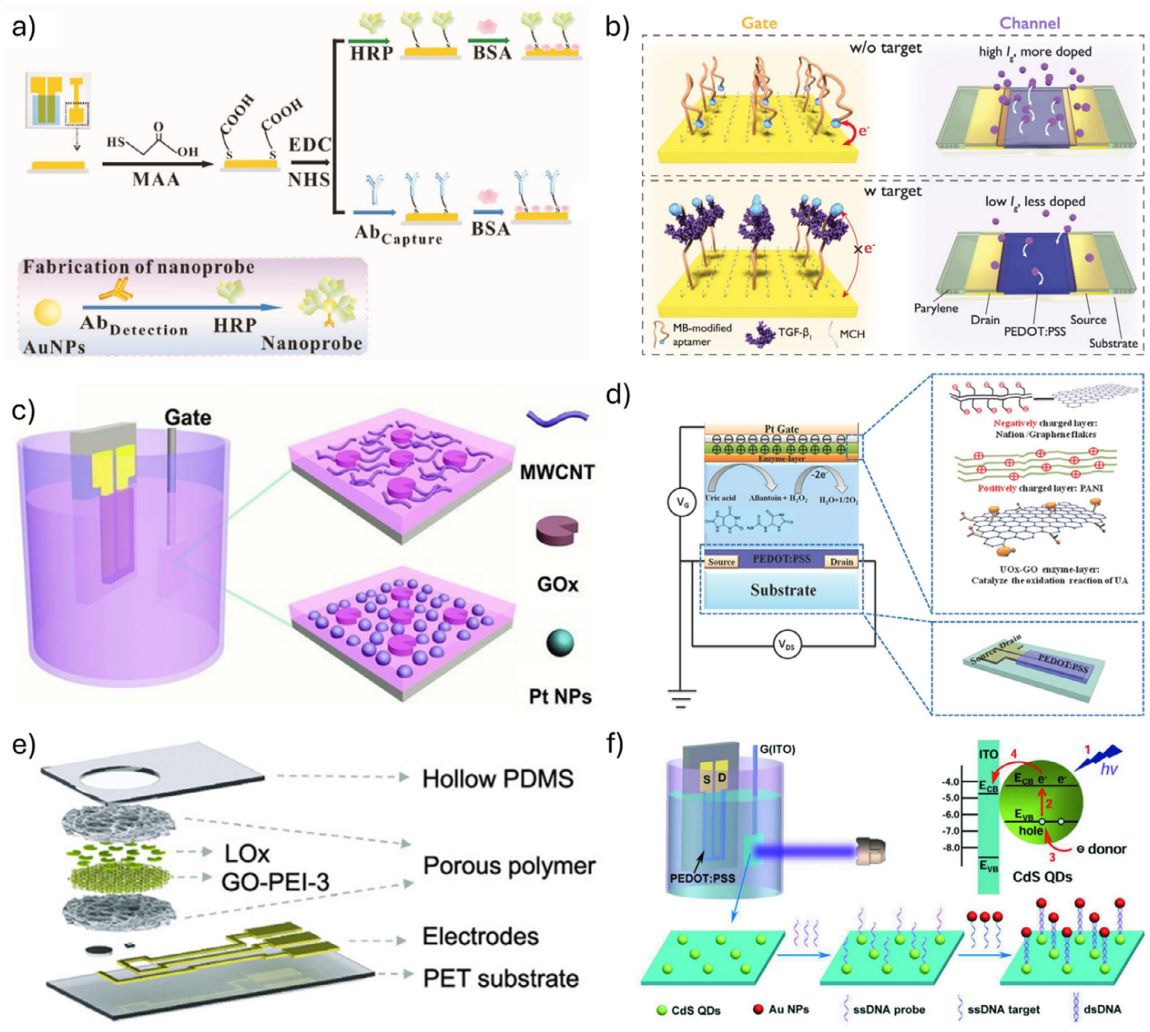
a) Detection of the cancer biomarker HER2 via gate functionalization of OECTs with catalytic nanoprobes. Reproduced with permission.^[^ ^94^ ^]^ Copyright 2017, Wiley. b) Detection of growth factor beta 1 (TGF‐β_1_) using an electrochemical aptamer‐based sensor. Reproduced with permission.^[^ ^96^ ^]^ Copyright 2023, Springer Nature. c) Highly sensitive glucose biosensors using MWCNT and Pt NPs modified gates.^[^ ^9^ ^]^ Copyright 2011, Wiley. d) Enhanced selectivity of enzyme biosensors in flexible OECTs by modifying Pt gate with PANI/Nafion–graphene bilayer. Reproduced with permission.^[^ ^99^ ^]^ Copyright 2015, Wiley. e) OECT chip incorporating a GO–PEI–3 membrane for enhanced sensitivity in lactate and uric acid detection. Reproduced with permission.^[^ ^66^ ^]^ Copyright 2024, Wiley. f) DNA sensing enabled by OPECT‐based platforms. Reproduced with permission.^[^ ^100^ ^]^ Copyright 2018, Wiley.

To enhance the density and stability of bio‐recognition elements, a self‐assembled monolayer (SAM) can be introduced. This covalent attachment method is typically realized through the assembly of gold–thiol binding, which is a common strategy for immobilizing carboxyl‐containing bio‐recognition elements onto Au surfaces. These immobilized units can subsequently be activated by the classical EDC/NHS coupling reaction (*N*‐(3‐(dimethylamino)propyl)*N'*‐ethyl‐carbodiimide hydrochloride/*N*‐hydroxysuccinimide), enabling covalent attachment of proteins.^[^ ^10^, ^94^ ^]^ When analytes bind to these protein‐based recognition elements, they induce changes in charge distribution, polarity, or interfacial capacitance at the gate electrode, consequently modulating the resulting channel current. For instance, our group demonstrated that mercaptoacetic acid (MAA)‐modified organic electrochemical transistor (OECT) gates exhibited ultrahigh sensitivity in detecting protein biomarkers of cancer, such as human epidermal growth factor receptor 2 (HER2) (**Figure** **4**a). The immobilization of HER2 antibodies onto the SAM‐functionalized Au gate facilitated highly efficient capture of HER2 proteins, resulting in significantly enhanced sensitivity compared to conventional electrochemical methods, with a detection limit as low as 10^−14^ g mL^−1^.^[^ ^94^ ^]^ Furthermore, we demonstrated the versatility of the MAA‐SAM strategy by covalently attaching SARS‐CoV‐2 spike protein antigen onto Au gates, enabling ultrafast (approximately 5 min) and highly sensitive detection of SARS‐CoV‐2 immunoglobulin G (IgG) antibodies at femtomolar concentrations in both saliva and serum samples.^[^ ^10^ ^]^ Similarly, 11‐mercaptoundecanoic acid (11‐MUA) can be used to immobilize SARS‐CoV‐2 antibodies on Au gates, achieving an exceptionally low detection limit (10^−17^ M) and outstanding device stability, which maintained high specificity and functionality even after prolonged storage (up to 20 days).^[^ ^95^ ^]^ Aptamers with thiol‐bearing terminals can directly bind to the Au gate without the need for SAM. For example, transforming growth factor beta 1 (TGF‐β_1_) can be detected by a methylene blue (MB) redox reporter modified on the Au gate surface. As shown in Figure 4b, in the absence of TGF‐β_1_, the aptamer conformation brings MB close to the electrode surface, resulting in a high working electrode current and more ion injection into PEDOT:PSS, leading to a large modulation of the channel current. In the presence of TGF‐β_1_, the aptamer conformation changes, moving MB away from the electrode surface, reducing the working electrode current, decreasing ion injection, and resulting in a smaller modulation of the channel current. The degree of modulation in the channel current correlates with TGF‐β_1_ concentration.^[^ ^96^ ^]^ Using the same method, the aminoglycoside aptamer [5′‐HO‐(CH_2_)_6_‐S‐S‐(CH_2_)_6_‐GGGACTTGGTTTAGGTAATGA‐GTCCC‐O‐CH_2_‐CHCH_2_OH(CH_2_)_4_‐NH‐CO‐(CH_2_)_2_‐methylene blue‐3′] was modified onto the gate to detect the antibiotic tobramycin. By applying a linear sweep square wave potential to the aptamer‐modified gate resulted in a transistor channel current response that was approximately two orders of magnitude higher than that of a comparable electrode‐based biosensor.^[^ ^97^ ^]^

Functionalization of the gate with materials such as nanostructures or specialized polymer membranes can markedly enhance the performance of OECTs.^[^ ^36^, ^98^ ^]^ Common approaches include modifying the gate with functional materials possessing high surface area and excellent electrical conductivity, which can facilitate electron/ion transfer, or using functional materials that selectively interact with specific analytes. These approaches, respectively, enhance interfacial reaction rates between the gate electrode and electrolyte, as well as improve detection selectivity. For example, our group fabricated OECT‐based glucose sensors (Figure 4c) by modifying Pt gates with either multi‐walled carbon nanotubes (MWCNTs) or platinum nanoparticles (Pt‐NPs) along with GOx.^[^ ^9^ ^]^ The introduced nanomaterials possess biomolecule immobilization capability and excellent electrocatalytic properties, which help increase the active surface area of electrodes, providing additional immobilization sites for GOx and thus enhancing enzyme loading capacity. The results indicated that the detection limit of the MWCNT‐CHIT/GOx/Pt electrode modified with MWCNT‐CHIT decreased to 0.5 µM, while that of the CHIT/GOx/Pt‐NPs/Pt electrode modified with Pt‐NPs further decreased to 5 nM, representing a three‐order‐of‐magnitude improvement compared to electrodes without nanomaterial modification. Furthermore, we demonstrated excellent selectivity toward H_2_O_2_ using an OECT with a gate electrode modified by a PANI/Nafion–graphene bilayer membrane (Figure 4d).^[^ ^99^ ^]^ The exceptional selectivity can be attributed to the unique structure and properties of the bilayer membrane, which effectively blocks interferents through electrostatic repulsion and molecular size exclusion. PANI, present as the positively charged protonated emeraldine salt form in phosphate‐buffered saline (PBS) solution, strongly repels positively charged molecules, such as dopamine (DA), through electrostatic interactions. In contrast, Nafion, possessing a stable Teflon backbone and negatively charged acidic sulfonate groups in PBS solution, effectively prevents the passage of anionic electroactive species, such as ascorbic acid (AA) and uric acid (UA). Additionally, large molecules such as glucose are hindered from passing through the nanochannels of the PANI and Nafion membranes. In contrast, H_2_O_2_ molecules, being the smallest among all analytes tested, can readily pass through the multilayer membrane and reach the Pt gate electrode. The modified OECT exhibited a detection limit for H_2_O_2_ as low as 3 × 10^−9^ M, which was three orders of magnitude lower than the detection limits for DA and AA. Similarly, positively charged polyethyleneimine (PEI) chains were intercalated into negatively charged graphene oxide (GO) sheets to prepare an adaptive GO membrane (GO‐PEI), which was subsequently modified onto the OECT gate electrode and immobilized with lactate oxidase (LOx) or urate oxidase (UOx) (Figure 4e).^[^ ^66^ ^]^ The PEI chains effectively suppressed swelling of the GO membrane and precisely regulated its interlayer spacing between 8.67 and 13.75 Å, thereby exhibiting size‐selectivity toward ions and biomolecules. Specifically, the membrane demonstrated significant selectivity toward larger biomolecules while exhibiting almost no obstruction to the transmission of H_2_O_2_. When the mass fraction of PEI was approximately 3%, the OECT achieved detection limits as low as 10^−9^ M for lactate and 10^−8^ M for uric acid. Photosensitive materials have been used to modify the gate of OECTs, leading to the development of organic photoelectrochemical transistor (OPECT)‐based DNA sensors.^[^ ^100^ ^]^ Exciton–plasmon interactions (EPIs) between CdS quantum dots (QDs) and gold nanoparticles (Au NPs) hinder charge transfer from the QDs to the Indium Tin Oxide (ITO) gate electrode, resulting in a reduced photovoltage and a corresponding modulation of the channel current (Figure 4f). This sensor demonstrated excellent selectivity for detecting DNA within a concentration range of 1×10^−15^ M to 1×10^−9^ M. An emerging direction in gate functionalization exploits the molecular specificity of biological receptors for multi‐analyte discrimination. For example, Song et al.^[^ ^101^ ^]^ proposed an artificial olfactory system that integrates human olfactory receptor (hOR)‐functionalized graphene extended gates with organic synaptic devices, enabling the detection and discrimination of volatile fatty acid mixtures based on conductance response patterns. This platform achieved over 90% recognition accuracy and demonstrates the promise of receptor‐based interfaces for bioinspired pattern recognition.

**Table** **2** summarizes the key examples of gate functionalization discussed in this section, categorized by analyte, channel material, sensing strategy, and the specific surface functionalization method used.

**Table 2:** Summary of representative gate functionalization approaches in OECT‐based sensing.

Analytes	Channel	Strategy	Detection limit	refs.
HER2	PEDOT:PSS	SAM	10^−14^ g mL^−1^	[94]
SARS‐CoV‐2 IgG	PEDOT:PSS	SAM	100 fM	[10]
SARS‐CoV‐2 IgG	PEDOT:PSS	SAM	5.7 × 10^−19^ M	[31]
TGF‐β1	PEDOT:PSS	direct biofunctionalization (Au‐thiol bonds)	∼ 1 ng mL^−1^	[96]
Glucose	PEDOT:PSS	functional materials (MWCNT/Pt‐NPs)	2.5 nM	[9]
DNA	PEDOT:PSS	functional materials (CdS QDs)	10^−15^ M	[100]
Uric Acid	PEDOT:PSS	functional materials (PANI/Nafion‐graphene)	10 nM	[99]
Cholesterol			0.1 µM	
Glucose			30 nM	
Lactate	PEDOT:PSS	functional materials (GO‐PEI membrane)	∼ 10 nM	[66]
Uric Acid			∼ 10 nM	

### 4.3 Roles of Orientation and Debye Length at Bio‐Electronic Interfaces

**Figure 5:**
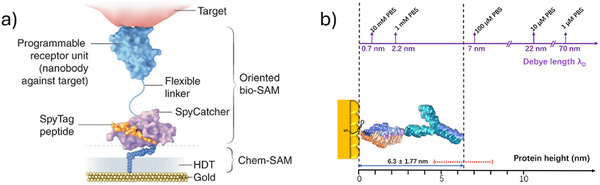
a) A chem‐SAM formed by SpyTag coupling to HDT enables covalent attachment of interchangeable nanobody–SpyCatcher fusion proteins (bio‐SAM). Reproduced with permission.^[^ ^31^ ^]^ Copyright 2021, Springer Nature. b) Comparison between the Debye lengths of PBS solutions and the characteristic height of proteins. Reproduced with permission.^[^ ^10^ ^]^ Copyright 2021, AAAS.

Ideal immobilization of antibodies or enzymes should possess at least two key characteristics: antibodies should be firmly bound to the substrate, and their immobilization should be highly controllable and site‐specific.^[^ ^102^ ^]^ Therefore, precise orientation can prevent false‐negative results and ensure lower detection limits. Although functionalization enables efficient immobilization of biomolecules on the channel or gate, the resulting functionalized interface may not represent the optimal sensing configuration. Misorientation of antibodies or enzymes may impede their accessibility to target analytes—especially those binding at the outermost interface—which can significantly reduce sensing sensitivity.^[^ ^15^ ^]^ Designing recombinant proteins and utilizing protein conjugation systems to achieve oriented immobilization of proteins on sensing interfaces is considered an effective strategy. For instance, Guo et al.^[^ ^31^ ^]^ engineered a recombinant fusion protein by combining an anti‐GFP nanobody with the SpyCatcher domain (**Figure** **5**a). Initially, a chemically self‐assembled monolayer (chem‐SAM), composed of 1,6‐hexanedithiol (HDT) and chemically modified SpyTag, was constructed on the Au gate electrode. Subsequently, the fusion protein was incubated with the chem‐SAM under physiological conditions. By utilizing the highly specific interaction between the SpyTag peptide and the SpyCatcher domain, along with their autocatalytic formation of a covalent isopeptide bond, the fusion protein was immobilized with well‐defined molecular orientation and configuration, leading to the formation of a biologically derived self‐assembled monolayer (bio‐SAM). This facilitated the oriented immobilization of proteins on the sensor surface, enabling the detection of the SARS‐CoV‐2 spike protein at femtomolar concentrations with a rapid response time.

Careful control of the solution's Debye length is critical for the unambiguous and selective detection of macromolecules.^[^ ^103^ ^]^ The Debye length refers to the effective distance over which electrostatic interactions are significant in an electrolyte solution. It is defined as the distance from an ion's center within which the surrounding ionic atmosphere—shaped by thermal motion and electrostatic forces—reaches equilibrium. Beyond this distance, the electrostatic influence of the ion becomes negligible. In essence, protein molecules situated beyond the Debye length from the gate exert a negligible influence on the gate potential. The Debye length in an electrolyte can be calculated using the following expression:^[^ ^104^ ^]^

(3) $$\lambda_{D}=\sqrt{\frac{\varepsilon kT}{2N_{A}q^{2}I}}$$

where *ε* is the absolute permittivity of the electrolyte, *k* is Boltzmann's constant, *T* is the temperature, *N*_A_ is Avogadro's number, *q* is the elementary charge, and *I* is the ionic strength of the solution. Although multiple factors influence the Debye length, its magnitude is primarily governed by the ionic strength. For instance, in high‐concentration PBS solutions, the Debye length becomes shorter than the average size of biomolecules. In 1× PBS (approximately 150 mM ionic strength), the Debye length is around 0.7 nm, which is significantly smaller than the typical height of an IgG molecule, approximately 10–15 nm. This disparity renders biomolecules such as IgG undetectable in high‐ionic‐strength environments due to insufficient electrostatic screening length for effective interaction.^[^ ^103^, ^105^ ^]^ A common strategy to address this limitation involves diluting the electrolyte solution. By carefully adjusting the ionic concentration, it is possible to lower the detection limit of an IgG sensor by up to two orders of magnitude (as low as 100 fM) (Figure 5b).^[^ ^10^ ^]^ However, this approach is not universally effective. Certain biomolecules require specific ionic strengths to maintain their native conformations, and in some cases—such as in situ monitoring in physiological fluids—the ionic concentration must remain unchanged, resulting in a Debye length of less than 1 nm.^[^ ^106^ ^]^

## 5 Skin–OECT Interfaces

The successful integration of OECTs with human skin depends on the establishment of a mechanically compliant, biocompatible, and electrically stable interface. In contrast to conventional rigid electronics, OECTs must accommodate the dynamic, hydrated, and microscopically irregular properties of the epidermis in order to maintain consistent signal transduction. In a recent review, Kim and colleagues provided an insightful summary of material design and integration strategies for soft bioelectronics in digital healthcare.^[^ ^107^ ^]^ This section discusses the diverse strategies used to engineer effective skin–OECT interfaces, including material innovation, surface engineering, and architectural refinement. By addressing both structural and interfacial design challenges, these efforts support stable and high‐resolution bioelectronic monitoring in practical, real‐world settings.

### 5.1 Implementation of Seamless Interface

In skin‐mounted OECTs, sensing is realized through direct contact between the channel and the tissue surface. The efficiency of biosignal transduction is primarily determined by the nanoscale proximity between the semiconductor channel and the biological interface. Therefore, establishing a seamless interface between the channel and the skin is essential.^[^ ^15^ ^]^ Overcoming the challenges of tissue‐device integration requires the careful consideration of material properties, structural designs, and interface modifications.

#### 5.1.1 Ultrathin and Substrate‐Free Architectures

**Figure 6:**
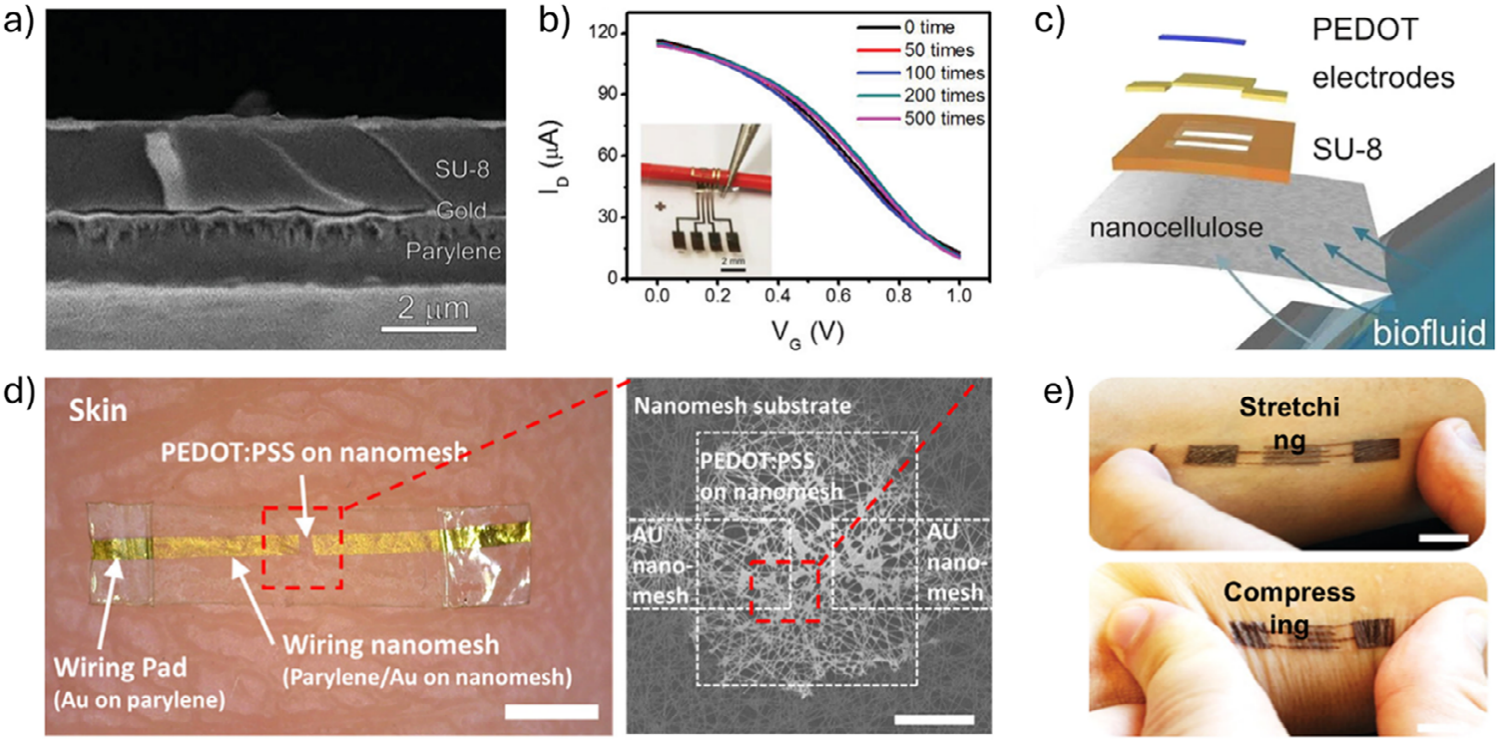
a) Sandwich structure of an ultra‐thin OECT. b) Transfer characteristics of an ultra‐thin OECT after bending for different times. Reproduced with permission.^[^ ^110^ ^]^ Copyright 2023, Wiley. c) Schematic illustration of a single OECT decal comprising a nanocellulose substrate, inkjet‐printed SU‐8 layer, evaporated gold electrodes, and a drop‐cast PEDOT:PSS channel. Reproduced with permission.^[^ ^111^ ^]^ Copyright 2017, Springer Nature. d) Photograph of a nanomesh OECT (NMOECT) adhered to the skin (left, scale bar: 1 mm) and SEM image showing the channel region of the device (right, scale bar: 200 µm). Reproduced with permission.^[^ ^112^ ^]^ Copyright 2020, American Chemical Society. e) Optical images of skin‐conformal OECTs subjected to mechanical deformation (scale bar: 6 mm). Reproduced with permission.^[^ ^113^ ^]^ Copyright 2019, Wiley.

The degree of conformability dictates how effectively a thin film adheres to uneven or textured surfaces. Enhancing film‐to‐substrate conformability improves the effective adhesion strength. Studies have shown that the ratio of film thickness to the characteristic wavelength of the substrate's surface roughness significantly affects conformability.^[^ ^108^ ^]^ For normal skin, the root mean square (RMS) roughness ranges from 0.03 to 45 µm; thus, thinner films are more likely to establish a fully conformed (FC) interface with the skin surface.^[^ ^109^ ^]^ Following this principle, our group employed an ultra‐thin parylene substrate (thickness < 2 µm) to fabricate an OECT array for high‐resolution human electrocardiogram (ECG) mapping (**Figure** **6**a).^[^ ^110^ ^]^ The resulting devices exhibit a high on‐off ratio (> 100), high transconductance (0.26 mS), and fast response time (down to 10 µs). Furthermore, the devices show minimal performance degradation after 500 cycles of bending, demonstrating excellent mechanical durability under repetitive deformation (Figure 6b). Certain novel substrate materials enable both conformability and efficient fluid transport from the bottom to the top sensor interface. For example, a bioelectronic decal fabricated using a thin (< 20 µm) porous microbial nanocellulose membrane as the substrate achieves a total thickness of merely 25 µm.^[^ ^111^ ^]^ The use of ultra‐thin microbial nanocellulose provides flexibility, mechanical robustness, hydrophilicity, and permeability to both liquids and gases (Figure 6c). When simulated sweat is delivered to the sensor element via vertical wicking, the microbial nanocellulose‐based OECT, mounted on a cotton pad, reaches its minimum current within 30 s. The detection limit for NaCl concentration is as low as 17.1 µM, while maintaining a broad dynamic range. Testing on chicken skin demonstrated that the device's performance is comparable to other test surfaces, indicating its suitability for application on irregular, contoured biological tissues. Similarly, Someya et al. used electrospinning to fabricate a porous nanomesh substrate composed of randomly distributed polyurethane (PU) nanofibers, as shown in Figure 6d.^[^ ^112^ ^]^ They then coated the surface of the PU nanomesh with a 250 nm‐thick layer of parylene to reinforce junctions between PU nanofibers while maintaining the porous nanomesh architecture. This structure functioned as the substrate for the OECT device, with a total thickness of only 1.5–2 µm. The ultra‐thin configuration endowed the device with high flexibility, facilitating a pre‐strained configuration that accommodates skin deformation. Even without intrinsic adhesion, the device maintained stable contact and attachment to the skin surface.

Using skin as a substrate for the device can eliminate the non‐conformal contact between traditional substrates and the skin. Achieving this requires overcoming the challenge of transferring the active layer (e.g., PEDOT:PSS) onto skin. Zhang et al.^[^ ^113^ ^]^ initially investigated the effects of different surfactants, additives, and annealing conditions on the transfer performance of PEDOT:PSS, discovering that 4‐Dodecylbenzenesulfonic acid (DBSA) facilitated the transfer process. They then used a hydrogel carrier to detach the PEDOT:PSS film from the glass substrate. Subsequently, they employed laser ablation with a shadow mask technique to deposit and pattern source‐drain electrodes onto the tattoo paper. Finally, the PEDOT:PSS film, supported by the hydrogel, was carefully transferred onto the tattoo paper, and the OECT component was directly transferred to the skin using a water‐soluble tattoo paper, creating a highly consistent tattoo‐like electronic interface. Due to the low thickness of the device, van der Waals forces promote seamless contact with the skin, maintaining contact and stable signal output during mechanical deformations of the skin, such as stretching and compression (Figure 6e).

#### 5.1.2 Interfacial Engineering

**Figure 7:**
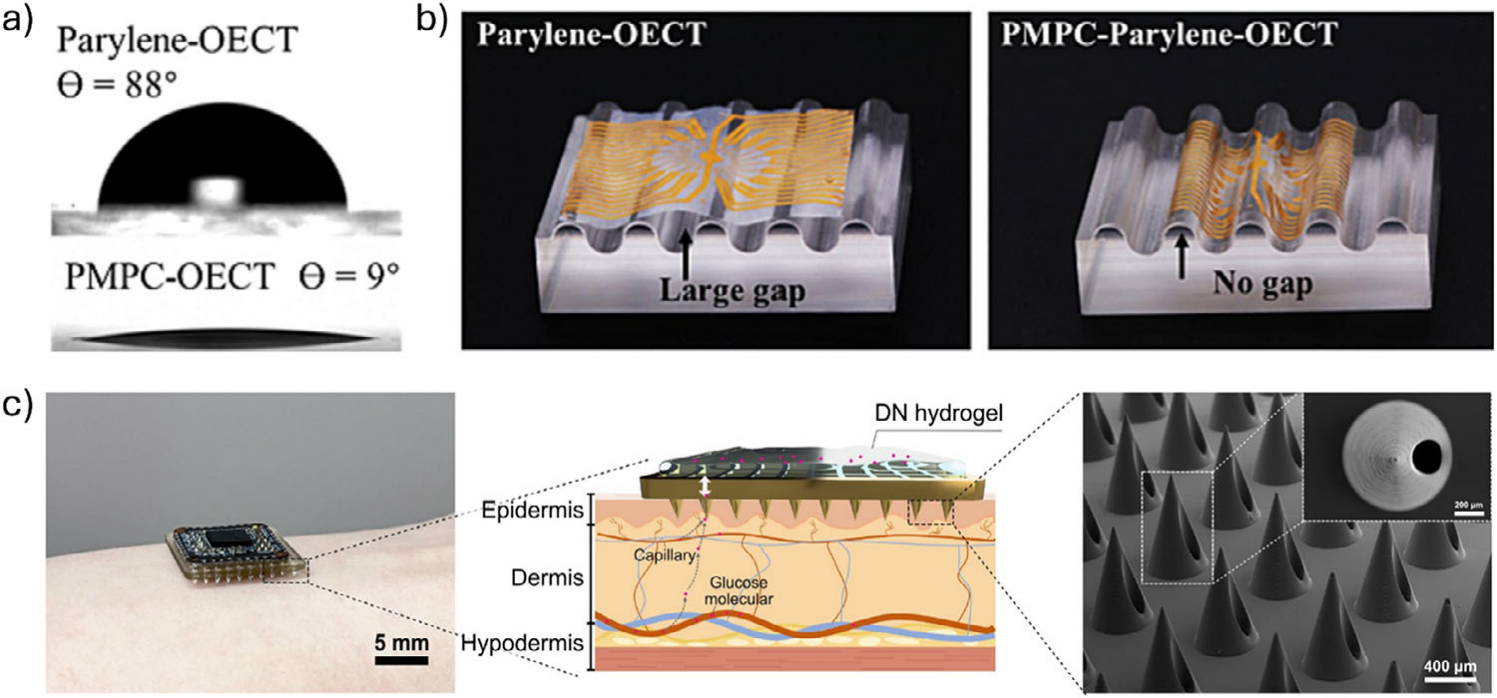
a) Contact angles of Parylene OECT(Top) and PMPC OECT(Bottom). b) Demonstration of conformability by wrapping wet Parylene‐encapsulated (left) and PMPC–Parylene‐encapsulated (right) OECT arrays onto circular PDMS substrates with grooved surfaces (radius = 1.5 mm). Reproduced with permission.^[^ ^116^ ^]^ Copyright 2024, Elsevier. c) Optical image of the OECT‐based continuous glucose monitor (OECT‐CGM) applied to the skin (left); schematic illustration of glucose diffusion from interstitial fluid (ISF) to the OECT through the microneedle interface (middle); SEM image of the microneedle structure (right). Reproduced with permission.^[^ ^71^ ^]^ Copyright 2024, AAAS.

Interfacial engineering plays a crucial role in enhancing the adhesion and functionality of OECTs when interfacing with the skin. Robust adhesion to the skin can be achieved by incorporating a combination of physical and chemical interactions at the device–tissue interface. These mechanisms may involve hydrogen bonds, hydrophobic forces, metal–ligand coordination, π–π stacking, cation–π interactions, and covalent linkages.^[^ ^114^, ^115^ ^]^ By modifying the surface properties of the device, such as increasing hydrophilicity or introducing functional groups, the interaction between the OECT and the skin can be significantly improved. For example, Zhang et al. proposed a method for modifying the interface of bioelectronic devices using hydrated zwitterionic materials to enhance hydrophilicity and adhesion to the skin.^[^ ^116^ ^]^ By grafting poly(2‐methacryloyloxyethyl phosphorylcholine) (PMPC) onto the encapsulation layer through atom transfer radical polymerization (ATRP), the device surface was modified to exhibit high hydrophilicity (**Figure** **7**a). This modification significantly improves the wetting properties and adhesion to biological surfaces, such as skin, through capillary forces driven by hydrated zwitterions. Additionally, the modification softens the device surface, reducing its elastic modulus to approximately 35 kPa, which is more than three orders of magnitude lower than the 450 MPa modulus of the unmodified parylene surface. This reduction in modulus enhances the flexibility and biocompatibility of the device, making it more compatible with soft biological surfaces like skin (Figure 7b). Building on this foundation, the research team advanced a zwitterion‐compatible hermetic encapsulation strategy and refined the layer‐by‐layer deposition technique. This approach is anticipated to impart the layered parylene system with ultralow biofouling characteristics while maintaining its mechanical integrity and encapsulation performance.^[^ ^117^ ^]^

In addition to chemical modifications, physical methods also play a crucial role in enhancing the adhesion between the device and skin, with the introduction of microneedle arrays being one such effective solution. Microneedles, typically ranging from 50 to 1000 micrometers in length, create micro‐scale punctures in the stratum corneum, facilitating direct interaction between the skin and the device while minimizing pain or discomfort.^[^ ^11^ ^]^ By leveraging a “skin anchoring” effect, microneedles enhance the stability of the sensor‐dermis interface, significantly improving adhesion during motion. As reported by Bai et al.,^[^ ^71^ ^]^ the use of microneedle arrays has shown promising results in continuous glucose monitoring systems (CGMs), enabling minimally invasive sampling of interstitial fluid (ISF). The microneedle patch, combined with a soft adhesive hydrogel, improves the device's skin compatibility and helps maintain a stable skin‐device interface during motion, facilitating glucose diffusion from the ISF to the OECT sensor.(Figure 7c). Additionally, the incorporation of microneedles contributes significantly to the long‐term durability of CGMs. By reducing skin irritation and maintaining reliable adhesion, these devices can function for extended periods without discomfort. This strategy not only ensures stable sensor operation during motion but also enhances overall wearability and durability, supporting its application in continuous glucose monitoring systems.

#### 5.1.3 Gel Interlayers

**Figure 8:**
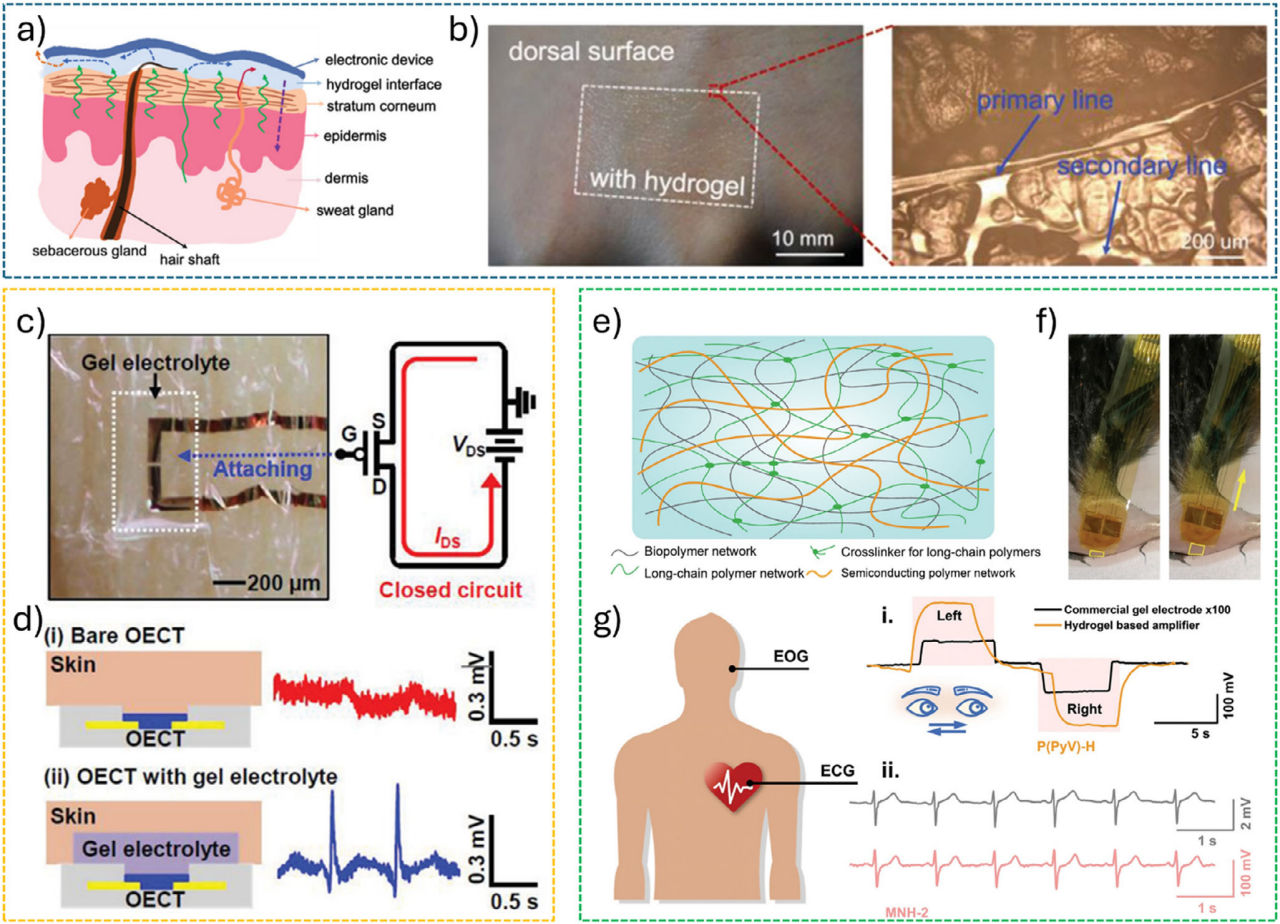
a) Schematic of the bioelectronics/hydrogel/skin hybrid interface. b) Schematic illustration of an ultrathin hydrogel film applied to the back of the hand, covering the extensor digitorum tendons (left); primary and secondary skin lines remain visible beneath the hydrogel layer (right). Reproduced with permission.^[^ ^121^ ^]^ Copyright 2023, Wiley. c) Optical image (left) of the wearable OECT‐based sensor on the skin and schematic of its circuit layout (right) d) Comparison of the OECT structure schematics with (i) and without (ii) solid electrolytes, and their corresponding electrocardiogram (ECG) signals recorded from the skin. Reproduced with permission.^[^ ^122^ ^]^ Copyright 2019, Wiley. e) Schematic illustration of the microstructures of the multiple network hydrogels (MNH). f) Photographs of OECT devices based on MNH‐2 adhered to skin tissue, demonstrating stable attachment before and during repeated tensile deformation. g) Comparative analysis of electrophysiological signals: (i) electrooculography (EOG) and (ii) electrocardiography (ECG) recorded using hydrogel‐based amplifiers versus commercial gel electrodes. Reproduced with permission.^[^ ^123^ ^]^ Copyright 2024, Wiley.

Introducing a soft intermediate layer between the skin and OECTs is a promising strategy to bridge the soft, dynamic, and hydrated nature of skin with various thin‐film‐based electronics (**Figure** **8**a). Gels, consisting of cross‐linked three‐dimensional polymer networks, possess mechanical characteristics comparable to those of biological tissues, with elastic moduli spanning from 1 Pa to 300 MPa, thereby encompassing the typical modulus range of skin and muscle (200–500 kPa). More importantly, their high‐water content facilitates efficient ion transport, thereby enhancing compatibility with biological environments.^[^ ^118^ ^]^ Owing to their excellent tunability, design flexibility, and favorable mechanical properties, gels can mimic natural tissues, reduce interfacial resistance, and improve conformability with biological systems.^[^ ^119^, ^120^ ^]^ For example, Cheng et al.^[^ ^121^ ^]^ proposed a technique for the preparation of ultrathin hydrogel interfaces via cold lamination, successfully fabricating a uniform hydrogel film with a thickness as low as 7 µm and an area exceeding 100 cm^2^ by controlling the crosslinking density of the polyacrylamide‐alginate (PAAM‐alginate) interpenetrating network. The Young's modulus of the hydrogel (34.3 – 540.2 kPa) closely matches that of human skin (≈ 130 kPa), allowing for micron‐scale deformation that enables it to embed into the extensor tendons of the hand and exhibit clear texture (Figure 8b). Furthermore, the mechanical interlocking and chemical adhesion synergy between the hydrogel and the skin helps withstand shear forces generated by skin movement, with no significant loss in adhesion performance after 50 repeated applications. When the OECT array (8 channels, channel dimensions 100 × 300 µm) is attached to the back of the hand via the hydrogel, its transconductance (*g*_m_) uniformity reaches 95%, and during continuous monitoring of epidermal electrical signals (e.g., electromyography (EMG)), the noise level is below 5 mV, representing a 30% reduction compared to traditional adhesive tape fixation methods. This is attributed to the close contact between the hydrogel and the skin, which reduces interfacial impedance fluctuations. Furthermore, the gel can serve as an ionic conductor bridge, establishing a conductive pathway between the skin and the channel. For instance, Lee et al. designed an ultrathin, non‐volatile gel electrolyte, using a glycerol‐based gel (≈ 6 µm thick) as the electrolyte, crosslinked from polyvinyl alcohol (PVA) and acrylamide, and containing 0.7 M NaCl inside the gel.^[^ ^122^ ^]^ The OECT, combined with this gel layer, forms a soft and conformal interface with the skin.(Figure 8c). The gel acts as an ionic conductor, forming an ionic pathway between the skin and the channel. The skin potential is transmitted through the gel as a gate voltage to modulate the operation of OECTs. However, OECTs without gel electrolytes lack a medium for ionic conduction, thereby preventing effective electrical coupling between the skin and the device and hindering ECG signal detection. In contrast, when an OECT covered with a glycerol gel electrolyte comes into direct contact with the skin, the NaCl ionic solution within the gel acts as a conductive medium, establishing an ionic pathway between the skin potential and the device's gate, enabling stable ECG signal recording (Figure 8d).

Traditional gels serve only as ionic conductors or insulating matrices, whereas through rational molecular design, gels can integrate both electronic and ionic conduction functionalities, coupled with adhesion and mechanical flexibility. For example, Li et al.^[^ ^123^ ^]^ developed a multi‐network hydrogel (MNH), combining a semiconductor polymer network with a bioadhesive network (e.g., gelatin, PAA), achieving synergistic optimization of mechanical flexibility, bioadhesiveness, and semiconductor properties (Figure 8e). First, they synthesized poly(pyridine vinylene) (P(PyV)) with a cationic backbone and used chloride ions as counter‐ions. Through electrostatic crosslinking with disodium 1,3‐benzenedisulfonate (DBS), they formed a three‐dimensional porous single‐network hydrogel (P(PyV)‐H). The structure undergoes ion exchange, replacing chloride ions with the divalent anion DBS, thereby constructing a stable conductive network. During electrochemical reduction, anion de‐intercalation promotes electron delocalization along the main chain, imparting the hydrogel with semiconductor characteristics, such as an electron mobility of 0.25 cm^2^ V^−1^·s and an on/off ratio > 10^7^. They further formed a multi‐network system (e.g., MNH‐2) by incorporating biopolymers such as gelatin and polyacrylic acid, maintaining a µC* value of 9.72 F·cm^−1^·V^−1^·s^−1^ while enhancing the interfacial shear strength to 25 kPa and achieving a tensile strain exceeding 100%. The integrated design of adhesion, mechanical flexibility (Figure 8f), and ionic conduction addresses the issue of rigid mismatch between traditional electronic devices and the skin interface, as well as signal attenuation, particularly in the collection and amplification of bioelectrical signals. Thanks to the close contact between the hydrogel and the skin, the interfacial resistance for signal transmission is greatly reduced. Hydrogel amplifiers can capture weak electrooculogram (EOG) and electrocardiogram (ECG) signals and amplify signals in very low‐frequency ranges (e.g., 1 Hz). Additionally, the hydrogel provides a stable conductive path, reducing noise interference caused by traditional metal electrodes. For example, when using hydrogel amplifiers, the amplitude of the EOG signal can exceed 200 mV, while traditional gel electrodes produce signals with a much lower amplitude (Figure 8g).

### 5.2 Stretchable and Flexible Devices

Stretchability describes a material's capacity to undergo dimensional deformation under applied mechanical stress without structural failure.^[^ ^124^ ^]^ Owing to this property, stretchable electronic systems can conform to soft, elastic, and curved surfaces, enabling seamless integration with the human body—an advantage that often surpasses the limitations of conventional rigid electronics.^[^ ^7^ ^]^ Fulfilling this requirement relies on device optimization strategies, which encompass both material choice and structural or component‐level engineering. The synthesis of most OECT devices relies on a layer‐by‐layer manufacturing process, where the OECT is composed of a stack of multiple materials (e.g., substrate, electrodes, channel, electrolyte, packaging layer, etc.).^[^ ^10^, ^94^, ^110^ ^]^ Each material in the different layers may have a distinct Young's modulus, which can significantly impact interlayer stresses, adhesion parameters, and delamination behavior. **Table** **3** presents some important physical parameters of typical substrates, channels, electrodes, and electrolytes in stretchable OECTs. As the substrate and encapsulation layers are generally thicker and laminated, they predominantly influence the overall deformation experienced by the active materials or electrodes. Consequently, one of the most effective approaches to alleviating interfacial stress is to lower the elastic modulus of these encapsulating components.^[^ ^23^ ^]^ Moreover, appropriate structural design can enhance the deformability of the device, breaking the material engineering limits. This includes using structures with adjustable shapes (e.g., corrugated, curved, wrinkled, microcracks, and mesh patterns) and optimizing the layout when interconnecting the OECT with flexible conductors (e.g., Island–Bridge Layout, Buckled Structure). These methods will be discussed in detail in this section.

**Table 3:** The physical parameters of typical substrates, channels, electrodes, and electrolytes in stretchable OECTs.

	Materials	Density [g cm^−3^]	Young's modulus [GPa]	Crack‐onset strain [%]
Substrates	Polyimide (PI)	1.38–1.43	2.5^[^ ^125^ ^]^	≈ 3^[^ ^125^, ^126^ ^]^
	Poly(ethylene terephthalate) (PET)	1.38–1.45	2.8 ± 0.3^[^ ^127^ ^]^	≈ 12^[^ ^127^ ^]^
	Poly(ethylene‐2,6‐naphthalate) (PEN)	1.36–1.41	3.3 ± 0.4^[^ ^128^ ^]^	≈ 7^[^ ^127^ ^]^
	Parylene	1.29–1.36	2.8^[^ ^129^, ^130^ ^]^	24.7^[^ ^129^ ^]^
	Poly(dimethylsiloxane) (PDMS)	1.05	(0.4 – 3.5) × 10^−3[^ ^131^ ^]^	≈ 93^[^ ^132^ ^]^
	Styrene–ethylene–butylene–styrene (SEBS)	0.90–1.01	0.0254 ± 0.002^[^ ^133^ ^]^	518 ± 20^[^ ^133^ ^]^
Channel	PEDOT:PSS	1.01	1.7^[^ ^134^ ^]^	9^[^ ^134^ ^]^
	P3HT	/	0.252 ± 0.057^[^ ^135^ ^]^	> 150^[^ ^135^ ^]^
	PANI	1.2–1.3	2.6 ± 0.4^[^ ^136^ ^]^	6 ± 1^[^ ^136^ ^]^
	phs‐h‐DPP‐g2T	/	/	200
	PEDOT:PSS/PAAMPSA/IL	/	8 × 10^−4[^ ^137^ ^]^	800
	p(g2T‐T)	/	0.2–0.4^[^ ^138^ ^]^	200
Electrodes	Au	19.3	70	≈ 1^[^ ^139^ ^]^
	Pt	21.54	168	0.15
	Ag	10.49	83	0.07
	PEDOT:PSS	1.01	1.7^[^ ^134^ ^]^	9^[^ ^134^ ^]^
Electrolyte	Ionic liquid gel	1.0–1.5^[^ ^140^ ^]^	(60–120) × 10^−6[^ ^141^ ^]^	≥ 1500^[^ ^142^ ^]^
	Hydrogel	1.0–1.2^[^ ^143^ ^]^	39 × 10^−6^–50 × 10^−3[^ ^144^ ^]^	> 1000^[^ ^145^ ^]^

#### 5.2.1 Material Engineering

##### Flexible Substrate

**Figure 9:**
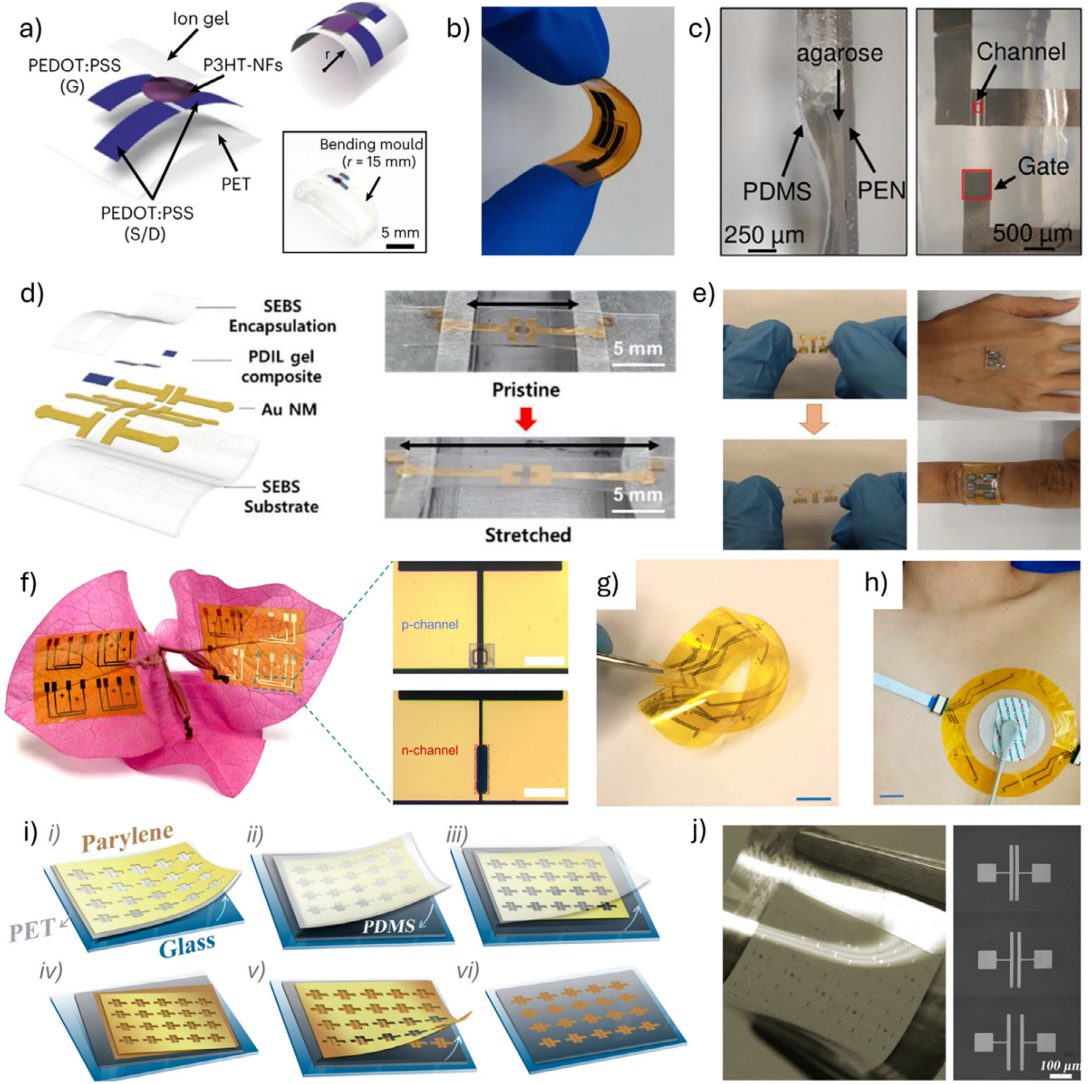
Schematic illustrations of OECT devices based on various substrates: a) PET, b) PI, c) PEN, d) SEBS, and e) PDMS. a) Reproduced with permission.^[^ ^147^ ^]^ Copyright 2023, Springer Nature. b) Reproduced with permission.^[^ ^146^ ^]^ Copyright 2023, The Royal Society of Chemistry. c) Reproduced with permission.^[^ ^148^ ^]^ Copyright 2025, Wiley. d) Reproduced with permission.^[^ ^154^ ^]^ Copyright 2022, MDPI. e) Reproduced with permission.^[^ ^155^ ^]^ Copyright 2019, Wiley. f) Photograph of an ultrathin flexible inverter placed on a petal (left) and channel microscopic image of the inverter (right); g) Photograph of an ultraflexible MOFECTs array; h) Photograph of an ultraflexible MOFECT array attached to the human chest. Reproduced with permission.^[^ ^153^ ^]^ Copyright 2023, AAAS. i) Parylene transfer process used to fabricate microelectrode arrays on PDMS. j) PDMS patterned with Au electrodes with substrate dimension of 15 mm × 15 mm. i,j) Reproduced with permission.^[^ ^156^ ^]^ Copyright 2019, American Chemical Society.

Using substrates with excellent flexibility, a Young's modulus similar to that of human skin, high biocompatibility, and chemical inertness is the most common strategy for realizing stretchable electronics. In recent years, various flexible substrates and their derivatives have been widely applied in the fabrication of flexible OECTs (**Figure** **9**a–e), including Polyamide (PI),^[^ ^146^ ^]^ Poly(ethylene terephthalate) (PET),^[^ ^147^ ^]^ Poly(ethylene‐2,6‐naphthalate) (PEN),^[^ ^148^ ^]^ Parylene, Styrene–ethylene–butylene–styrene (SEBS), and Poly(dimethylsiloxane) (PDMS). Among them, PET has garnered significant attention due to its excellent electrical insulation properties and its ability to maintain stability under severe temperature fluctuations.^[^ ^147^ ^]^ However, both PET and PEN are incompatible with high‐temperature (> 150 °C) manufacturing processes,^[^ ^149^, ^150^ ^]^ while device packaging processes (such as the post‐bake temperature of SU‐8, which can reach up to 160 °C) and annealing of certain channel materials may require such processes. In contrast, PI is more stable at high temperatures; however, its low crack‐onset strain (3%) limits its application in some extreme stretchable scenarios. Similarly, Parylene can also withstand high temperatures and can be fabricated into ultra‐thin flexible films through chemical vapor deposition (CVD).^[^ ^151^ ^]^ Additionally, Parylene possesses biocompatibility and biological stability, which are crucial for applications in biological interfaces.^[^ ^152^ ^]^

Recent studies have identified the use of ultrathin polyimide (PI) substrates as a key strategy for fabricating ultraflexible OECTs. Our research group has successfully demonstrated the integration of two‐dimensional metal–organic framework (MOF)‐based OECT arrays onto ultrathin PI substrates, allowing for their conformal attachment to the human body for wearable electrocardiogram (ECG) monitoring (Figure 9g,h).^[^ ^153^ ^]^ The thermal stability of the PI substrate supports crucial device processing steps, such as the annealing of two‐dimensional conjugated MOF (2D c‐MOF) films and SU‐8 patterning. Additionally, this study introduces an innovative metal‐oxide (MOD) gate structure in flexible devices (Figure 9f). The combination of the MOD gate with the highly oriented 2D c‐MOF channel facilitates efficient ion transport through vertical nanopores, resulting in ultrahigh transconductance (up to 20.2 µS) and fast response speed (10 µs). This design exploits the porous structure of MOFs to enhance volumetric capacitance (67.8 F cm^−3^), overcoming the limitations of conventional gate dielectrics.

Although materials such as PET, PI, and PEN exhibit good processability and certain mechanical flexibility and stability, they often fail to meet the higher demands of flexibility under extreme stretching or frequent deformation. Consequently, researchers have gradually shifted their focus to substrate materials with more rubber‐like properties, such as PDMS. Its unique silicon–oxygen backbone imparts an ultralow Young's modulus (ranging from tens to hundreds of kPa), comparable to or even lower than that of human skin, thereby enabling a more natural mechanical match with the skin. However, achieving high‐resolution and high‐throughput patterning on PDMS surfaces remains challenging, primarily due to the poor adhesion of photoresists to elastomeric surfaces and substrate swelling induced by organic solvents.^[^ ^157^, ^158^, ^159^ ^]^ To address this, Zhang et al. proposed a novel Parylene‐based transfer patterning process.^[^ ^156^ ^]^ Photolithography and reactive ion etching were first conducted on a PET surface, followed by the transfer of the patterned Parylene film onto PDMS to form a seamless metal deposition mask (Figure 9i). This method not only overcomes the difficulty of lifting off Parylene on PDMS but also achieves metal electrode patterns with a minimum spacing of 5 µm and an almost 100% fabrication yield (Figure 9j). More importantly, the entire process is carried out at room temperature and in a dry state, offering excellent scalability and reusability potential.

##### Stretchable Channel Materials

Although organic semiconductors are intrinsically flexible, as the core component of OECTs, the organic channel materials should exhibit both inherent stretchability and high carrier transport properties for applications in tissue interfaces. The former further requires that semiconductors maintain an effective charge transport pathway during mechanical deformation. **Table** **4** lists several commonly used channel materials for OECTs, along with their mechanical and electrical properties. Among them, PEDOT:PSS is widely accepted due to its high processability, high ionic and electronic mobility, and excellent biocompatibility.^[^ ^36^ ^]^ Therefore, it is necessary to consider the trade‐off between enhancing the deformability of PEDOT:PSS during blending and the potential reduction in ion/electron transport efficiency. To achieve high conductivity, fillers are often added, but this can increase the Young's modulus, which negatively affects stretchability.^[^ ^160^, ^161^, ^162^ ^]^ For example, by adding dimethyl sulfoxide (DMSO) into PEDOT:PSS films, the electrical conductivity increased from 0.3 to 360 S cm^−1^, but the addition of DMSO significantly raised the Young's modulus from 10 to 1000 MPa, resulting in films that were too stiff and exhibited poor stretchability.^[^ ^163^ ^]^ In contrast, achieving high stretchability often requires the addition of insulating components, which can reduce the material's conductivity. For instance, to improve mechanical performance, Kim et al.^[^ ^164^ ^]^ incorporated nonionic waterborne polyurethane (WPU) into PEDOT:PSS, enabling the films to withstand 50% strain at 0.5 wt% WPU and 100% strain at 2.0 wt%. However, this enhancement came at the cost of conductivity: as WPU content increased from 0.1 to 10 wt%, the electrical conductivity dropped sharply from 655.0 to 45.4 S cm^−1^.

**Table 4:** Mechanical and electrical properties of some stretchable conductive polymers.

	Strain tolerance [%]	Intrinsic carrier mobility [cm^2^/V·s]	Dynamic performance	Refs.
PEDOT:PSS	≥ 50	1.1	1 cm^2^ V^−1^ s^−1^ at 50% strain	[165]
p(g2T‐T)	200	0.93	At 200% strain: ‐Parallel to charge transport: 1.58 cm^2^ V^−1^ s^−1^ ‐Perpendicular to charge transport: 0.31 cm^2^ V^−1^ s^−1^ Mobility remains nearly unchanged after 5000 cycles of 100% strain (parallel direction)	[166]
phs‐h‐DPP‐g2T	200%	/	20% drop in *g*_m,n_ ^a)^ after 10 000 strain cycles	[69]
PEDOT:PSS/PAAMPSA/IL	800%	/	Performance retention > 95% after 1500 cycles at 30% strain	[137]
PEDOT:PSS/IL	60%	/	After self‐healing, withstands up to 510% strain; retains > 80% performance	[167]

^a)^ Normalized transconductance.

To balance the conductivity and flexibility of PEDOT:PSS, Savagatrup et al.^[^ ^168^ ^]^ systematically studied the effects of additives such as dimethyl sulfoxide (DMSO), flurosurfactant (Zonyl), and polyethyleneimine (PEI) on the mechanical and electrical properties of PEDOT:PSS. DMSO, as a secondary dopant, promotes the growth and interconnection of PEDOT crystallites, significantly enhancing conductivity to nearly 10^3^ S/cm at a 5% concentration. Zonyl, serving as both a plasticizer and a tertiary dopant, increases the crack‐onset strain to 25%–40% at a concentration of 10%, while also increasing the free volume of polymer chains through hydrogen bonding. By adjusting the ratio of DMSO and Zonyl, a synergistic optimization of the stretchability (40% strain) and conductivity (sheet resistance of 380 Ω sq^−1^) of PEDOT:PSS was achieved. Li et al.^[^ ^169^ ^]^ incorporated low‐molecular‐weight poly(ethylene glycol) (PEG), 5% v/v glycerol, and 1% v/v Capstone into the formulation, which collectively inhibited crack formation in PEDOT:PSS films while preserving high electrical conductivity (∼450 S/cm). At 30% strain, the film's current retention rate remained close to 90%, while at 45% strain, it still retained 75% of the initial current. Additionally, the film's response time was significantly shortened compared to the PEDOT:PSS film without PEG, due to the hydroxyl groups in PEG, which enhanced the ionic mobility.

Ionic liquids (ILs), due to their excellent chemical stability, broad electrochemical window, and high ionic conductivity, have demonstrated potential for optimizing channel materials.^[^ ^170^ ^]^ Specifically, for PEDOT:PSS, p‐type doping of the polymer facilitates the formation of the fiber network, enhancing charge transport properties while improving the material's stability under mechanical deformation. Recently, Pang et al.^[^ ^167^ ^]^ optimized PEDOT:PSS by introducing the biocompatible ionic liquid (IL) P14[TFSI]. They found that adding 0.5 wt% of P14[TFSI] significantly enhanced its electronic conductivity to 740 S/cm and its ionic conductivity to 6×10^−6^ S cm^−1^, and through ion exchange, promoted the crystallization and interconnection of PEDOT nanofibers, resulting in a film stretchability of up to 60%. The stretchable OECT based on this optimized material exhibited high transconductance (2.1 mS), mechanical elasticity (30% strain), and long‐term stability (with transconductance remaining 95% of its initial value after 6 months of storage at room temperature, and the ion gel's mass change less than 3%), achieving a high‐fidelity ECG monitoring with a signal‐to‐noise ratio (SNR) up to 35.7 dB.

It should be noted that although reducing the Young's modulus of channel materials is crucial, the modulus matching between the substrate and the material is equally important. Studies have shown that when PEDOT:PSS films are applied to TPU substrates, they exhibit excellent intrinsic stretchability.^[^ ^165^ ^]^ At 50% strain, the film shows almost no visible cracks and can maintain a high current without relying on plasticizers. However, when the film is applied to other substrates (e.g., PDMS, ethylene‐vinyl acetate copolymer (EVA), SEBS), noticeable cracks appear at lower strains, accompanied by significant current loss. The high intrinsic stretchability is primarily attributed to the improved modulus matching between the TPU substrate and the PEDOT:PSS film. This suggests that optimizing modulus matching helps to effectively distribute stress, reduce microcrack formation, and prevent large‐scale transverse cracks, thus enhancing the intrinsic stretchability of the channel material.

#### 5.2.2 Structural Engineering

**Figure 10:**
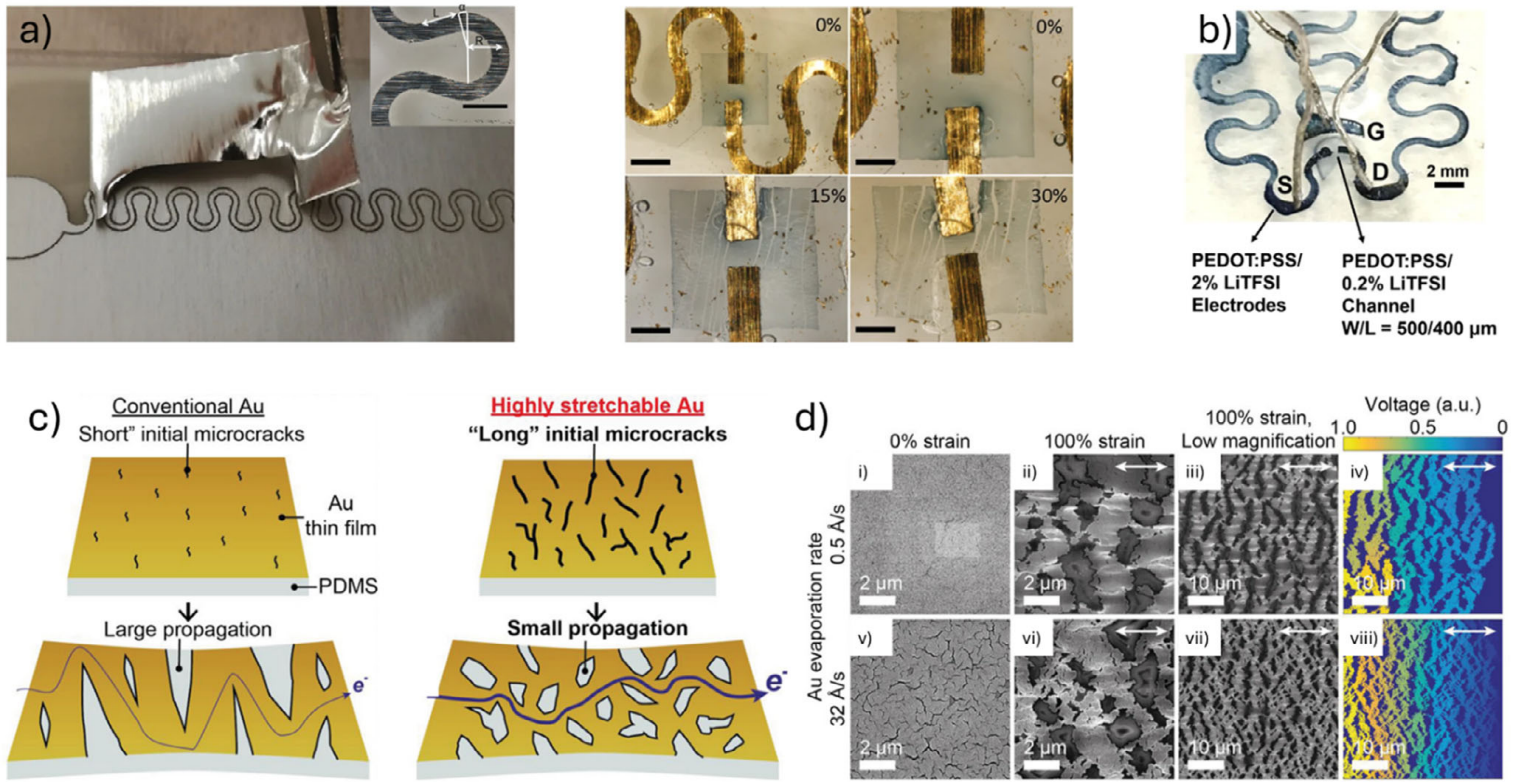
a) Fabrication of metal interconnects by laser‐cutting metal foil adhered to a heat‐release tape (left), and photographs of the channel and electrodes under various strain conditions (right). Reproduced with permission.^[^ ^171^ ^]^ Copyright 2018, Springer Nature. b) Photograph of an all‐polymer OECT configuration, where a submicron‐thick PEDOT:PSS/0.2% LiTFSI channel layer is printed on top of a thick PEDOT:PSS/2% LiTFSI microelectrode. Reproduced with permission.^[^ ^172^ ^]^ Copyright 2022, Springer Nature. c) Schematic illustration of crack propagation under strain in a conventional Au film with short initial microcracks (left) and a highly stretchable Au film with long initial microcracks (right). d) i)–iv) and v)–viii): SEM images of Au thin films deposited at evaporation rates of 0.5 and 32 Å s^−1^, respectively. The Au films deposited at a high rate exhibit numerous long initial microcracks, with minimal crack propagation under strain. c,d) Reproduced with permission.^[^ ^173^ ^]^ Copyright 2019, Wiley.

When OECT devices are intended for highly flexible applications such as skin adhesion and dynamic physiological monitoring, structural design becomes equally critical. As an important discipline parallel to materials engineering, structural engineering focuses on strategies such as strain engineering, device layout optimization, and three‐dimensional structural design to effectively distribute mechanical stress, delay material fatigue, and maintain stable electrical performance under stretching and bending. A primary focus in structural optimization is the development of stretchable interconnections (electrodes), whose performance largely depends on their geometric configuration. In stretchable electronics, the serpentine layout is the most adopted interconnection strategy due to its excellent strain‐buffering capability, achieved by absorbing deformation through the uncoiling of curved arcs. For instance, Marchiori et al.^[^ ^171^ ^]^ optimized the geometric parameters of electrode routing—including arc angle α (routing angle), arc radius R, and the connecting length L between arcs—to achieve maximum stretchability (**Figure** **10**a (left)). The resulting metallic interconnects exhibited up to 70% stretchability with ultralow milliohm‐level resistance. The electrodes were subsequently embedded into and cured within a PDMS layer, thermally released, and then laminated in an inverted orientation onto a glass substrate. A final spin‐coating process was used to fully encapsulate the device within a PDMS packaging layer. The fabricated OECT retained approximately 77% of its initial transconductance (6.5 mS) and over 85% of its initial output current at 11% strain. However, when the strain was further increased to 38%, the transconductance sharply dropped to 0.35 mS, approximately 5.4% of its original value. This degradation is likely due to delamination between the electrodes and the channel, caused by the high rigidity of aluminum and the excessive interconnect thickness (700 µm) used in the device (Figure 10a (right)).

To address the issue of structural mismatch, recent studies have shifted toward the development and application of conductive polymer electrodes. For example, Wang et al.^[^ ^172^ ^]^ employed a commercial PEDOT:PSS solution, doped with 2 vol% LiTFSI solution and further modified by adding 0.5% of the surfactant FS‐31 to improve wettability. After vacuum degassing, the resulting ink was deposited onto a glass substrate via blade coating and dried at 60 °C. The film was then transferred onto a pre‐stretched SEBS elastomer, followed by stress release, which induced the formation of self‐assembled wrinkled microelectrodes (Figure 10b). This structure can unfold during stretching to absorb external strain, enabling geometric strain buffering and thereby preserving the interfacial integrity between the electrode and the channel. Through the synergistic design of geometry and materials, the device maintained nearly unchanged electrical performance under 20% uniaxial strain and retained over 80% of its initial transconductance after 1000 strain cycles, demonstrating stable high‐performance output.

Microcracks and bent Au films have garnered particular interest in stretchable electronic devices, as they maintain conductivity during stretching. Controlling the initial microcrack morphology and its propagation in Au electrodes can potentially improve the conductivity under strain and stabilize it (Figure 10c). Matsuhisa et al.^[^ ^173^ ^]^ further proposed that by modulating the initial microcrack morphology during the Au film deposition process, the conductive stability at high strains can be significantly enhanced. They used a high evaporation rate (32 Å s^−1^) to deposit a 40 nm Au film, generating high aspect ratio initial microcracks, which effectively suppressed strain‐induced crack propagation (Figure 10d). Compared to traditional low‐rate deposition methods (0.5 Å s^−1^), the optimized Au film exhibited a sheet resistance of only 33.3 Ω sq^−1^ at 120% strain, making it one of the best‐performing microcracked Au conductors reported to date. OECTs employing these highly stretchable Au electrodes showed a high transconductance (*g*_m_) of 0.54 mS at 0% strain, maintaining 0.14 mS of *g*_m_ even at 140% strain.

**Figure 11:**
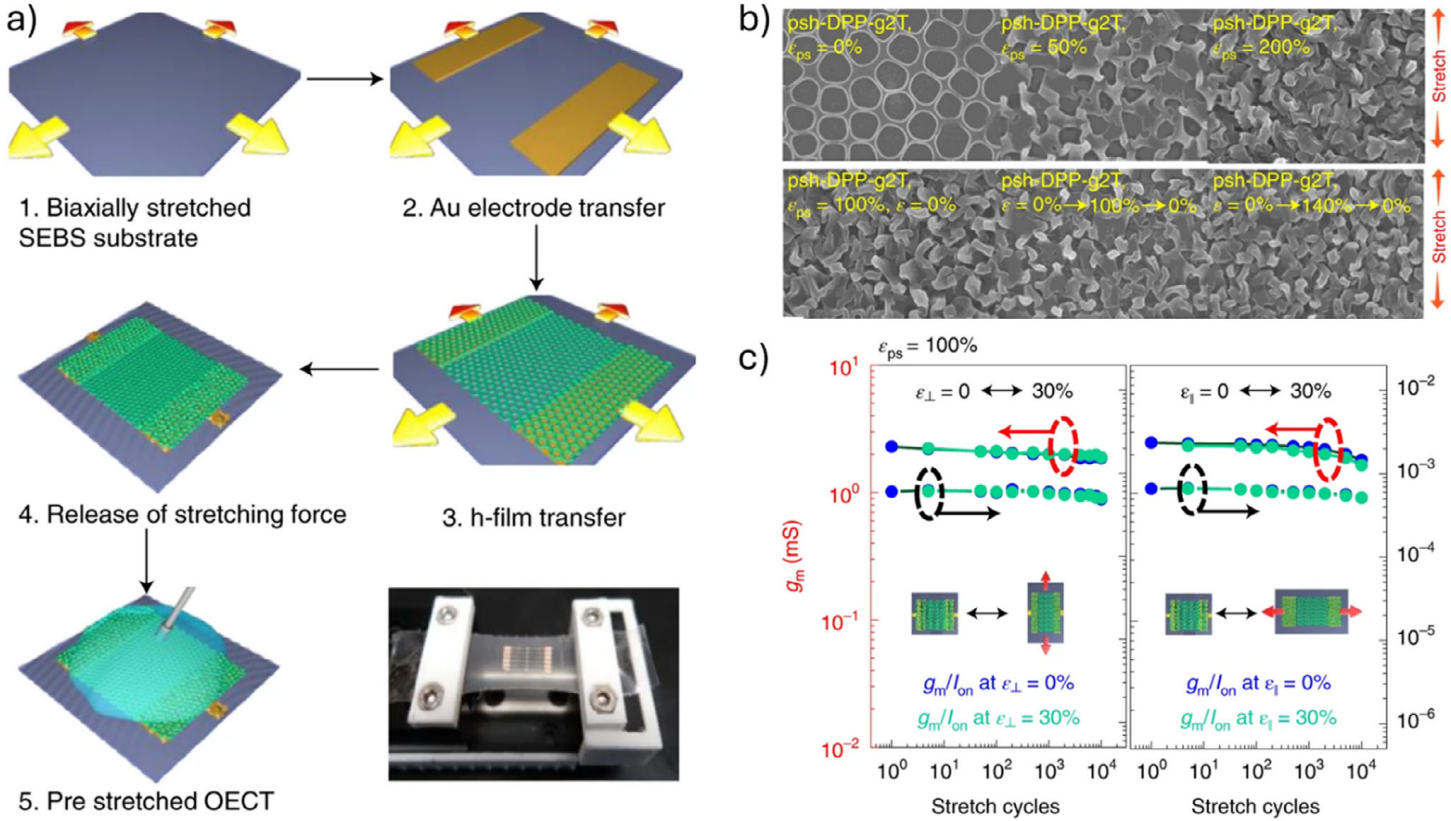
a) Schematic illustration of the fabrication of a prestretched OECT on a biaxially stretched SEBS substrate; b) SEM images of the h‐DPP‐g2T thin film under various tensile strains, showing the microstructural evolution; c) Dependence of transconductance (*g*_m_) and on‐current (*I*_on_) on the number of stretching cycles for psh‐DPP‐g2T OECTs (*ε*_ps_ = 100%) in both perpendicular (*ε*_⊥_) and parallel (*ε_∥_*) directions. Reproduced with permission.^[^ ^69^ ^]^ Copyright 2022, Springer Nature.

Furthermore, constructing wrinkled structures on the surface of flexible or stretchable substrates via pre‐stretching or molding is a simple and effective strategy to enhance stretchability. For the former, the fabrication of stretchable organic transistors has been reported on pre‐strained elastomers such as PDMS,^[^ ^156^, ^174^ ^]^ SEBS,^[^ ^175^ ^]^ and 3 M VHB.^[^ ^128^ ^]^ Someya et al.^[^ ^128^ ^]^ pioneered this approach by developing ultraflexible organic field‐effect transistors (OFETs) on prestretched 3 M VHB substrates, where wrinkled topologies were formed. Building on this principle, Zhang et al.^[^ ^156^ ^]^ demonstrated a patterned OECT fabrication strategy using prestretched PDMS substrates. By prestraining the PDMS and subsequently depositing the active PEDOT:PSS layer, followed by gradual release of the substrate, periodic uniaxial wrinkles were generated across the surface. These wrinkles not only served as effective strain buffers but also contributed to channel formation, facilitating patterning and simplifying the device fabrication process. To further enhance both mechanical stretchability and electrical stability, Chen et al.^[^ ^69^ ^]^ proposed a novel strategy by integrating honeycomb‐structured semiconducting films with a biaxially prestrained SEBS substrate (**Figure** **11**a). In this approach, the active layer (h‐DPP‐g2T) was first fabricated as a free‐standing porous film and subsequently transferred onto a prestrained elastomer embedded with pre‐patterned Au electrodes. Upon release of the applied strain, the device surface developed well‐aligned wrinkled topographies without distorting the geometry of the active channel. This design effectively decoupled mechanical deformation from the channel, thereby minimizing electrical performance degradation during stretching. As confirmed by SEM analysis (Figure 11b), the wrinkled honeycomb film demonstrated superior morphological stability compared to its dense‐film counterpart. Electrical measurements further revealed that the prestretched OECTs maintained stable transconductance and on‐state currents under high strains (up to 140%) and during repeated stretching cycles, making them promising candidates for mechanically resilient bioelectronic applications (Figure 11c).

**Figure 12:**
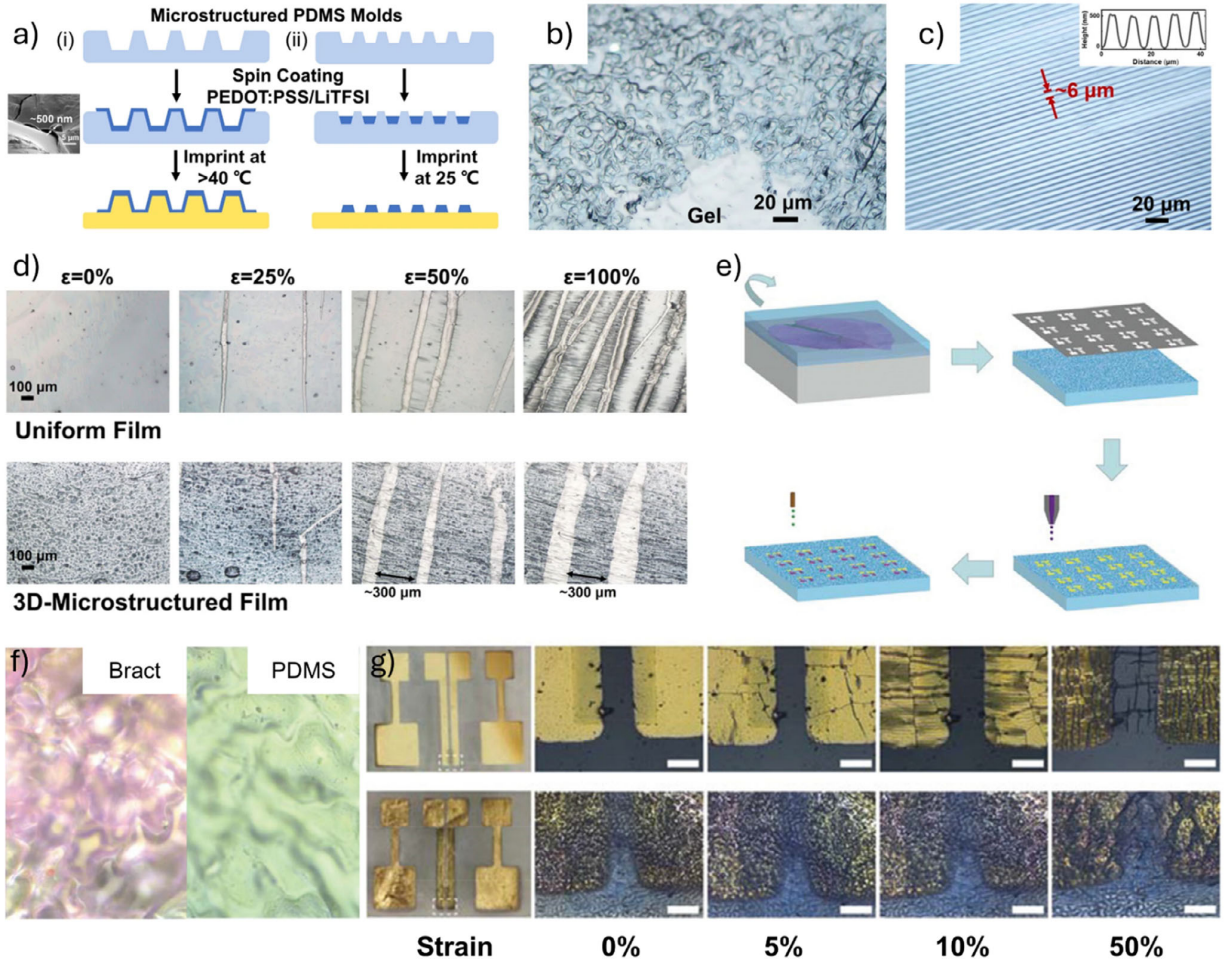
a) Schematics of PEDOT:PSS/LiTFSI films with (i) 3D microstructured and (ii) microwire morphologies on the GEL–GLY/Na_3_Cit surface, fabricated by imprinting with PDMS molds; b),c) Corresponding optical microscope images of the random sandpaper template and photolithographically defined microwire template, respectively; d) Optical microscope images showing strain‐induced morphological changes of uniform and 3D microstructured PEDOT:PSS/LiTFSI films under strains ranging from 0% to 100%. a–d) Reproduced with permission.^[^ ^172^ ^]^ Copyright 2022, Springer Nature. e) Schematic illustration of PDMS molding using bract texture and the fabrication process of OECT devices; f) Photographs of the bract (left) and molded PDMS substrate; g) Morphological changes of a conventional OECT (top) and a stretchable OECT (bottom) under increasing strain. Scale bar: 200 µm. e–g) Reproduced with permission.^[^ ^155^ ^]^ Copyright 2019, Wiley.

In the case of molding, periodic micro/nanostructures with undulating profiles can be replicated onto the substrate surface using a template. These structures can geometrically unfold under strain to absorb deformation, thereby preventing excessive strain in critical device layers.^[^ ^7^ ^]^ For example, Wang et al.^[^ ^172^ ^]^ patterned a PEDOT:PSS/LiTFSI conductive polymer film using PDMS molds with microstructured surfaces—either a random sandpaper template (**Figure** **12**b) or photolithographically defined microline patterns (Figure 12c)). The patterned film was then thermally transferred onto the surface of a gelatin–glycerol–trisodium citrate (GEL–GLY/Na_3_Cit) hydrogel electrolyte, forming a 3D wrinkled conductive layer with a honeycomb‐like topology (Figure 12a). Compared to traditional flat structures, the 3D molded design maintained a continuous conductive path under strains exceeding 50%, exhibiting lower crack density and shorter crack lengths (Figure 12d). Device performance also benefited from the structural stability, retaining approximately 84% of the source‐drain current (*I*_ON_) and 70%–80% of the peak transconductance (*G*_m_) under 100% biaxial strain. More importantly, after 1000 cycles at 80% strain, the device still maintained ∼95% of *I*_ON_ and ∼75% of the maximum *G*_m_. Our group proposed a novel method for fabricating stretchable OECTs based on a bioinspired PDMS substrate.^[^ ^155^ ^]^ The substrate pattern was transferred from the bract of *Bougainvillea glabra*, presenting a two‐dimensional wavy structure (Figure 12f). This naturally occurring micro‐wrinkled morphology was successfully replicated onto the PDMS surface via soft lithography, forming a highly isotropic undulating structure with an average structural ratio (A₀/λ) of approximately 0.13, effectively mitigating strain concentration (Figure 12e). Under varying tensile strains (ranging from 0% to 30%), the electrode structure of the bioinspired OECT device demonstrated excellent strain‐buffering capability. Within the 10%–20% strain range, almost no visible cracks were observed in the electrodes. In contrast, conventional OECTs fabricated on flat PDMS substrates exhibited severe cracking at just 5% strain and complete device failure at 20% strain (Figure 12g). This bioinspired OECT was further applied as a conformable glucose sensor, achieving a detection limit as low as 1 µM, showing great potential for wearable blood glucose monitoring.

## 6 In Vivo‐OECT Interfaces

When deployed within living organisms, OECTs face more complex challenges than those encountered in epidermal applications. These challenges encompass immune compatibility, long‐term stability, and mechanical integration with internal soft tissues. Achieving effective in vivo integration necessitates the coordinated optimization of materials, device architecture, and implantation strategies to minimize foreign body reactions while maintaining electrical performance. Several critical factors must be considered to achieve this integration, including bioresorbability, long‐term stability, and material compatibility with clinical sterilization processes. This section reviews recent advancements in engineering biocompatible and conformal interfaces, developing miniaturized and biodegradable platforms, and implementing strategies for minimally invasive implantation. Each of these advancements contributes to the safe and sustained operation of OECTs in physiological environments.

### 6.1 Implementation Considerations for In Vivo Applications

#### 6.1.1 Bioresorbability and Long‐Term Stability

##### Biocompatible Interfaces

The requirements for in vivo interfaces closely resemble those of skin interfaces, particularly regarding mechanical compliance and biocompatibility. Mechanical compliance refers to the ability of the material used in the OECT to deform and conform to the soft, dynamic nature of biological tissues without inducing stress or damage. However, implantable systems must meet more stringent requirements, as they are directly exposed to fragile, dynamic internal tissues and the host immune system. As summarized in **Table** **5**, the mechanical properties of various tissues impose critical constraints on device design, particularly with regard to minimizing mechanical mismatch. Achieving a truly biocompatible interface requires more than inert material properties; it also necessitates modulating immune responses, such as suppressing inflammation, minimizing fibrotic encapsulation, and promoting immune tolerance. Both mechanical compliance and biocompatibility are essential for ensuring that the OECT does not induce inflammation or immune rejection upon implantation. To this end, ultra‐soft, tissue‐compliant substrates with minimal structural footprints are critical to ensuring seamless integration and minimizing chronic immune rejection. Additionally, both the substrate and active components must be compatible with clinical sterilization protocols without compromising their mechanical or electronic performance, which is vital for the safe and effective in vivo deployment of OECTs.

**Table 5:** Key mechanical properties of tissues for biomedical device design.

Tissue	Mechanical properties			Dynamics	Refs.
	Young's modulus		Strain range		
	[kPa] [indentation]	[MPa] [tensile]			
Cardiac	18 ± 2	/	10%–20%	∼40–200 contractions/minute	[176]
Gastrointestinal tract	/	1×10^−3^–1	140%–190%	7–20 contractions/minute	[177, 178, 179, 180]
Central nervous system	0.2–7	∼2	/	/	[181]
Liver & kidney	∼190	∼10	liver: 1%–5% kidney: 1%–25%/s	/	[181, 182]
Blood vessels	∼125	∼2	artery: 10%–30% vein: 100%–150%	/	[181, 183, 184]
Muscle	∼7	∼480	10%–20%	/	[181]
Tendon	/	∼560	105–20%	/	[181]
Cornea	∼29	∼3	/	/	[181]
Fat	/	5×10^−4^–3×10^−3^	/	/	[181, 185]

##### Bioresorbable OECTs

Biodegradable materials, whether natural or synthetic, are capable of degrading in vivo. This degradation occurs via enzymatic processes, non‐enzymatic mechanisms, or a combination of both. During this process, they generate biocompatible, non‐toxic byproducts, which are subsequently cleared through standard metabolic pathways.^[^ ^15^ ^]^ **Table** **6** lists examples of bioresorbable materials for electronics. Among various biomaterials, the biodegradable copolymer poly(lactic‐co‐glycolic acid) (PLGA) has shown considerable promise as a carrier for drug delivery and as a scaffold in tissue engineering. Its suitability for such biomedical applications is underscored by U.S. FDA approval for drug delivery use.^[^ ^77^ ^]^ Building on PLGA, Yu et al. proposed an ultra‐thin, flexible, biodegradable OECT array.^[^ ^75^ ^]^ This device integrates a 100‐channel array with PLGA as the substrate and insulation layer, Au electrodes, and a PEDOT:PSS channel, with a total thickness of only 15 µm. The array can form conformal adhesion with the curved surface of the brain (Figure 16d), thanks to its mechanical flexibility with a minimal bending radius of less than 5 mm (Figure 16c). Moreover, the entire device underwent accelerated testing in a 90 °C PBS solution and was able to degrade into micro/nanoparticles within 4 hours (Figure 16a). Importantly, the insulation provided by PLGA effectively isolates the Au electrodes, with Au dissolution levels remaining below 1.74 µg L^−1^, significantly enhancing biological safety. The array was seamlessly applied to the rat cortical surface for high‐fidelity micro‐ electrocorticogram (µ‐ECoG) recording, achieving an SNR of up to 37 dB with cellular‐level resolution.

**Table 6:** Examples of bioresorbable materials for bioelectronics.

Components	Material	Dissolution rate	Refs.
Substrate	Silk	∼8 µm min^−1^ in PBS	[189]
	Cellulose, gelatin	/	[190]
	Rice paper	/	[191]
	Levan polysaccharide	∼120 min in DI water	[186]
	Poly lactic‐co‐glycolic acid (PLGA)	∼10µmday^−1^ for 50:50 monomer ratio	[77]
	Diacetate cellulose	∼50s in 90% acetic acid solution	[187]
	Polylactic acid (PLA)	/	[188]
Conductor	Magnesium (Mg)	4.8 ± 2.2 µm h^−1^	[192]
	Zinc (Zn)	0.3 ± 0.2 µm h^−1^	
	AZ31B (zinc alloy)	2.6 ± 2.1 µm h^−1^	
	Iron (Fe)	∼7 × 10^−3^ µm h^−1^	
	Molybdenum (Mo)	(7 ± 4) × 10^−4^ µm h^−1^	
	Tungsten (W)	2 × 10^−3^ µm h^−1^	
Semiconductor	Silicon (Si)	4.5 nm day^−1^	[192, 193]
	*i*‐3gTIT	/	[47]

**Figure 13:**
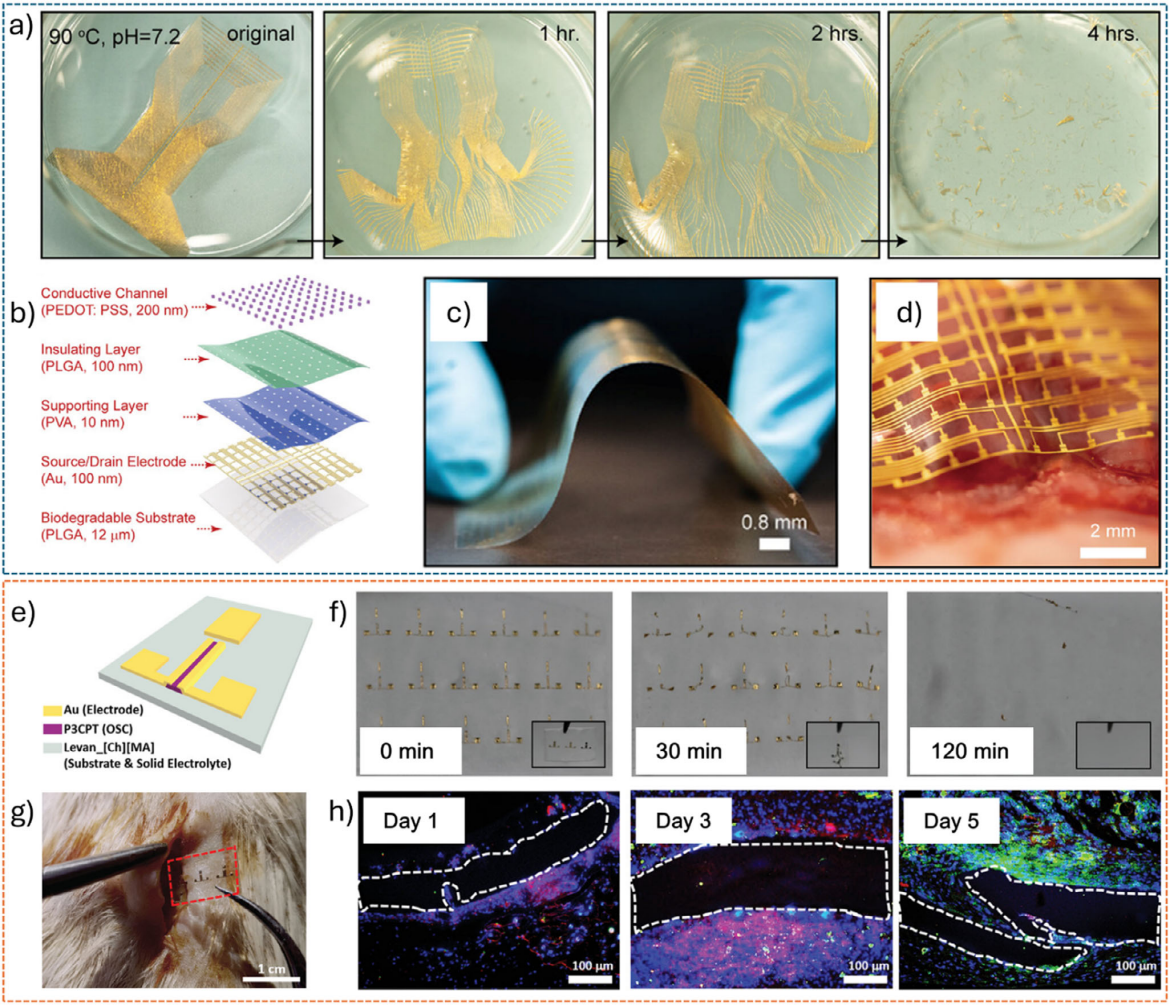
a) Accelerated degradation of a biodegradable OECT in PBS solution at 90 °C; b) Schematic diagram of a fully biodegradable OECT array with compatible mechanical flexibility c) and ultra‐thin and high‐density d). a)‐d) Reproduced with permission.^[^ ^75^ ^]^ Copyright 2023, Wiley. e) Biodegradable OECT using levan polysaccharide and ionic liquid ([Ch][MA]) as the substrate and dielectric material; f) Photographs of the water solubility and physical transient states of the biodegradable transistor immersed in deionized water; g) Photographs showing the implantation of OECT into a rat body; h) Immunofluorescence staining images after OECT implantation. The white dashed lines indicate the area of the OECT. e)‐h) Reproduced with permission.^[^ ^186^ ^]^ Copyright 2020, Wiley.

Some edible materials can also be utilized to construct solid‐state electrolytes and substrates. For example, Kim et al.^[^ ^186^ ^]^ developed a composite system composed of levan polysaccharide and a choline‐based ionic liquid ([Ch][MA]) (**Figure** **13**e). Levan, a naturally water‐soluble polysaccharide, and [Ch][MA], synthesized from choline (an essential nutrient) and malic acid (a natural organic acid), form a free‐standing solid‐state electrolyte (LSE) through simple solution blending, serving as both the substrate and the dielectric layer. The LSE film, with a thickness of approximately 100 µm and over 98% transmittance in the visible light range, exhibits outstanding mechanical resilience, tolerating up to 1.11% bending strain and 5% tensile strain. It achieves a transconductance of 2.0 mS and an on/off ratio of 10^3^ under a low voltage of −2.0 V. In this system, levan gradually hydrolyzes into monosaccharides via glycosidic bond cleavage, while [Ch][MA] dissolves in water, allowing for complete degradation within 2 hours in deionized water (Figure 13f). The degradation byproducts, such as malic acid and choline, can be metabolized through natural biological pathways. Rat implantation experiments (Figure 13g) showed no long‐term inflammation, and immunofluorescence confirmed the dominance of M2 macrophages in the repair process, suggesting that the material can safely participate in tissue remodeling (Figure 13h). Efforts have also focused on utilizing biodegradable and compostable substrates to advance sustainable OECT platforms. Granelli et al.^[^ ^187^ ^]^ introduced a fully printed, mask‐less fabrication strategy for OECTs integrated on thin‐film diacetate cellulose—a biomass‐derived, biodegradable, and industrially compostable material. This substrate, sourced from non‐edible plant fibers, demonstrates rapid degradation in natural environments and industrial composting systems, with dissolution occurring in under 1 min when exposed to mild solvents like acetic acid. The entire fabrication process minimizes chemical waste by avoiding photolithography, relying instead on additive manufacturing techniques such as dispensing and capillary printing. Despite the use of eco‐friendly materials and low‐temperature processes, the resulting OECTs retain excellent electronic properties, including low‐voltage operation, high transconductance, and a normalized inverter gain up to 136.6 V^−1^. Furthermore, the fully printed devices enable high‐sensitivity ion sensing (506 mV dec^−1^), illustrating that biodegradability does not necessarily compromise performance. Fumeaux et al.^[^ ^188^ ^]^ adopted a complementary strategy by developing disposable OECTs based on water‐soluble conducting polymers and a cellulose‐based paper substrate. Designed specifically for single‐use biochemical sensing, their devices could disintegrate upon disposal, aligning with ecological safety regulations for point‐of‐care diagnostics.

Further advancing degradable active materials, Makhinia et al.^[^ ^113^ ^]^ presented a fully printed accumulation‐mode OECT architecture using degradable semiconducting blends and glycerol‐based gel electrolytes. By fine‐tuning the interface between the semiconducting layer and the electrolyte, their devices achieved favorable transconductance and switching behavior, while ensuring component‐level degradation under mild aqueous conditions. Most recently and notably, Chen et al.^[^ ^47^ ^]^ introduced imine‐linked polymeric OMIECs (*i*‐3gTIT and *o*‐3gTIT) that simultaneously deliver excellent mixed ionic–electronic conduction and programmable degradation. By modulating backbone regiochemistry, they engineered *i*‐3gTIT to exhibit superior charge mobility (1.99 cm^2^ V^−1^ s^−1^), enhanced crystallinity, and rapid acid‐triggered degradation. Importantly, the material also demonstrated good biocompatibility in cell culture assays and could function as a transient synaptic element in neuromorphic computing platforms.

##### Minimally Invasive OECT

Minimizing the physical invasiveness of implantable electronics is essential to reducing tissue damage and enabling stable, long‐term recording of sensitive biological signals. In neural interfaces, where OECTs serve as miniaturized amplifiers of ionic activity, complementary transistor pairs with matched threshold voltages and transconductance are often needed. However, variability between n‐type and p‐type OECTs typically results in the need for millimeter‐scale devices, limiting spatial resolution.^[^ ^194^, ^195^ ^]^ Owing to the fabrication flexibility of OMIECs, an important strategies have emerged: vertical OECT designs, which reduce the device footprint by orienting the channel perpendicular to the substrate.

**Figure 14:**
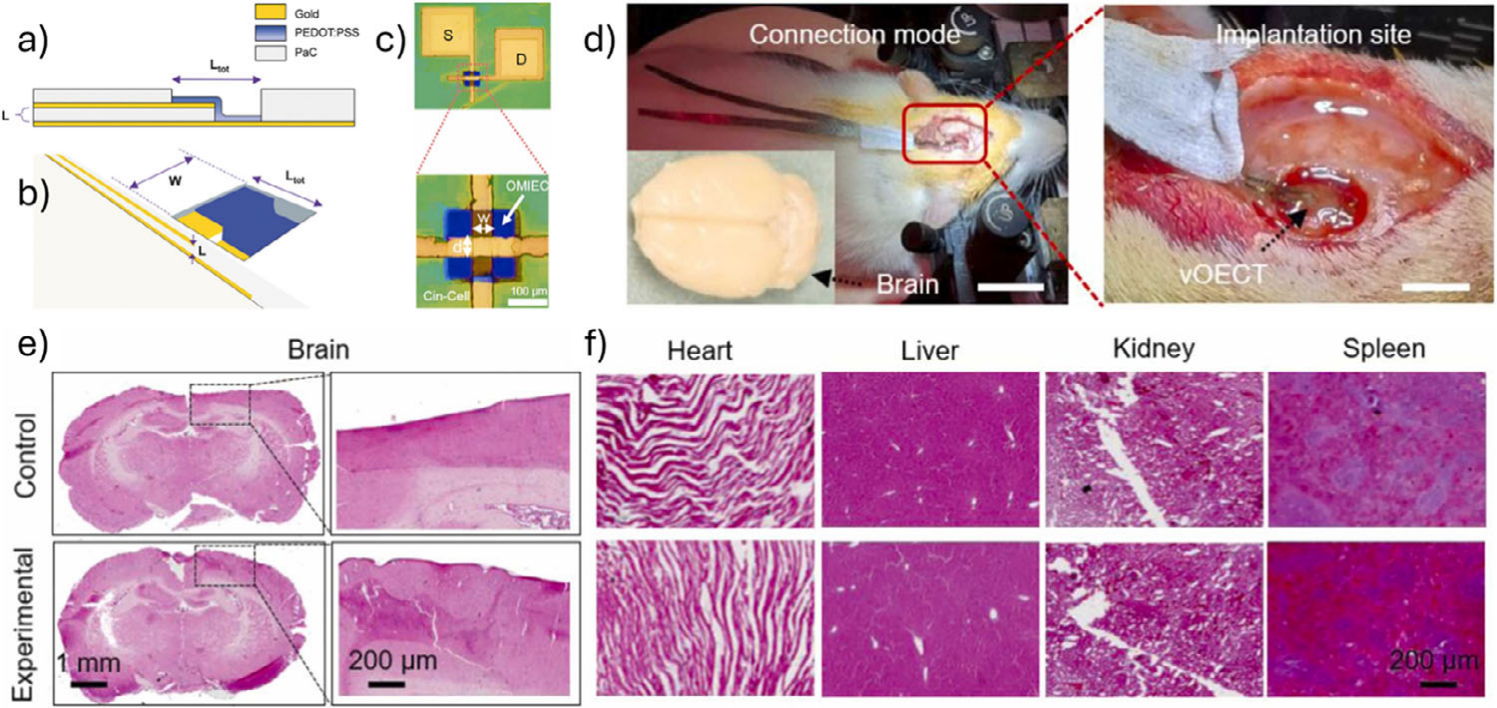
a) Cross‐sectional and b) top‐down illustrations of the structural layout of vertical organic electrochemical transistors (vOECTs). a,b) Reproduced with permission.^[^ ^78^ ^]^ Copyright 2018, Wiley. c) Optical image of the fabricated vOECT; d) Connection mode of implantable vOECT (left) and location of implantable vOECT in regions of the rat cortex (right); e) Hematoxylin and eosin (H&E) staining of brain tissue; f) H&E‐stained sections of major organs including the heart, liver, kidney, and spleen. c–f) Reproduced with permission.^[^ ^199^ ^]^ Copyright 2024, Elsevier.

Vertical organic electrochemical transistors (vOECTs) achieve a smaller device size by orienting the channel perpendicularly to the substrate. This configuration not only reduces the device footprint, minimizing tissue disruption during implantation, but also improves overall integration with the biological environment, thereby enhancing biocompatibility. As shown in **Figure** **14**a,b, the channel length (L) in a vOECT is determined by the thickness of the dielectric layer separating the source and drain electrodes arranged in a vertical configuration.^[^ ^78^ ^]^ This configuration enables sub‐micrometer resolution, which exceeds the approximately 5 µm resolution typically achievable with conventional fabrication techniques. The architecture also substantially reduces the device footprint, with the area of a single inverter decreasing by approximately 50% compared to that of conventional OECTs (cOECTs), which significantly minimizes tissue damage during implantation. In addition, it improves current density, transconductance, and response speed.^[^ ^196^, ^197^ ^]^ This vertical design not only maintains high electrical performance but also allows for higher integration density and improved strain tolerance,^[^ ^198^ ^]^ thereby enhancing the potential of OECTs in bioelectronic interface applications. For example, Gao et al.^[^ ^199^ ^]^ recently presented a compelling demonstration of the minimally invasive capabilities of vOECTs through the development of ultraflexible devices employing a high‐loading, homogeneous crosslinking strategy. By incorporating a diaziridine‐functionalized small‐molecule crosslinker (DtFDA), the authors achieved the formation of face‐on‐oriented, low‐crystallinity OMIEC channels, which promoted efficient ionic–electronic coupling. The resulting vOECTs exhibited high transconductance values exceeding 0.34 S, sub‐millisecond response times, and exceptional operational stability over 200 000 cycles. Importantly, the vertical device architecture enabled sub‐100 nm channel lengths while maintaining low‐voltage, low‐hysteresis characteristics under both thermal and electrochemical stress conditions. The vOECTs were monolithically integrated onto parylene substrates and chronically implanted into the rat somatosensory cortex (Figure 14d). Upon limb stimulation, the devices recorded high‐fidelity electrocorticographic (ECoG) signals, which remained stable over a four‐week implantation period. Furthermore, histological analyses of brain tissue and major organs (Figure 14e,f) revealed negligible local inflammation and no observable systemic toxicity, collectively confirming the long‐term biocompatibility and safety of this vertical bioelectronic platform.

vOECTs hold great promise for enhancing device performance. However, their continued development faces notable challenges, particularly in achieving precise channel patterning and aligning the crystallographic orientation of organic materials with the device architecture.^[^ ^200^, ^201^ ^]^ On one hand, vOECTs demand well‐defined channel regions to minimize parasitic capacitance and support high integration density, yet traditional photolithographic techniques often fall short in resolution. To address this, Lai et al.^[^ ^202^ ^]^ introduced a highly reactive diazirine‐based photo‐crosslinker, double‐end trifluoromethyl diazirine (DtFDA), which selectively crosslinks with the side chains of organic semiconductors without requiring photoinitiators. This approach enabled high‐resolution patterning with a minimum feature size of 2 µm in materials such as p(g2T‐T) and Homo‐gDPP. On the other hand, the vertical charge transport pathway in vOECTs benefits significantly from semiconductors with a face‐on molecular orientation.^[^ ^203^, ^204^, ^205^ ^]^ By applying thermal annealing to induce vertical face‐on stacking in a newly developed small‐molecule semiconductor, IDIC‐MEG, researchers achieved a record transconductance of 46.3 mS in vOECTs, underscoring the critical role of orientation–structure alignment in device optimization.^[^ ^204^ ^]^ To facilitate comparison across the various vOECT and vIGT architectures discussed, **Table** **7** provides a summary of the key performance metrics, including but not limited to transconductance, response time, stability, integration density, and other relevant device characteristics.

**Table 7:** Comparative table of key metrics for vOECT and vIGT devices.

Device Type	Architecture /Material	Channel Length	Response Time	*g*_m_	Stability	Integration Density	Refs.
vOECT	Sub‐100 nm vertical OMIEC channel with DtFDA crosslinker	< 100 nm	< 1 ms	> 0.34 S	> 200 000 cycles	/	[199]
F‐vOECT	Coaxial vertical structure; p‐type channel: p(g2T‐T) n‐type channel: BBL	∼3 µm	p‐type: 0.138 s n‐type: 2.777 s	p‐type: 41.10 mS n‐type: 2.25 mS	> 500 cycles	/	[206]
F‐vOECT	Coaxial vertical structure; Channel: PEDOT:PSS	∼3 µm	12 ms	16 mS	> 14 days in PBS; > 7 days in vivo;	/	[207]
vIGT	H‐via vertical hydration conduit; p‐type channel: PEDOT:PSS/d‐sorbitol n‐type channel: PEDOT:PSS/PEI	800 nm	0.9 µs	18.67 ± 1.90 mS	>1 year in physiological media	155 586 transistors cm^−2^	[208]

#### 6.1.2 Sterilization Compatibility

**Figure 15:**
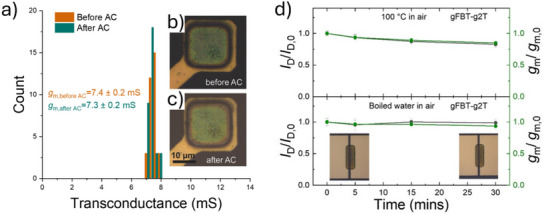
a) Distribution of OECT transconductance before and after autoclave sterilization; b, c) Optical microscopy images of the same microelectrode before b) and after c) autoclave treatment. a–c) Reproduced with permission.^[^ ^73^ ^]^ Copyright 2016, Wiley. d) Device stability of gFBT‐g2T OECTs under different annealing durations: exposed to air at 100 °C (top) and immersed in boiling water (bottom), showing variations in saturation current and transconductance. Inset: optical images of the same channel before and after 30 minutes of immersion in boiling water. Reproduced with permission.^[^ ^46^ ^]^ Copyright 2025, Wiley.

In addition to biocompatibility and mechanical adaptability, another crucial yet often overlooked requirement for implantable OECTs is their compatibility with clinical sterilization protocols. Ensuring sterility is a prerequisite for any device intended for in vivo use, especially in clinical or surgical environments. However, this poses a significant challenge for OECTs, as many organic electronic materials exhibit high sensitivity to heat, humidity, and oxidative environments commonly associated with standard sterilization procedures. Notably, Uguz et al.^[^ ^73^ ^]^ demonstrated that PEDOT:PSS‐based OECTs can withstand standard autoclave sterilization (121 °C for 20 min) without compromising their functional integrity. Optical microscopy images (**Figure** **15**b,c) showed no discernible morphological changes in the microelectrodes before and after sterilization, confirming structural stability. Importantly, the electrical performance was largely preserved: the average transconductance decreased only slightly from 7.4 ± 0.2 to 7.3 ± 0.2 mS, indicating that the devices’ signal amplification capability remained virtually unaffected (Figure 15a). Recently, Liao et al.^[^ ^46^ ^]^ developed a polymeric mixed conductor—gFBT‐g2T—that exhibits intrinsic resistance to autoclave sterilization. Instead of evaluating only fully assembled devices, they systematically examined the physicochemical and electrical stability of the polymer films themselves. Remarkably, gFBT‐g2T retained over 95% of its initial electrical conductivity after five autoclave cycles (121 °C, 15 psi, 20 min) (Figure 15d), in contrast to conventional PEDOT:PSS formulations, which typically undergo significant degradation upon repeated sterilization. Spectroscopic analyses further confirmed the preservation of the polymer's doping state and structural conformation, demonstrating excellent retention of its mixed ionic–electronic conduction properties.

### 6.2 Electrical Signal‐OECT Interfaces

Based on the previously discussed core considerations for in vivo OECT implementation—mechanical matching, biocompatibility, minimally invasive design, and biodegradability—this section focuses on the specific implementation of electrical signal sensing interfaces. Electrical signals (e.g., ECG, ECoG), being the most easily captured physiological signals in vivo, require interface designs that strictly adhere to the principles of “minimizing tissue interference and enhancing signal fidelity.” For instance, using biocompatible substrates (e.g., parylene, PLGA) to reduce immune rejection, minimizing tissue damage through minimally invasive forms (e.g., ultra‐thin arrays, vertical structures), and reapplying the “mechanical matching” strategy (e.g., SEBS elastic substrates adapted to muscle contractions) to ensure long‐term signal stability. The following section will detail the design logic and application cases of interfaces according to signal types (ECG, ECoG, and other electrical signals) in different scenarios.

#### 6.2.1 ECG Sensing Interfaces

**Figure 16:**
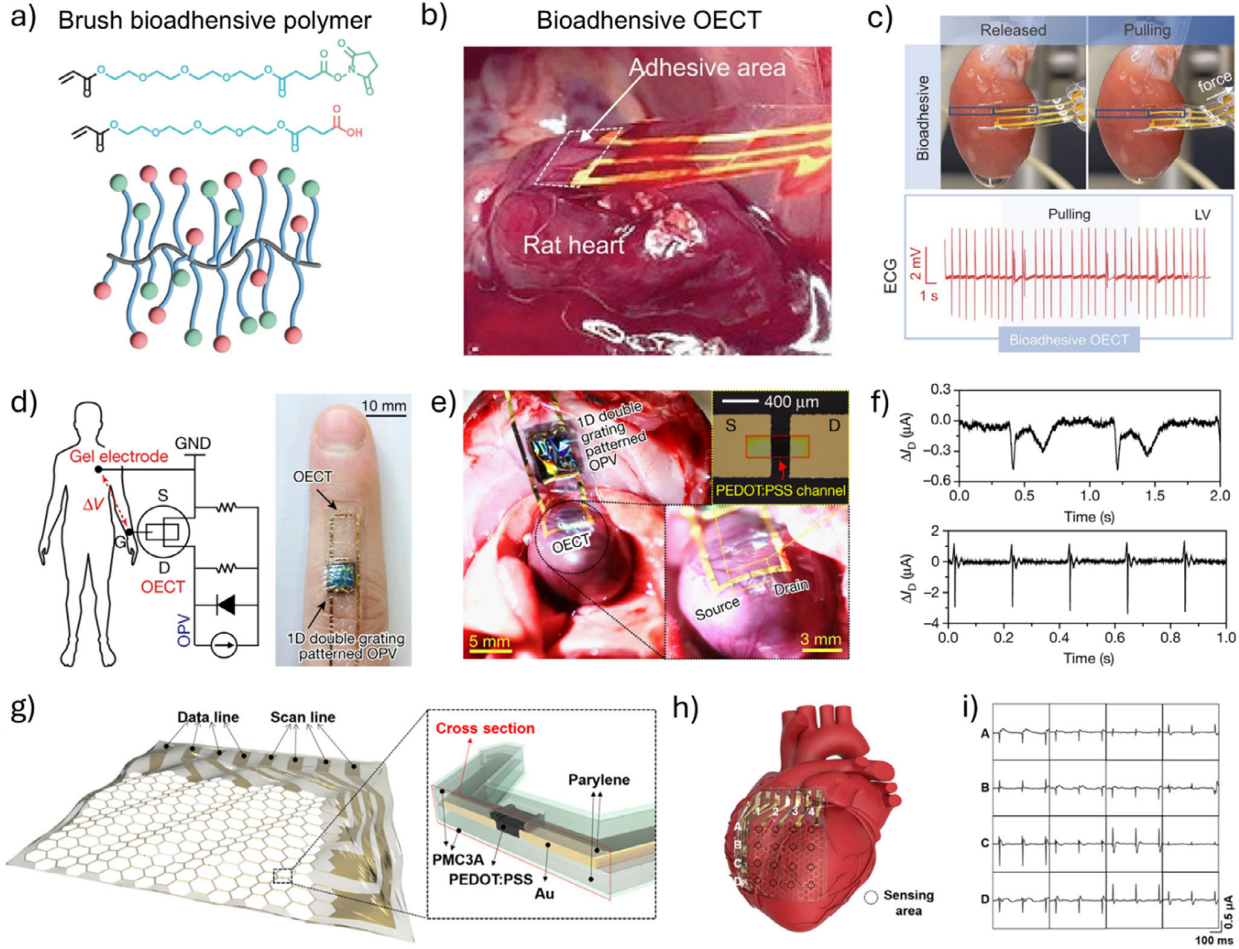
a) Chemical structures of adhesive monomers with elongated linear side chains terminated by NHS ester and COOH groups, and a schematic illustration of the resulting bioadhesive polymer (BAP); b) Photograph showing a fully bioadhesive OECT with adhered to a rat heart for ECG recording; c) Photographs of a fully bioadhesive OECT stably attached to an isolated rat heart surface during mechanical agitation (top) and corresponding ECG signals recorded by the device (bottom). a–c) Reproduced with permission.^[^ ^12^ ^]^ Copyright 2023, AAAS. d) Wiring diagram for cardiac signal recording; e) Photograph of the OECT‐integrated device attached to a finger and a rat heart. f) Measured output current from the recorded cardiac signal trace, under light illumination (top) and output current from the ECG trace, under light illumination (bottom). d–f) Reproduced with permission.^[^ ^209^ ^]^ Copyright 2018, Springer Nature. g) Photo of the OECT array on a honeycomb grid parylene substrate; h) Image of the positioning of a OECT array on the heart of a rat and the corresponding ECG signal shown in (i). g–i) Reproduced with permission.^[^ ^68^ ^]^ Copyright 2018, AAAS.

Typically, a flexible thin‐film device is attached directly onto the skin, such as forearm, chest, and wrist, or directly onto the heart to measure the ECG signals. The high dynamism of cardiac tissue, characterized by 40–200 contractions per minute and 10%–20% strain, places higher demands on the mechanical matching of ECG interfaces. Beyond the earlier principle of “ultra‐thin substrates combined with elastic materials”, interface adhesion must be further optimized to prevent detachment during cyclic contraction. To further enhance the interfacial coupling between OECTs and biological tissues, Li et al.^[^ ^12^ ^]^ proposed a novel strategy based on bioadhesive polymer semiconductors (BAP). This approach integrates a hydrogel‐inspired adhesive interface directly into the semiconductor architecture at the molecular level. Specifically, conjugated polymers were functionalized with *N*‐hydroxysuccinimide (NHS) ester and carboxylic acid (COOH) groups to construct a bioadhesive polymer semiconductor (BASC) with a double‐network architecture (**Figure** **16**a). NHS ester groups covalently bind to primary amines on tissue surfaces under moist conditions, while carboxylic acid groups enhance interface adhesion by absorbing interfacial moisture. This enables the polymer to adhere directly to wet tissue surfaces without the need for external adhesives, forming a stable and conformal bioelectronic interface. Such a “molecular interlocking” mechanism substantially improves mechanical stability and recording fidelity under moist, dynamic physiological conditions (Figure 16b). When adhered to the beating surface of an ex vivo rat heart, the device remained firmly attached even under mechanical perturbation and successfully recorded ECG signals with high fidelity during dynamic operation (Figure 16c). Notably, this group has recently made further advances in the intrinsic design of hydrogel‐based semiconductor materials, exploring integrated strategies that unify electronic–ionic coupling with interfacial mechanical functionality.^[^ ^138^ ^]^

While bioadhesive OECTs address the challenge of dynamic cardiac adhesion, the issue of stable power supplies remains, as traditional wired sources fail to withstand mechanical deformation. To address this, Park et al.^[^ ^209^ ^]^ developed a self‐powered, ultra‐flexible bioelectronic platform by integrating OECTs with an ultrathin organophotovoltaic (OPV) device. Built on a 1 µm‐thick parylene substrate, with an overall thickness of approximately 3 µm, this design enables intimate conformation to soft biological surfaces—including the dynamic surface of a beating rat heart—where it effectively recorded cardiac signals under LED illumination (Figure 16d–f)). Using a room‐temperature nanoimprint process, periodic nano‐grating structures (760 nm pitch) were formed on the charge transport layers, boosting the OPV's power conversion efficiency (PCE) to 10.5%, with a power‐to‐weight ratio of 11.46 W g^−1^. The integrated OECT achieved a transconductance of 0.8 mS and a response speed exceeding 1 kHz under physiological conditions, with cardiac signals showing a maximum SNR of 40.02 dB.

Beyond biocompatibility and seamless integration, enhancing spatial resolution and enabling multiplexed signal acquisition are key goals in advancing OECT‐based interfaces. In this regard, the team further applied the OECT array to more mechanically dynamic biological substrates—specifically, the surface of a beating heart.^[^ ^68^ ^]^ To ensure stable signal acquisition in wet, fluid‐rich, and constantly deforming physiological environments, the system employed a honeycomb‐structured parylene mesh to impart lateral stretchability, and was coated with a hemocompatible polymer, poly(3‐methoxypropyl acrylate) (PMC3A). The device, comprising a 4 × 4 OECT array, exhibited excellent flexibility and adhesion, allowing direct deployment onto dynamic cardiac tissue without any fixation (Figure 16d). Under continuous cardiac motion, the device recorded ECG signals with clearly defined P, QRS, and T waves, achieving a SNR of up to 52 dB while maintaining a stable transconductance exceeding 1 mS (Figure 16f). Moreover, the device showed minimal degradation in electrical performance under mechanical stretching. Even after 10^4^ repeated stretching cycles, output current fluctuations remained minimal, underscoring the device's mechanical robustness and its potential for long‐term deployment on highly curved visceral surfaces such as the heart or blood vessels.

**Figure 17:**
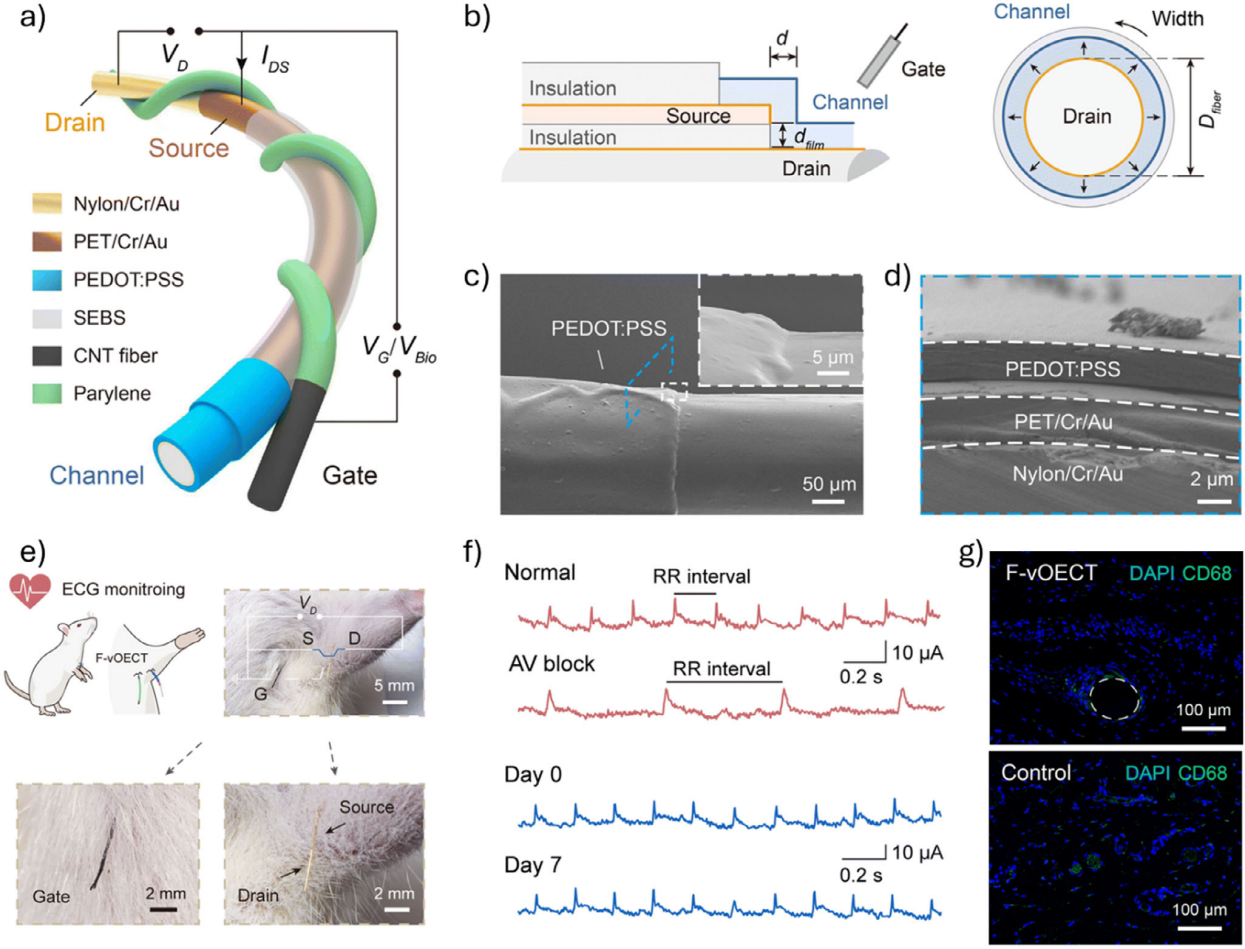
a) Schematic illustration of the configuration of the F‐vOECT; b) Schematic diagrams of the F‐vOECT cross‐section and profile; c) and d) SEM images of the F‐vOECT cross‐section and profile, respectively; e) Photograph of a rat implanted with F‐vOECT and the corresponding circuit diagram; f) ECG signals recorded by F‐vOECT (top), and comparative ECG recordings on day 0 and day 7 (bottom); g) Immunohistochemical staining images of rat subcutaneous tissue after 1‐week implantation of F‐vOECT (left) and non‐implanted control group (right). Reproduced with permission.^[^ ^207^ ^]^ Copyright 2024, Royal Society of Chemistry.

Fiber‐shaped vertical organic electrochemical transistors (F‐vOECTs) offer significant advantages for implantable bioelectronics.^[^ ^206^ ^]^ The cylindrical geometry of fiber devices enables smaller implantation footprints and omnidirectional mechanical compliance, which is beneficial for reducing tissue damage and improving long‐term integration stability. However, conventional fiber‐shaped OECTs typically rely on manually masked or electrolyte‐defined large channel areas, leading to slow response times that often exceed 100 ms,^[^ ^210^, ^211^, ^212^ ^]^ thus limiting their utility in capturing fast electrophysiological signals such as ECG and EMG. To address these challenges, Zhong et al.^[^ ^206^ ^]^ developed high‐performance fiber‐shaped vOECTs using a surface photolithography approach to precisely define short vertical channels on the fiber surface. This device architecture adopts a coaxial stacking configuration in which the drain and source electrodes are vertically aligned, and a p‐type semiconductor layer (p(g2T‐T)) is sandwiched between them. The resulting p‐type devices demonstrated outstanding electrical performance, including a maximum transconductance of up to 41.1 mS and a turn‐on transient time of approximately 138 ms. This performance significantly surpasses that of previous fiber‐based OECTs. Moreover, the vertical configuration reduces the channel length to around 3 µm, effectively decreasing the ion transport path and enhancing temporal resolution through reduced capacitive lag. Chen et al.^[^ ^207^ ^]^ employed a similar fabrication strategy to precisely define a vertical channel thickness of ∼3 µm between a 75 µm nylon/Cr/Au fiber and a PET/Cr/Au film source electrode (**Figure** **17**b–d), significantly shortening the ion transport path. The resulting F‐vOECT exhibited a high transconductance of 16 mS at 0 V gate bias. Moreover, under a minimum bending radius of 1.5 mm, after 500 repeated bending cycles, the variations in both transconductance and on‐state current remained within 10%. Upon subcutaneous implantation in rats (Figure 17e), the device successfully recorded real‐time ECG waveforms under both normal and atrioventricular (AV) block conditions (Figure 17f) top), with the RR interval extending from 0.2 to 0.55 s, and maintained stable signal amplitude over a period of 7 days (Figure 17f) bottom). In addition, histological analysis after 7 days of implantation showed no apparent inflammation or morphological abnormalities in H&E staining, confirming excellent tissue compatibility (Figure 17g).

#### 6.2.2 ECoG Sensing Interfaces

**Figure 18:**
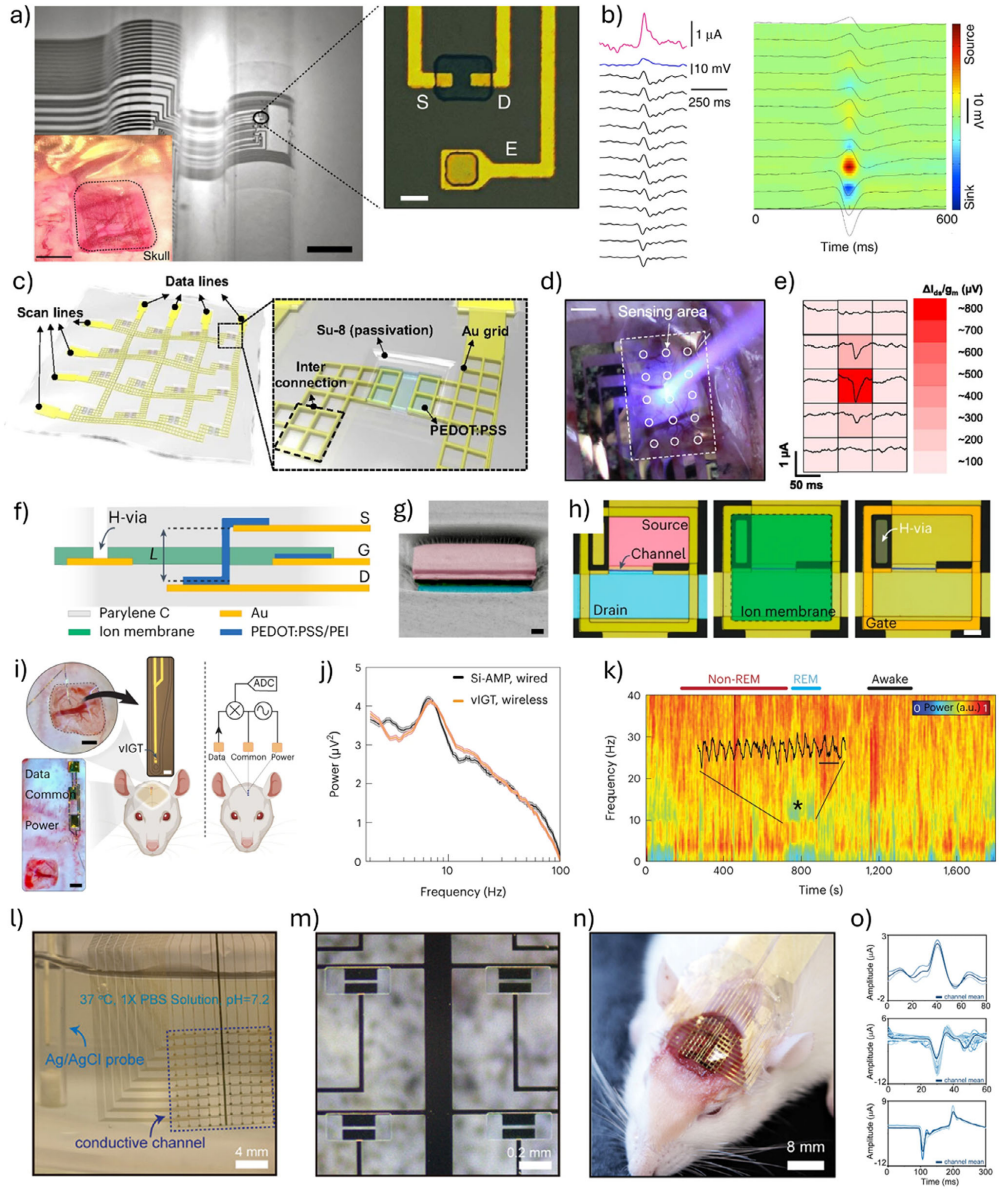
a) Optical micrograph of the device conforming onto a curvilinear surface. The inset shows the placement of the ECoG probe on the somatosensory cortex; b) Recording of a bicuculline‐induced epileptiform spike from a transistor (pink), a PEDOT:PSS surface electrode (blue) and 12 of the 16 Ir‐penetrating electrodes (black). a,b) Reproduced with permission.^[^ ^213^ ^]^ Copyright 2013, Springer Nature. c) Photo of the transparent electrophysiology OECT array on a parylene substrate; d) Photograph of the transparent electrophysiology array stimulated by continuous blue laser with e) indicating a strong ECoG signal measured by the OECT at the center of the array. c–e) Reproduced with permission.^[^ ^214^ ^]^ Copyright 2018, National Academy of Sciences. f) Cross‐sectional schematic of a vertical ion‐gated transistor (vIGT) with an H‐via enabling hydration through the ion membrane; g) SEM image of the channel region; h) Optical micrograph showing the top view of a single vIGT; i) Standalone vIGT device for in vivo recording and wireless transmission; j) Power spectral comparison between vIGT and conventional neural interface; k) Time–frequency spectrogram of neural data acquired by the vIGT‐based device. f–k) Reproduced with permission.^[^ ^208^ ^]^ Copyright 2023, Springer Nature. l) Optical image of a 100‐channel OECT array; m) Optical image of four‐unit cells after fabrication. n) Photograph of OECT array laminating onto the cerebral cortex of SD rat; o) Representative evoked spike waveforms under different conditions: anaesthesia (top), epileptic seizure (middle), and electrical stimulation (bottom). l–o) Reproduced with permission.^[^ ^75^ ^]^ Copyright 2023, Wiley.

ECoG is another technique used to measure the electrical activity of the brain. Unlike EEG, which involves placing electrodes on the scalp, ECoG requires the placement of electrodes directly on the brain's surface. This method provides higher spatial resolution and more precise localization of the source of electrical activity compared to EEG. To achieve this level of precision, ECoG sensors must possess specific characteristics such as biocompatibility, high spatial resolution, stability, low noise, and flexibility. These properties ensure accurate and reliable measurements. Given the low elasticity modulus of cortical brain tissue (0.2–7 kPa), ultra‐soft substrates like parylene are ideal, as they can seamlessly conform to the brain's curvature while ensuring high‐quality signal acquisition. For instance, Khodagholy et al.^[^ ^213^ ^]^ first demonstrated that OECTs can achieve high SNR in in vivo electrophysiological recordings. This was made possible by employing an ultrathin (2 µm) poly(p‐xylylene) (parylene) substrate, resulting in a total device thickness of only 4 µm, thereby enabling conformation to the brain's curvature (**Figure** **18**a). The integration of this ultra‐soft parylene film with a flexible PEDOT:PSS active layer allowed for stable interfacing with cortical tissue, enhancing the fidelity of low‐amplitude neural signals, including pathological oscillations observed in epilepsy models (Figure 18b). Similarly, Lee et al.^[^ ^214^ ^]^ developed a 3 × 5 OECT array using a 1.2 µm‐thick parylene substrate, combined with transparent Au nanomesh electrodes and a PEDOT:PSS active layer, resulting in a total device thickness of only ∼3 µm (Figure 18c). Each pixel possesses independent amplification capability, and by employing an active matrix design, high‐density interconnects are achieved without introducing significant crosstalk, thereby minimizing the number of external leads and improving system scalability. As shown in Figure 18d, the device was conformally laminated onto the cortex of an optogenetically modified rat, enabling precise mapping of cortical potentials under laser stimulation. Thanks to its high optical transparency and mechanical conformability, the laser can directly pass through the device, avoiding artifacts from light scattering or reflection. The OECT array exhibits rapid responses to localized stimulation, with a Δ*I*_ds_/*g*_m_ voltage signal of up to ∼800 µV at the center pixel, and a clearly resolved spatial distribution across neighboring pixels, demonstrating its high‐resolution capability for ECoG recording (Figure 18e). Each OECT unit within the array achieved a transconductance of 1.1 mS and a response time of < 350 µs, sufficient for tracking fast neural activity.

In addition to the advances in conventional OECTs for ECoG sensing, recent research has focused on vertical OECTs (vOECTs), which minimize in vivo volume occupation by orienting the channel perpendicular to the substrate—addressing the spatial constraints of cortical brain tissue. This design not only reduces the device footprint but also improves integration with the curved cerebral cortex, making it a promising direction for high‐resolution ECoG recording. For instance, Cea et al.^[^ ^208^ ^]^ proposed a vertical internal ion‐gated transistor (vIGT) architecture, which incorporates a miniaturized hydration conduit (H‐via) within the vOECT stack (Figure 18f). This design enables channel operation without direct contact with external electrolytes, thereby mitigating crosstalk in dense arrays while maintaining stable hydration (Figure 18g,h). The vIGTs exhibited ultrafast response times (τ ≈ 0.9 µs), sustained operational stability for over a year in physiological media, and achieved integration densities exceeding 150 000 transistors per cm^2^. Notably, the authors realized a fully self‐contained, wirelessly powered neural interface based entirely on vIGTs, enabling real‐time acquisition and transmission of electrocorticographic (ECoG) signals in freely moving rodents (Figure 18i). The system successfully captured somatosensory‐evoked potentials and chronic neural activity with signal fidelity comparable to that of conventional wired silicon‐based systems (Figure 18j,k).

#### 6.2.3 Other Electrical Signal

**Figure 19:**
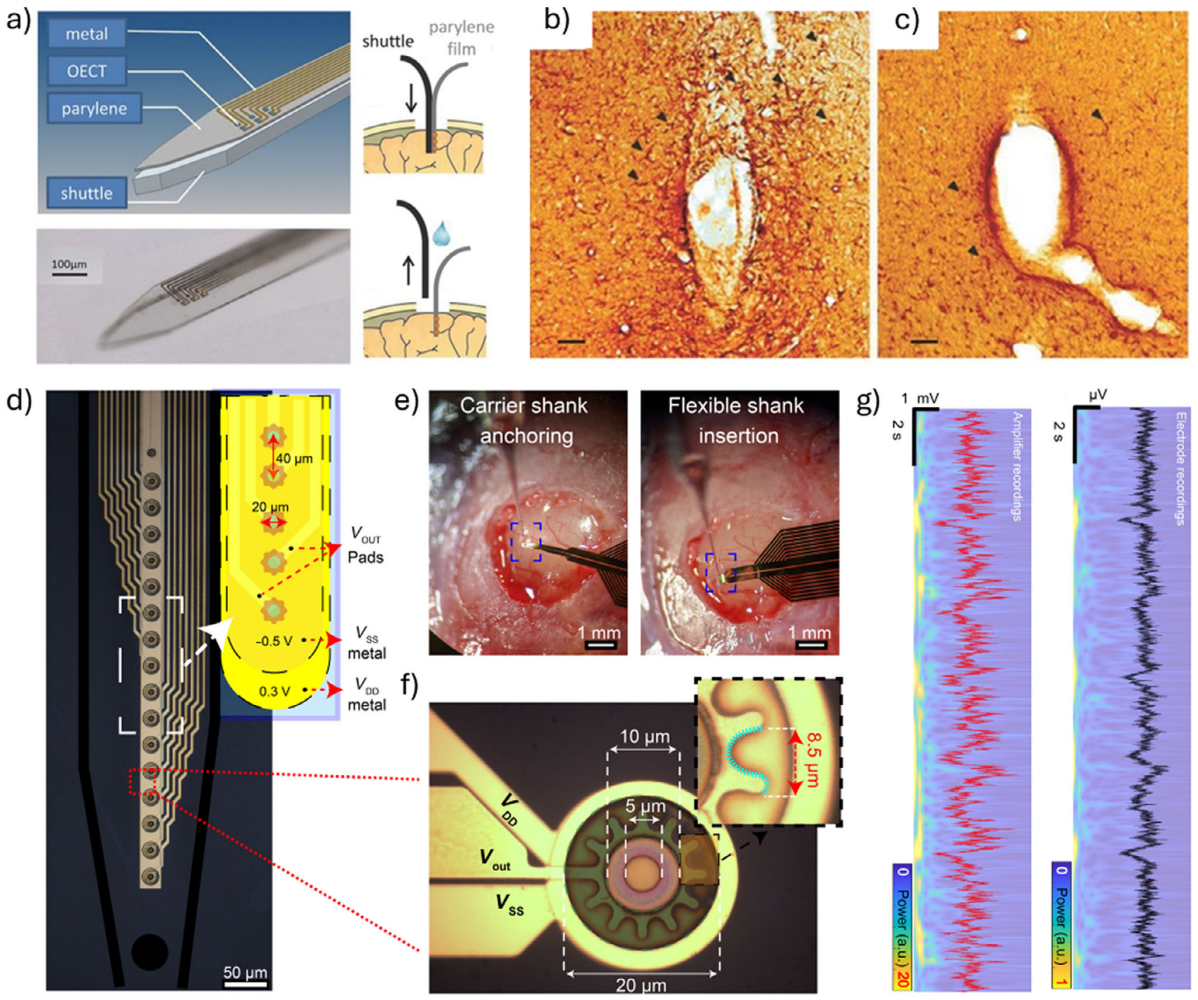
a) Photo of parylene‐ and the shuttle‐parts of the entire probe (left) and principle of delamination (right); Glial scarring observed around b) standard silicon probe and c) delaminating depth probe after one month of implantation. a–c) Reproduced with permission.^[^ ^67^ ^]^ Copyright 2015, Wiley. d) Image of the shank comprising 16 push‐pull amplifiers, with the array layout shown in the right inset. e) In vivo implantation procedure of the flexible shanks using a rigid guide; f) Image of an individual push–pull amplifier, with n‐channel (green) and p‐channel (purple) shown; g) Comparison of in vivo activity recorded by the amplifiers (red) versus the passive electrode (blue). d–g) Reproduced with permission.^[^ ^216^ ^]^ Copyright 2024, AAAS.

Beyond ECG and ECoG signals, in vivo OECTs also enable sensing of deep neural signals and neuromuscular signals—electrical signals that require adaptations to the “mechanical matching” and “minimally invasive” criteria for non‐superficial tissues. These signals, such as single‐neuron spikes in deep brain regions, demand higher spatial resolution and smaller device footprints than ECG or ECoG, driving innovations in flexible shank arrays and vertical amplification architectures. As early as ten years ago, Williamson et al.^[^ ^67^ ^]^ reported the synthesis of an OECT on a 4 µm parylene film, which was attached to a rigid SU‐8 shuttle via van der Waals forces. The device was then accurately positioned within the brain using a needle implantation method. The moisture in cerebrospinal fluid caused the parylene film to detach from the shuttle, leaving only the flexible film embedded within the brain tissue (Figure 19a). This implantation method resulted in no significant glial scarring around the device after one month in the rat cortex (Figure 19c). In contrast, traditional silicon probes induced prominent inflammatory responses (Figure 19b). This outcome is attributed to the small size of the rigid shuttle, which minimizes the wound size, and the excellent biocompatibility of parylene with biological tissue.

Similarly, Uguz et al.^[^ ^215^ ^]^ leveraged the flexibility of the same substrate to fabricate a 32‐channel shank array, with each pixel having a diameter of only 26 µm, resulting in a fourfold increase in channel density compared to the design by Williamson et al.^[^ ^67^ ^]^ The device's cross‐sectional area is just 50 × 8 µm^2^, with a total thickness of 8 µm, allowing the device to be inserted with minimal tissue disruption. More importantly, this study utilized p‐n OED technology, combining p–n diodes with OECTs, which significantly enhanced the device's rectification ratio (10^5^) and switching speed (230 µs). This advancement minimizes inter‐channel crosstalk during time‐division multiplexing, reducing it by 66 dB compared to single OECTs. Around the same time, the team demonstrated efficient front‐end signal amplification by integrating complementary p‐type and n‐type OECTs.^[^ ^216^ ^]^ By utilizing both enhancement‐mode and depletion‐mode devices, they developed an amplifier architecture with a small‐signal voltage gain exceeding 30 dB and a common‐mode rejection ratio (CMRR) greater than 60 dB. Each amplifier pixel measured only 20 µm in diameter with a total device thickness of 8 µm (Figure 19d). They implemented a vertical push‐pull topology in which sawtooth‐shaped n‐type electrodes expanded the effective channel width, and rectangular p‐type electrodes were designed to match the transconductance (Figure 19f). This configuration allowed the integration of n‐type p(C₆NDI‐T) and p‐type p(g2T‐TT) into compact vertical units. The channel length was defined by the thickness of the parylene C insulating layer, which enabled efficient rectification and fast switching performance. During the implantation, a rigid guide controlled by a micromanipulator was used to insert the flexible shank into the mouse cortex. After insertion, hydration by cerebrospinal fluid detached the carrier, leaving only the flexible amplifier array embedded in the tissue (Figure 19e). This method reduced the cranial opening to 300 µm in diameter, much smaller than the openings required for conventional silicon probes (> 3 mm) and helped lower the risk of glial scarring due to physical injury. In vivo recordings confirmed the performance advantages of the push‐pull amplifier array. Compared with passive electrodes, the amplifiers exhibited significantly higher SNR and revealed additional features in the recorded signals, including *δ* and *θ* band oscillations and single‐neuron spikes above 300 Hz (**Figure** **19**g). Through high rectification ratios that suppress crosstalk and a push–pull configuration that cancels common‐mode noise, the system was able to amplify low‐amplitude neural signals into the millivolt range.

### 6.3 In Vivo Biochemical Signals

In the field of implantable OECTs, in vivo chemical sensing applications remain relatively limited compared to electrical signal recording. This is primarily driven by the core design concept of wearable/implantable bioelectronics: most devices prioritize non‐invasive or minimally invasive detection to enhance user comfort and compliance, even at the cost of partial accuracy. For chemical sensing, non‐invasive approaches (e.g., sweat‐based glucose monitoring via skin‐mounted OECTs) are more favored in clinical and daily health management, as they avoid the risks of tissue damage or infection associated with invasive implantation. In contrast, in vivo electrical sensing (e.g., EEG, myocardial electricity) has matured faster—implantable OECTs excel at capturing weak, dynamic bioelectric signals through their high transconductance and conformal interfaces, making them the primary focus of early in vivo applications.

**Figure 20:**
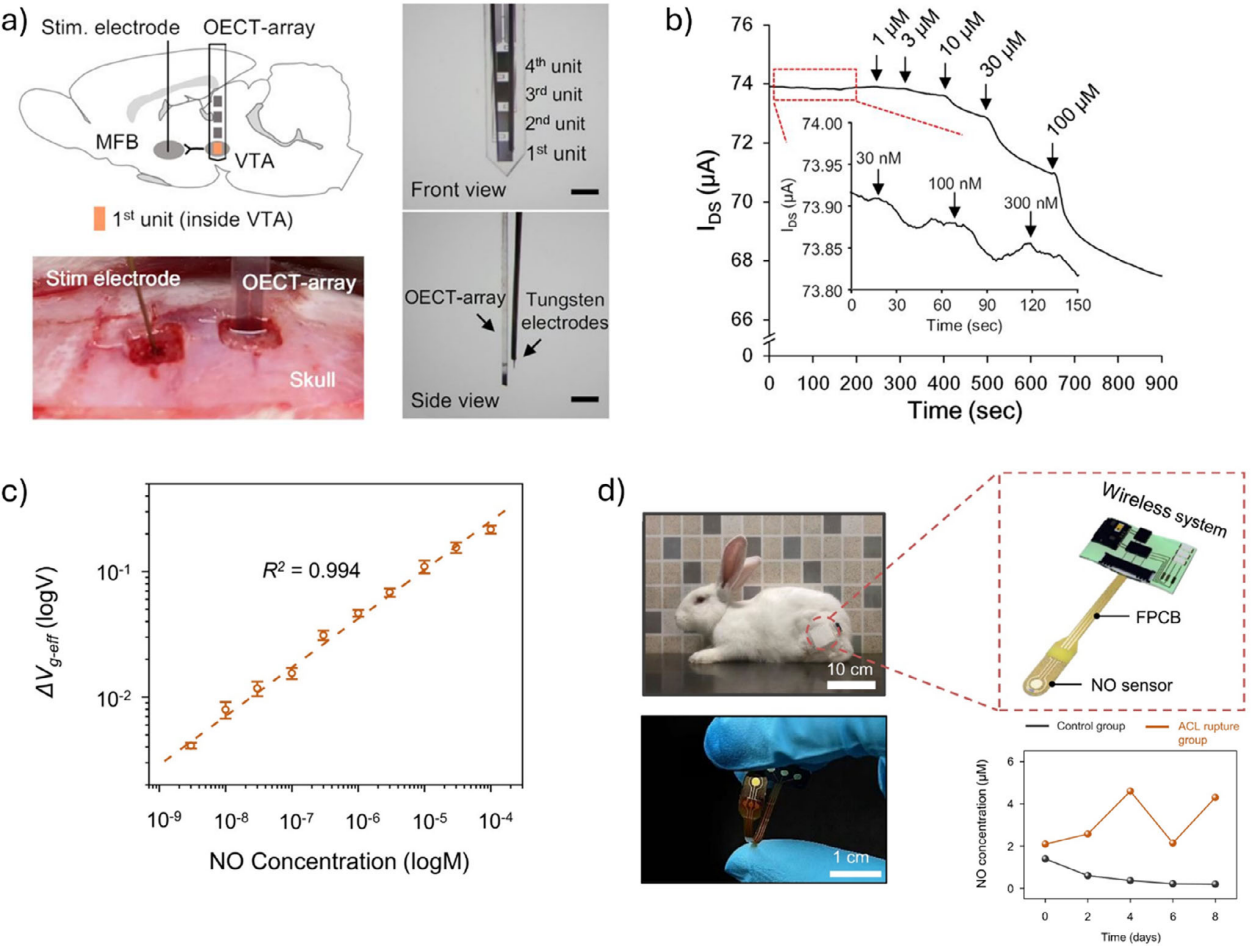
a) Schematic diagram (upper) and surgical photogram (lower) of the experimental setup; b) Ex vivo recording of IDS changes in response to artificially added dopamine to ACSF containing a high level of ascorbate acid. a,b) Reproduced with permission.^[^ ^217^ ^]^ Copyright 2021, eLife Sciences Publications, Ltd. c) Calibration curve showing the relationship between the effective gate voltage changes ΔVg‐eff and NO concentrations in logarithmic scale. d) Flexible high‐sensitivity NO sensing platform scheme. Bottom right corner shows the joint cavity of New Zealand rabbits without and ACL rupture within 0, 2, 4, 6, 8d above the concentration of no in the joint cavity. c,d) Reproduced with permission.^[^ ^218^ ^]^ Copyright 2022, National Academy of Sciences.

Nevertheless, recent advances in OECT interface engineering (e.g., molecular functionalization, miniaturized array design) have enabled breakthroughs in implantable chemical sensing, addressing the need for localized, real‐time detection of endogenous biomolecules (e.g., neurotransmitters, metabolites) that cannot be captured by non‐invasive methods. For example, a pioneering work by Xie et al.^[^ ^217^ ^]^ reported the first fully implantable OECT array for real‐time, multi‐site detection of catecholamine neurotransmitters (CA‐NTs, including dopamine, noradrenaline) in the brains of living rats—filling a critical gap in implantable OECT chemical sensing. The device was engineered to balance mechanical compliance, biocompatibility, and molecular recognition: it featured a slim blade‐shaped architecture (∼1 mm wide, ∼15 mm long, ∼200 µm thick) on a polyethylene terephthalate (PET) substrate, with 4 independent OECT units (spaced 1.2 mm apart) to cover distributed brain regions (**Figure** **20**a). Each unit consisted of a Pt gate electrode (for molecular recognition) and a PEDOT:PSS channel (for signal amplification), with all non‐sensing areas insulated by SU‐8 photoresist to prevent interference from the complex brain microenvironment. The chemical sensing mechanism relied on a functionalized interface at the Pt gate: CA‐NTs released into the extracellular space undergo electro‐oxidation—their catechol groups are converted to quinones, transferring electrons to the Pt gate to modulate the effective gate voltage (*Δ*V_eff_) and subsequent channel current (*I*_ds_). This molecular recognition process was built on a stable physical interface: the PET substrate's low modulus (∼2.8 GPa) and ultra‐thin design enabled conformal adhesion to curved brain surfaces, while PEDOT:PSS's intrinsic biocompatibility minimized foreign body responses. The device achieved a detection limit of 1 nM for dopamine in phosphate‐buffered saline (PBS) and 30 nM in artificial cerebrospinal fluid (ACSF) (containing 1.28 mM ascorbate, a major in vivo interference), with a temporal resolution of ∼50 ms—sufficient to capture phasic neurotransmitter release (Figure 20b).

Another representative advance in implantable OECT chemical sensing is Deng et al.’s flexible, wireless sensor for real‐time nitric oxide (NO) detection in joint cavities—targeting post‐traumatic osteoarthritis (OA) monitoring, where NO acts as a key inflammatory biomarker.^[^ ^218^ ^]^ This sensor uses a flexible polyimide substrate (matching joint tissue compliance) with a miniaturized PEDOT:PSS channel and an Au gate modified with poly‐5A1N (a NO‐selective membrane). The poly‐5A1N layer blocks interfering molecules (e.g., glucose, uric acid) while boosting NO oxidation, enabling a 3 nM detection limit and 3 nM–100 µM linear range (Figure 20c). A 5 µm SU‐8 encapsulation ensures biocompatibility, and a Bluetooth module supports wireless data transmission. In vivo tests on rabbits with unilateral ACL rupture showed: the ACL group (injured joint) maintained ∼3–4 µM NO for 8 days (versus ∼0.5 µM in uninjured controls), and this high NO correlated with later OA signs (disordered chondrocytes, damaged cartilage) (Figure 20d). The sensor retained 82.1% of initial response after 10 days, with no significant foreign body reactions.

## 7 Conclusion and Outlook

OECTs have emerged as transformative bioelectronic platforms capable of interfacing electronic and biological systems by virtue of their ionic–electronic coupling, mechanical compliance, and biocompatibility. As outlined in this review, significant progress in materials engineering, device architectures, and interface strategies has greatly enhanced the ability of OECTs to function within soft, dynamic, and heterogeneous biological environments. These advances have enabled high transconductance, sub‐millisecond response times, and stable performance in both wearable and implantable applications. As a result, OECTs have demonstrated potential across diverse biomedical uses, including electrophysiological signal acquisition, single‐cell analysis, and real‐time biosensing.

Despite these promising developments, several key challenges must be addressed before OECTs can be widely adopted in clinical settings. One major concern involves the long‐term biostability and compatibility of organic materials within the body. Although PEDOT:PSS is currently the most commonly used organic mixed ionic–electronic conductor (OMIEC), its degradation behavior under physiological conditions remains insufficiently understood. Common issues include oxidative damage, ion leaching, and biofouling.^[^ ^219^, ^220^, ^221^ ^]^ In addition, sterilization processes such as autoclaving and gamma irradiation can impair the electrical and structural integrity of both OMIECs and encapsulation materials.^[^ ^73^ ^]^ Future research should therefore focus on developing next‐generation OMIECs that combine high performance with properties such as bioresorbability, immune neutrality, and resistance to enzymatic and mechanical degradation. Emerging solutions include dynamic covalent networks, zwitterionic backbones, and supramolecular assemblies that exhibit self‐healing and antifouling characteristics.

Another critical challenge in the development of OECTs lies in the development of high‐performance channel materials, particularly stretchable polymer semiconductors. A central challenge lies in maintaining effective charge transport under mechanical deformation. In dynamic biological environments, devices experience continuous bending, stretching, and twisting. These mechanical stresses can significantly disrupt the molecular ordering and π‐conjugation of the polymer backbone, thereby resulting in diminished conductivity. Although a comprehensive review has outlined molecular design strategies such as the incorporation of flexible side chains, crosslinkable units, and dynamic covalent networks,^[^ ^222^ ^]^ translating these design rules into OECT‐compatible materials remains challenging, and systematic investigations into the stretchability of OECT channel materials are scarce. Most current research prioritizes electrochemical performance or charge mobility, frequently overlooking mechanical robustness under mechanical strain. Achieving a balance between mechanical durability and efficient ionic and electronic transport requires innovative materials engineering strategies. One promising approach is the hybridization of conductive polymers with nanoscale fillers such as carbon nanotubes,^[^ ^223^ ^]^ graphene oxide (GO),^[^ ^224^ ^]^ or MXenes,^[^ ^225^ ^]^ or incorporation of ion‐conductive hydrogels to enhance mechanical strength and maintain percolated charge pathways under strain. However, meticulous optimization of the filler content is essential. Excessive incorporation of rigid fillers can disrupt the continuity of the polymer matrix, resulting in phase separation, diminished stretchability, and impaired ion mobility. Furthermore, beyond a critical threshold, interfacial mismatch and agglomeration of fillers may degrade both the electrical conductivity and mechanical compliance of the composite, ultimately compromising device performance. Therefore, precise control over the type, dispersion, and loading of fillers is essential for the systematic development of next‐generation stretchable OECT channel materials.

In addition to the aforementioned laboratory‐scale challenges, a key hurdle for broad clinical and commercial applications lies in the repeatability and uniformity of the fabrication process. Although current techniques, including spin coating, inkjet printing,^[^ ^226^ ^]^ and soft lithography,^[^ ^155^ ^]^ exhibit promising scalability, ensuring uniformity in material deposition, device alignment, and performance across large‐area substrates requires further refinement. For example, the performance consistency of PEDOT:PSS‐based OECTs fabricated using screen printing technology exhibits significant variation across different substrate locations.^[^ ^227^, ^228^ ^]^ Recent advancements in additive manufacturing,^[^ ^172^, ^229^ ^]^ continuous wet‐spinning processing,^[^ ^230^ ^]^ and photopatternable OMIECs^[^ ^231^ ^]^ provide promising solutions to address these challenges. Notably, a hybrid process combining orthogonal photolithography and inkjet printing has been validated to integrate the high precision of lithography (±1 µm lateral tolerance for PEDOT:PSS channels) and the uniform thickness control of printing (<5% deviation for solid‐state electrolytes, SSEs).^[^ ^232^ ^]^ While these advancements offer promising solutions, future research should focus on optimizing fabrication processes for large‐scale production, ensuring consistency across various device types and environmental conditions.

Beyond technical advances, clinical translation requires robust regulatory and standardization frameworks. To date, no dedicated ISO or FDA protocols exist for evaluating the safety and efficacy of organic bioelectronic devices, particularly those involving degradable polymers or skin‐conformal designs. While some recent guidelines address general biocompatibility and material safety, they fall short of addressing the specific challenges posed by these systems. Tailored standards assessing electrical performance, mechanical durability, immune response, and degradation under physiological conditions are urgently needed to ensure device reliability and patient safety. Finally, the successful implementation of OECT technologies will benefit from close collaboration across multiple sectors. Coordination among academic researchers, industry developers, healthcare professionals, patients, and regulatory authorities is critical for transforming laboratory innovations into clinically applicable solutions. Shared benchmarking platforms, open‐access physiological datasets, and interdisciplinary research consortia will help accelerate development and facilitate broader adoption of OECTs in biomedical applications.

## Conflict of Interest

The authors declare no conflict of interest.
